# The *Amborella* vacuolar processing enzyme family

**DOI:** 10.3389/fpls.2015.00618

**Published:** 2015-08-21

**Authors:** Valérie Poncet, Charlie Scutt, Rémi Tournebize, Matthieu Villegente, Gwendal Cueff, Loïc Rajjou, Thierry Balliau, Michel Zivy, Bruno Fogliani, Claudette Job, Alexandre de Kochko, Valérie Sarramegna-Burtet, Dominique Job

**Affiliations:** ^1^Institut de Recherche pour le Développement, UMR Diversité, Adaptation et Développement des PlantesMontpellier, France; ^2^Laboratoire Reproduction et Développement des Plantes, UMR 5667, Ecole Normale Supérieure de LyonLyon, France; ^3^Laboratoire Insulaire du Vivant et de l'Environnement, Université de la Nouvelle-CalédonieNouméa, New Caledonia; ^4^Institut National de la Recherche Agronomique, Institut Jean-Pierre Bourgin, UMR 1318 Institut National de la Recherche Agronomique/AgroParisTech, ERL Centre National de la Recherche Scientifique 3559, Laboratoire d'Excellence “Saclay Plant Sciences” (LabEx SPS), RD10Versailles, France; ^5^AgroParisTech, Chaire de Physiologie VégétaleParis, France; ^6^Institut National de la Recherche Agronomique, Plateforme d'Analyse Protéomique de Paris Sud-Ouest, Institut National de la Recherche Agronomique/Université Paris-Sud/Centre National de la Recherche Scientifique/AgroParisTech, UMR 0320/UMR 8120 Génétique Quantitative et Evolution – Le MoulonGif-sur-Yvette, France; ^7^Institut Agronomique Néo-Calédonien, Diversités Biologique et Fonctionnelle des Ecosystèmes TerrestresPaïta, New Caledonia; ^8^UMR 5240 Laboratoire Mixte Centre National de la Recherche Scientifique/Institut National des Sciences Appliquées/Université Claude Bernard Lyon 1/Bayer CropScienceLyon, France

**Keywords:** *Amborella trichopoda*, vacuolar processing enzymes, seed, proteomics, plant evolution, genetic diversity

## Abstract

Most vacuolar proteins are synthesized on rough endoplasmic reticulum as proprotein precursors and then transported to the vacuoles, where they are converted into their respective mature forms by vacuolar processing enzymes (VPEs). In the case of the seed storage proteins, this process is of major importance, as it conditions the establishment of vigorous seedlings. Toward the goal of identifying proteome signatures that could be associated with the origin and early diversification of angiosperms, we previously characterized the 11S-legumin-type seed storage proteins from *Amborella trichopoda*, a rainforest shrub endemic to New Caledonia that is also the probable sister to all other angiosperms (*Amborella* Genome Project, 2013). In the present study, proteomic and genomic approaches were used to characterize the VPE family in this species. Three genes were found to encode VPEs in the *Amborella's* genome. Phylogenetic analyses showed that the *Amborella* sequences grouped within two major clades of angiosperm VPEs, indicating that the duplication that generated the ancestors of these clades occurred before the most recent common ancestor of living angiosperms. A further important duplication within the VPE family appears to have occurred in common ancestor of the core eudicots, while many more recent duplications have also occurred in specific taxa, including both *Arabidopsis thaliana* and *Amborella*. An analysis of natural genetic variation for each of the three *Amborella* VPE genes revealed the absence of selective forces acting on intronic and exonic single-nucleotide polymorphisms among several natural *Amborella* populations in New Caledonia.

## Introduction

Evolutionary genetics is considered as a central part of biology (Charlesworth and Charlesworth, 2009). In plants, *Amborella trichopoda (Amborella)*, an understory shrub endemic to New Caledonia, has been proposed to correspond to the single living representative of the sister lineage to all other extant flowering plants (Bremer et al., 2009; Jiao et al., 2011; Lee et al., 2011; Wickett et al., 2014). Hence the recent release of its genome sequence provides a pivotal reference for understanding genome and gene family evolution throughout angiosperm history (*Amborella* Genome Project, 2013).

In previous work (*Amborella* Genome Project, 2013), we characterized the *Amborella* seed storage proteins with the goal of identifying proteome signatures that could be associated with the origin and early diversification of angiosperms. In particular, we focused our attention on the abundant 11S globulins that have been characterized and compared across seed plants in evolutionary analyses (Häger et al., 1995; Adachi et al., 2003; Li et al., 2012). We found that the *Amborella* genome contains three distinct 11S globulin genes (*Amborella* Genome Project, 2013). In all plant species, 11S globulins are synthesized in the form of high molecular weight precursors that are processed by vacuolar processing enzymes (VPEs) during seed maturation. This limited proteolysis, which is regularly directed to an Asn-Gly (N-G) junction, yields the A (acidic)- and B (basic)-subunits of mature 11S globulins that is accompanied by further assembly of the trimer precursor-protein complexes into mature hexamers within the protein storage vacuoles (PSVs) (Chrispeels et al., 1982; Müntz, 1998; Shutov et al., 2003).

Although two of the three *Amborella* 11S globulins do contain a canonical N-G cleavage site, we observed that a third one deviates notably from the two others as it exhibits, in place of an N-G junction, an N-V-I sequence (*Amborella* Genome Project, 2013). Similar deviations from the N-G cleavage motif were observed for 11S globulins from *Ginkgo biloba* (*Amborella* Genome Project, 2013) and *Metasequoia glyptostroboides* (Häger and Wind, 1997), thus highlighting the possibly ancestral nature of this atypical *Amborella* 11S globulin.

Most vacuolar proteins (as is the case for the 11S globulins) are synthesized on the rough endoplasmic reticulum (ER) as proprotein precursors and then transported to the vacuoles where they are converted into their respective mature forms (Neuhaus and Rogers, 1998; Herman and Larkins, 1999) by the action of VPEs (EC 3.4.22.34). VPEs, also called legumains or asparaginyl endopeptidases, are cysteine proteases found in various organisms, including plants, mammals, and protozoans such as *Schistosoma mansoni*. They are classified as members of family C13 in the MEROPS protease database (Rawlings et al., 2008; http://merops.sanger.ac.uk/) that belongs to the CD clan, which also contains caspases (family C14A) and metacaspases (family C14B) (Misas-Villamil et al., 2013). Caspases are the main players in the regulation of programmed cell death (PCD) in animals, whereas metacaspases are involved in the same process in plants and fungi (Hara-Nishimura and Hatsugai, 2011; Tsiatsiani et al., 2011, 2012). Clan CD proteases contain a His–Cys catalytic dyad and have strict substrate requirements for the amino acid preceding the cleavable bond (P1 position) (Chen et al., 2004; Dall and Brandstetter, 2013; Misas-Villamil et al., 2013).

Plant VPEs are classified into vegetative and seed-expressed types (Gruis et al., 2002; Ariizumi et al., 2011; Radchuk et al., 2011; Julián et al., 2013; Kumar et al., 2015). The *Arabidopsis thaliana* (*Arabidopsis*) genome contains four VPE genes designated as α-VPE, β-VPE, γ-VPE, and δ-VPE (Rojo et al., 2003; Nakaune et al., 2005; Hatsugai et al., 2015). The seed-type β-VPE is essential for the proper processing of storage proteins (Shimada et al., 2003).

The co-existence in *Amborella* seeds of the angiosperm- and gymnosperm-type 11S globulins prompted us to characterize the VPE system in seeds of this plant. Here, we refine our understanding of this gene family with the characterization of several *Amborella* VPE homologs.

Phylogenetic analyses of plant VPEs and legumains have been previously reported. However these previous studies only considered selected sequences from monocots and eudicots and did not include sequences from gymnosperms or basal eudicots (Kato et al., 2003; Nakaune et al., 2005; Julián et al., 2013; Kang et al., 2013; Christoff et al., 2014; Pierre et al., 2014). To gain further insight in plant VPEs and benefiting from the present *Amborella* sequences, we reconstructed a phylogeny of VPE proteins based on the amino acid sequences of VPEs from a wide range of embryophytes (land plants). By using a comparative approach, combined with the principle of parsimony, data from this uniquely-placed angiosperm can help defining the condition of any character in the most recent common ancestor (MRCA) of the living angiosperms, and we have applied this method to the structural and functional evolution of the VPE family.

Another way to evaluate the functional relevance of genes is to examine the levels of naturally occurring genomic variations therein, i.e., polymorphism within populations (Koornneef et al., 2004). For this purpose we used next-generation sequencing data from the recently completed *Amborella* genome (*Amborella* Genome Project, 2013) to characterize single-nucleotide polymorphisms (SNPs) in VPE sequences and their distribution over the natural range distribution of *Amborella* in New Caledonia (Poncet et al., 2013).

## Materials and methods

### Plant material

Mature drupes of *Amborella* were collected from 10 individual trees located at “plateau de Dogny-Sarraméa” (New Caledonia; 21°37′0″ N, 165°52′59″ E). The fleshy part of the fruits was removed and pits (containing the seeds) were briefly dried on paper before being removed for seed isolation *stricto sensu*. Surface-sterilized seeds were cut longitudinally in two with a razor blade. A drop of sterile Milli-Q water was placed on the endospermic face of each half. Embryos were quickly extracted (in < 1 min), with clean extra-thin needles and immediately frozen in liquid nitrogen.

### Preparation of soluble protein extracts

For the preparation of soluble protein extracts, 100 isolated *Amborella* embryos were ground in liquid nitrogen using a mortar and pestle. Total soluble proteins were extracted at room temperature in 400 μl thiourea/urea lysis buffer composed of 7 M urea, 2 M thiourea, 6 mM Tris-HCl, 4.2 mM Trizma® base (Sigma-Aldrich, Lyon, France), 4% (w/v) 3-[(3-cholamidopropyl) dimethylammonio]-1-propanesulfonate (CHAPS, Sigma-Aldrich) supplemented with 50 μl of the protease inhibitor cocktail Complete Mini (Roche Diagnostics France, Meylan, France). Then, 15 μl of 1 M dithiothreitol (DTT, Sigma-Aldrich), 2 μl of DNase I (Roche Diagnostics), and 5 μl of RNase A (Sigma-Aldrich) were added to the sample. Following stirring for 2 h at 4°C, protein extracts were centrifuged at 20,000 g at 4°C for 15 min. The resulting supernatant was submitted to a second clarifying centrifugation, as above (Rajjou et al., 2011). The final supernatant was kept and protein concentrations in the various extracts were measured according to Bradford (1976) using Bovine Serum Albumin as a standard.

### Shotgun proteomic analysis

The *Amborella* seed proteome exploration was performed by LC-MS/MS analysis following preparation of soluble protein extracts (30 μg protein; *n* = 3 biological replicates) that had been subjected to 1D-SDS-PAGE (http://pappso.inra.fr). Protein extracts were loaded in 1X Laemmli buffer (Laemmli, 1970) with DTT (50 mM) in a stacking gel [acrylamide 8% (w/v); Tris-HCl 0.56 M, pH 8.8; SDS 0.1% (w/v)]. After 15 min of migration at 10 mA, the gel was stained with colloidal blue (GelCode Blue Stain Reagent; Thermo Fisher Scientific Inc, Rockford, IL) and destained in Milli-Q water. The whole band corresponding to total proteins was excised and submitted to in-gel digestion with the Progest system (Genomic Solution, Huntingdon, UK) according to a standard trypsin protocol. Briefly, gel pieces were washed for 1 h at 37°C in a solution containing 25% (v/v) acetonitrile (ACN) and 50 mM ammonium bicarbonate (pH 7.8), followed by dehydration in 100% ACN for 15 min. Gel pieces were rehydrated overnight at 37°C with 1/50 (w/w) trypsin (Promega, Madison, WI, USA) in 20 mM ammonium bicarbonate, pH 7.8. Digestion was stopped by adding 0.4% (v/v) of trifluoroacetic acid (TFA).

HPLC was performed on a NanoLC-Ultra system (Eksigent). A 4-μl sample was loaded at 7.5 μl/min^−1^ on a precolumn cartridge (stationary phase: BIOSPHERE C18, 5 μm; column: 100 μm i.d., 2 cm; NanoSeparations) and desalted with 0.1% methanoic acid (HCOOH). After 3 min, the precolumn cartridge was connected to the separating PepMap C18 column (stationary phase: BIOSPHERE C18, 3 μm; column: 75 μm i.d., 150 mm; NanoSeparations). Buffers used were 0.1% HCOOH in water (A) and 0.1% HCOOH in ACN (B). Peptide separation was achieved with a linear gradient from 5 to 30% B for 30 min at 300 nl/min^−1^. Including the regeneration step at 95% B and the equilibration step at 95% A, one run took 45 min. Eluted peptides were analyzed on-line with a Q-Exactive mass spectrometer (Thermo Electron) using a nano-electrospray interface (non-coated capillary probe, 10 μ i.d.; New Objective). Xcalibur 2.1 interface was used to monitor data-dependent acquisition of peptide ions (http://www.thermoscientific.com/content/tfs/en/product/xcalibur-software.html). This acquisition included a full MS scan covering 300 to 1400 range of mass-to-charge ratio (m/z) with a resolution of 70,000 and a MS/MS step (normalized collision energy: 30%; resolution: 17,500). MS/MS step was reiterated for the eight major ions detected during full MS scan. Dynamic exclusion was set to 45 s.

A database search was performed with XTandem (version 2011.12.01.1) (Bjornson et al., 2008; http://www.thegpm.org/TANDEM/) for protein identification. Enzymatic cleavage was declared as a trypsin digestion with one possible miscleavage. Cys carbamidomethylation and Met oxidation were declared, respectively as fixed and variable modifications. Precursor mass and fragment mass tolerance were 10 ppm and 0.02 Th, respectively. The *Amborella* Genome database (http://www.amborella.org/) and a contaminant database (trypsin, keratins) were used. Identified proteins were analyzed using XTandem Pipeline (http://pappso.inra.fr/bioinfo/xtandempipeline/, version 3.3). Only peptides with an E-value smaller than 0.03 were validated.

### Phylogenetic analyses

VPE protein sequences were obtained from the available databases by BLAST searching (Altschul et al., 1997). Multiple alignments were performed using MUSCLE (Edgar, 2004) and well-aligned sites were chosen using G-Blocks (Castresana, 2000) with settings to minimize the stringency of selection. Maximum likelihood (ML) phylogenetic reconstructions incorporating 500 bootstrap replicates were performed in PhyML (Guindon et al., 2010) using the LG substitution model (Le and Gascuel, 2008).

### Gene sequence annotation

To identify genes potentially encoding VPEs in *Amborella, Arabidopsis* VPE sequences retrieved from the *Arabidopsis* Information Resource (TAIR) (Swarbreck, 2008; http://www.arabidopsis.org) were blasted against the *Amborella* EVM 27 Predicted Protein database (http://www.amborella.org/). The annotations of the scaffolds containing the predicted VPE genes (NCBI accession # NW_006499912.1 and NW_006497648) were then manually checked and eventually re-annotated using the Lasergene Genomics Suite (http://www.dnastar.com/t-products-dnastar-lasergene-genomics.aspx; DNASTAR Inc., USA). The newly annotated VPE sequences were submitted to NCBI under the accessions n° BK009356 and BK009357. The previous annotated cds # XM_006853855.2 was not modified.

### Gene sequence polymorphism

Next-generation resequencing data from the *Amborella* genome (*Amborella* Genome Project, 2013) were used to characterize SNP polymorphisms corresponding to the VPE sequences characterized in the present work. The genomic information has been generated for 12 individuals (named according to their location: Tonine, Ponandou, Pwicate, Tchamba, Ba, Aoupinié, Boregaou, Amieu, Dogny, Mé Ori, Mé Fomechawa, and Nakada) covering a wide range of *Amborella's* extant geographical distribution and natural genetic diversity (Poncet et al., 2013).

All the SNPs identified were defined as informative with a minor allele frequency (MAF) >0.08 and a missingness rate < 0.42 by SNP. Biallelic exonic SNPs were annotated as synonymous or non-synonymous according to their reference/alternate allele nucleotides and to their position in the updated annotated coding sequences.

For each of the 12 individuals, the average MAF values were first computed across all SNPs for each of the three genes. Then, for each of the four genetic clusters inferred by a previous microsatellite (simple sequence repeats, SSR) analysis (Poncet et al., 2013), namely North (Tonine, Ponandou, Pwicate, Tchamba), Center (Ba, Aoupinié, Boregaou, Amieu, Dogny), Me (Mé Ori, Mé Fomechawa), and Nak (Nakada), we calculated for each gene (i) the mean proportion of polymorphic SNPs and (ii) the mean proportion of private alleles. Computations were performed on intronic and exonic SNP datasets independently by discarding missing data.

To assess any impact of natural selective pressures on VPE sequences, we examined the SNP patterns in comparison with 10 neutrally-behaving SSRs. Polymorphisms under neutral evolution co-vary with divergence between populations regardless of the mutation rate (Hartl and Clark, 2007). Marks of selection on SNP markers would be detected as polymorphism deviating from the expected neutral background polymorphism (influenced by forces such as divergence as well as other demographic events).

## Results

### Shotgun proteomic analyses

**Figure 1:**
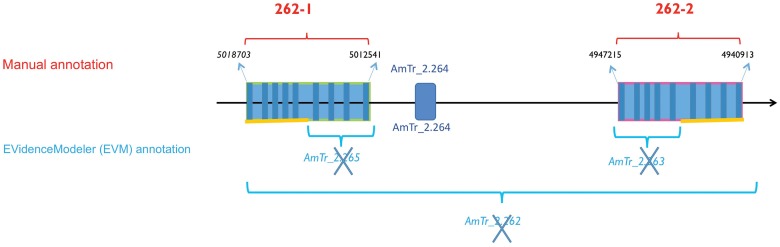
**Gene duplication model for the β-type *Amborella* VPEs on scaffold 2**. The two duplicated genes (*AmTr_2.262-1* and *AmTr_2.262-2*) show 84.5 % similarity at the nucleotide sequence level and 98.8 % at the protein level. **Top:** The manual annotation of the two closely related VPE genes, *AmTr_2.262-1* and *AmTr_2.262-2*, referred to 262-1 and 262-2, respectively and the gene encoding the pectinesterase/pectinesterase (*AmTr_ 2.264*). **Bottom:** In the framework of the *Amborella* Genome Project (2013) the EVidenceModeler (EVM) program (http://evidencemodeler.sourceforge.net) automatically annotated VPE genes within the region, leading to wrongly annotated, supernumerary and truncated genes.

A shotgun proteomic analysis revealed 415 proteins from the isolated *Amborella* embryos (Villegente et al., unpublished results). In particular, this analysis confirmed the presence of three 11S globulin forms in the *Amborella* embryos, of which two contained canonical N-G VPE cleavage sites, while the third contained a variant cleavage site (N-V-I) (data not shown). This shotgun proteomic analysis also revealed the presence of two specific peptides (GIIINHPQGEDVYAGVPK and HQADVCHAYQLLLK) (Supplemental Table S1) matching with the amino acid sequences of *Amborella* VPE proteins encoded by sequences on the scaffold AmTr_v1.0_scaffold 00002, labeled 27.model.AmTr_v1.0_scaffold00002.262 and evm_27.model.AmTr_v1.0_scaffold00002.263 (Figure 1).

### The *Amborella* VPE family

To identify other genes potentially encoding VPEs in *Amborella*, we blasted the *Arabidopsis* VPE sequences retrieved from TAIR (Swarbreck, 2008; http://www.arabidopsis.org) against the *Amborella* EVM 27 Predicted Protein database (http://www.amborella.org/). Two additional *loci* were thus revealed, namely evm_27.model.AmTr_v1.0_scaffold00036.100 (designated below as *AmTr_36.100*) and evm_27.model.AmTr_v1.0_scaffold00002.265 (*AmTr_2.265*). To confirm the automatic annotation (http://www.amborella.org/ and http://amborella.uga.edu/) a manual annotation was performed on the retrieved scaffold sequences. The automatic annotation process of the scaffold AmTr_v1.0_scaffold 00002 was actually wrong leading to truncated genes (Figure 1). Two full-length genes duplicated in tandem on the same scaffold (AmTr_v1.0_scaffold00002) were in fact identified and designated as *AmTr_2.262-1* and *AmTr_2.262-2*; (Figure 1). They are each composed of nine predicted exons and eight predicted introns like *AmTr_36.100*. This structure is shared by almost all VPE genes from the available sequence databases representing green algae, bryophytes, lycophytes, gymnosperms, monocots, and eudicots (data not shown). The two duplicated genes (*AmTr_2.262-1* and *AmTr_2.262-2)* show 84.5% similarity at the nucleotide sequence level and 98.8% at the protein level (Supplemental Figure S1). The amino acid sequences encoded by all three predicted *Amborella* VPE genes exhibit the two conserved amino acid residues (H and C), which are also present in the active sites of all known active VPEs (Chen et al., 1998, 2004) (Supplemental Figure S1). BLAST searching of the *Amborella* RNASeq Trinity Assembly using the online *Amborella* Genome Database (http://www.amborella.org) identified expressed sequences corresponding to *AmTr_2.262-2* (gnl|Ambo_Trinity|comp32807_c0_seq1 and >gnl|Ambo_Trinity|comp23371_c0_seq1) and *AmTr_36.100* (gnl|Ambo_Trinity|comp847_c0_seq1 and gnl|Ambo_Trinity|comp777_c0_seq1), though not to *AmTr_2.262-1*.

We conclude that the *Amborella* VPE family is composed of three genes: *AmTr_2.262-1, Am_Tr2.262-2*, and *AmTr_36.100* (Figure 1; Supplemental Figure S1), of which the former two are very closely related. *AmTr_2.262-2* and *AmTr_36.100* are both transcribed, while the transcription of *AmTr_2.262-1* remains to be demonstrated. It should be noted among others that no seed tissues were used to obtain transcribed sequences in the available *Amborella* transcriptome databases (see http://ancangio.uga.edu/content/amborella-trichopoda), and so it is possible that *AmTr_2.262-1* might be expressed, but shows an entirely seed-specific expression profile. Both of the peptide signatures identified using the proteomics approach described in the present work (GIIINHPQGEDVYAGVPK and HQADVCHAYQLLLK) show 100% identity to fragments of the predicted genes *AmTr_2.262-1* and *AmTr_2.262-2* (of which the latter is certainly transcribed and the former could be transcribed in other organs and tissues that were not surveyed in the available data, including fruits and seeds). No peptide signatures from *AmTr_36.100*, which is most presumably transcribed in non-seed tissues, were detected in seeds in the proteomics approach described in the present work.

Most of our current knowledge of the genes encoding VPEs and their biological functions comes from molecular and genetic studies of the model plant *Arabidopsis*. In this species, four VPE homologs have been described: α-VPE and γ-VPE, which are specific to vegetative organs, β-VPE, which is specific to seeds, and δ-VPE, which is involved in seed coat formation (Nakaune et al., 2005; Hatsugai et al., 2015) and in the processing and degradation of various proteins in senescent *Arabidopsis* tissues (Rojo et al., 2003).

To reveal potential biological functions of the *Amborella* VPEs, the amino acid sequences of the three *Amborella* VPEs were blasted at TAIR against the *Arabidopsis* genome. An analysis of the scores obtained from this comparison disclosed that the *AmTr_36.100* gene would encode a γ-type VPE (Supplemental Figure S2), while the *AmTr_2.262-1* and *AmTr_2.262-2* genes would encode β-type VPEs (Supplemental Figure S2).

### Phylogenetic analyses

To gain further insight in plant VPEs and benefiting from the present *Amborella* sequences, the amino acid sequences of VPEs identified by BLAST from a wide range of embryophytes (land plants) taxa were used for phylogenetic reconstruction. These sequences were first blasted at TAIR against the *Arabidopsis* genome. For all VPEs presently considered, the best hits corresponded to *Arabidopsis* VPEs, testifying of high amino acid sequence conservation among plant VPEs (data not shown).

**Figure 2:**
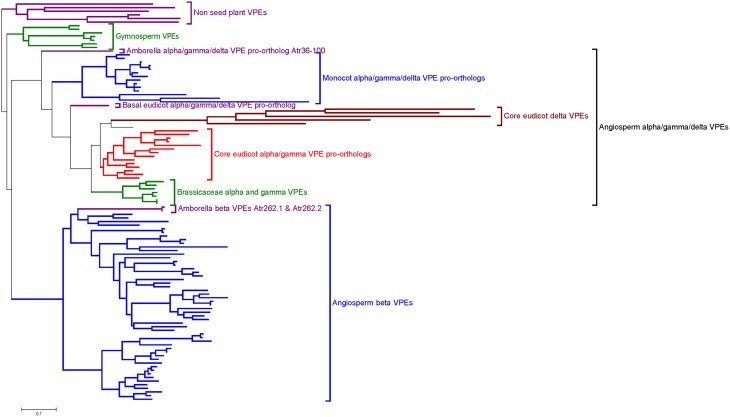
**Summary of an ML phylogeny of plant VPE proteins, rooted between seed and non-seed plant VPEs, showing *Amborella* proteins in the most basal positions of two well-supported clades of angiosperm α/γ/δ-VPEs and β-VPEs, respectively**. The figure only shows a simplified tree (see Materials and Methods). This phylogeny is given in full in Supplemental Figure S3. Amino acid sequences used to construct the tree are listed in Supplemental Figure S2.

The phylogenetic reconstruction of embryophyte VPE proteins (Figure 2; Supplemental Figure S3) shows two sister clades of VPEs from angiosperms, of which one clade contains the α-, γ-, and δ-VPE proteins from *Arabidopsis* (“Angiosperm α/γ/δ-VPE clade”), while the other contains the β-VPE protein from *Arabidopsis* (“Angiosperm β-VPE clade”). The presence of *Amborella* VPEs within each of these two clades is very well supported (100% bootstrap support in each case). *Amborella* proteins occupy basal positions in both the angiosperm α/γ/δ- and β-VPE clades, albeit with modest bootstrap support. Gymnosperm VPEs and non-seed plant VPEs group in two further clades, externally to the combined clade of angiosperm α/γ/δ- and β-VPEs.

The above topology clearly indicates that the duplication event that generated the respective ancestors of the angiosperm α/γ/δ- and β-VPE clades occurred before the MRCA of the extant angiosperms. The absence of clearly distinguished α/γ/δ- and β-VPEs in gymnosperms, and the position of the gymnosperm VPE clade as sister to a clade containing all angiosperm VPEs (albeit with modest bootstrap support), suggests that the duplication that separated the angiosperm α/γ/δ- and β-VPE lineages occurred along the angiosperm stem lineage, after its separation from that of the living gymnosperms.

*Arabidopsis* δ-VPE occurs in a well-supported sub-clade of the angiosperm α/γ/δ-VPE clade, together with genes from widely diverged core eudicots including *Medicago, Vitis*, and *Populus*. By contrast, all α/γ/δ-clade VPEs from *Amborella*, monocots and basal eudicots (such as *Papaver*) group externally to the point of divergence of the δ-VPE sub-clade (Figure 2; Supplemental Figure S3). It thus appears that the δ-VPE lineage arose in a gene duplication event in a common ancestor of all, or a major part of, the living core eudicots. Branch lengths within the δ-VPE sub-clade are very long compared to those in the remainder of the angiosperm α/γ/δ-VPE clade, suggesting either strong positive or relaxed selection pressure to have operated on the δ-VPE lineage since its separation from that of the remaining α/γ/δ-VPE lineage (Guindon et al., 2004).

The *Arabidopsis* α- and γ-VPEs (Ath_gi15225226 and Ath_gi15233996) group with 100% bootstrap support in a small clade containing sequences only from closely related Brassicaceae, including *Arabidopsis lyrata* and *Eutrema salsugineum* (Figure 2; Supplemental Figure S3). This topology strongly suggests that the duplication that generated the α- and γ-VPE lineages occurred recently, within Brassicaceae. This conclusion accords well with the common expression pattern of *Arabidopsis* α- and γ-VPEs in vegetative organs.

The phylogeny in Figure 2 and Supplemental Figure S3 shows that multiple, closely related VPE proteins are present in numerous taxa, including monocots, *Amborella*, eudicots, and gymnosperms. Thus, relatively recent gene duplications, such as that which generated the α- and γ-VPE lineages in *Arabidopsis*, appear to have occurred frequently within the VPE family in diverse plant groups.

### Gene sequence polymorphism and *Amborella* population analyses

**Figure 3:**
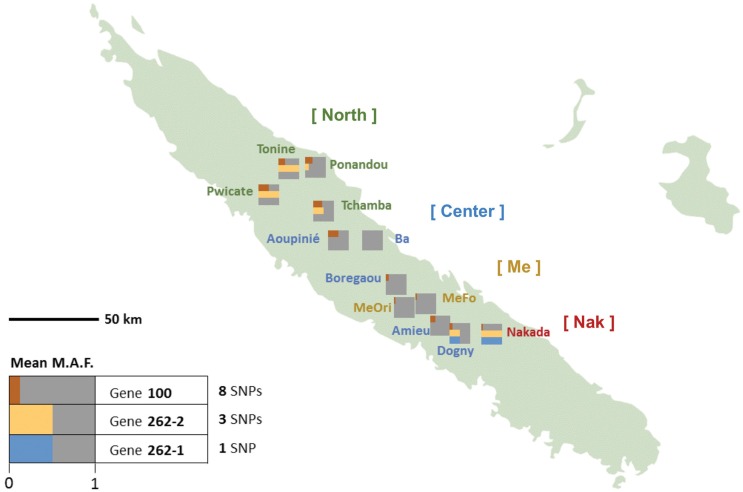
**Frequencies distribution of *Amborella* VPE exonic SNPs in New Caledonia**. For each genotype, the exonic mean minor allele frequencies (MAF) are represented by colored bar proportional to their mean values in each of the three VPE genes: *AmTr_36.100* (“100”, red), *AmTr_2.262-1* (“262-1”, blue) and *AmTr_2.262-2* (“262-2”, yellow). The corresponding population names are given together with their assignation to the four genetic clusters as inferred by SSR analysis (Poncet et al., 2013): “North” (Tonine, Ponandou, Pwicate, Tchamba) in green, “Center” (Ba, Aoupinié, Boregaou, Amieu, Dogny) in blue, “Me” (Mé Ori, Mé Fomechawa) in yellow, and “Nak” (Nakada) in red.

Nucleotide sequence polymorphism was characterized at both the intron and exon levels for the *Amborella* VPE genes described in the present work, from 12 individuals that were considered to be representative of *Amborella's* extant geographical distribution and genetic diversity (Poncet et al., 2013) (Figure 3; Supplemental Figure S4).

### Impact of exonic SNPs on protein structure

SNPs recorded across the studied individuals revealed a similar number of intronic and exonic polymorphisms for the three genes, with one to eight exonic SNPs and 22 to 34 intronic SNPs per gene. Among the 12 SNPs located in the protein-coding regions of the three genes, seven SNPs were synonymous and five were non-synonymous, though none was found located in the codons encoding catalytic residues (Supplemental Figure S4).

### Minor allele frequency (MAF) and genetic diversity within *Amborella* distribution

**Figure 4:**
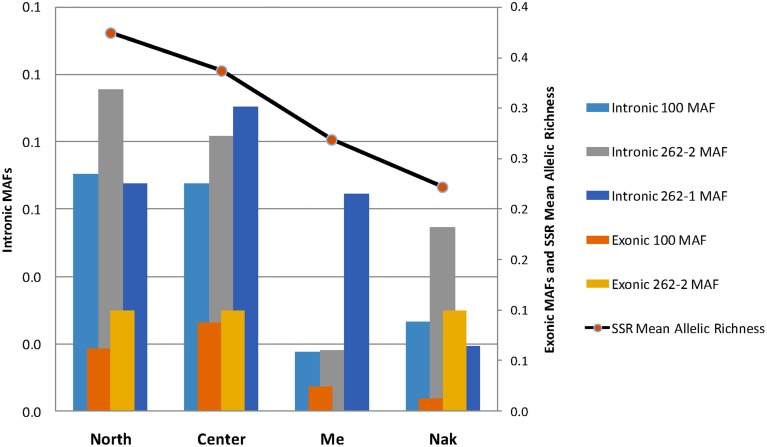
**Mean proportion of exonic and intronic polymorphic SNPs in the four genetic groups (North, Center, Me, and Nak; see Figure 3)**. The mean minor allele frequencies (MAF) are compared to the mean SSR allelic richness obtained on the same groups by Poncet et al. (2013).

We examined the MAF statistics of *Amborella* VPE genes within the previously described genetic groups North (Tonine, Ponandou, Pwicate, Tchamba), Center (Ba, Aoupinié, Boregaou, Amieu, Dogny), Me (Mé Ori, Mé Fomechawa), and Nak (Nakada). For each of the individuals and genetic groups studied, average MAFs were computed for each *Amborella* VPE gene (Figures 3, 4; Supplemental Figure S4). For exonic SNPs, higher frequencies were observed for the northern (Ponandou, Pwicate, Tonine, and Tchamba) individuals for genes *AmTr_36.100* and *AmTr_2.262-2*, and for Nakada individual for genes *AmTr_2.262-1* and *AmTr_2.262-2* (Figure 3). This trend was comparable to the diversity distribution observed with SSR microsatellites (Poncet et al., 2013) with individuals from the north exhibiting higher diversity and clustering in a same group. A similar distribution was also observed for intronic SNPs, with the northern group displaying a high percentage of polymorphic SNPs (71, 68, and 95% for genes *AmTr_36.100, AmTr_2.262-1*, and *AmTr_2.262-2*, respectively), the highest percentage of private SNPs (18, 6, and 18% for genes *AmTr_36.100, AmTr_2.262-1, and AmTr_2.262-2*, respectively) and a mean MAF of 0.43 across all three genes, the highest among all groups (Supplemental Figure S4). In particular, changes in the mean proportion of exonic and intronic polymorphic SNPs across the four genetic groups follow a parallel progression with the mean SSR allelic richness (Figure 4). Levels of naturally occurring genomic variations in the VPE sequences thus appeared to co-vary with divergence and demographic history between populations and in particular with neutrally behaving polymorphisms (SSRs).

## Discussion

### The *Amborella* VPE family

The present work shows that three VPE genes are present in the sister to all other living angiosperms, *Amborella trichopoda.* Two of these, *AmTr_2.262-1* and *AmTr_2.262-2*, encode closely related β-VPEs, which are orthologs of the single β-VPE found in *Arabidopsis.* Our proteomics work shows that at least one of these two genes is expressed in seeds, thus generating the peptide fragments GIIINHPQGEDVYAGVPK and HQADVCHAYQLLLK, which we detected. Transcriptomics data available through both the *Amborella* Genome Database (http://www.amborella.org) and Ancestral Angiosperm Genome Project website (http://ancangio.uga.edu) indicate that *AmTr_2.262-2* is expressed in non-seed tissues, though this gene may additionally be expressed in seeds, as no seed tissues were used to obtain these transcriptomics data. We conclude that *AmTr_2.262-1* is either not expressed, or is specifically expressed in seeds. The third VPE gene present in *Amborella, AmTr_36.100*, appears from phylogenetic studies to be a pro-ortholog *of the* α-, γ-, and δ-VPEs in *Arabidopsis*. From transcriptomics analyses, *AmTr_36.100* is transcribed in non-seed tissues, while the proteomics analysis presented here failed to show any peptide signatures derived from this gene in a seed-protein extract. We therefore conclude that *AmTr_36.100* is the *Amborella* pro-ortholog of the α-γ- and δ-VPEs from *Arabidopsis* and shows an entirely non-seed expression profile. The analysis of the *Amborella* VPE family performed in the present work has allowed us to draw solid conclusions (see following section) on the state of the VPE family in the MRCA of the extant angiosperms which no previous study has been able to make.

### A partial reconstruction of the evolution of the VPE family in angiosperms

The *Arabidopsis* VPE family, whose expression patterns and functions are the best characterized of any plant species, consists of four genes encoding α-, β-, γ-, and δ-VPEs. Previous phylogenetic studies have succeeded in identifying several major clades of plant VPEs (Nakaune et al., 2005; Christoff et al., 2014). However, these studies did not clearly elucidate the deep evolutionary relationships between the major clades of VPEs identified, or the origins through gene duplication of the VPE lineages present in *Arabidopsis* or other established plant models.

**Figure 5:**
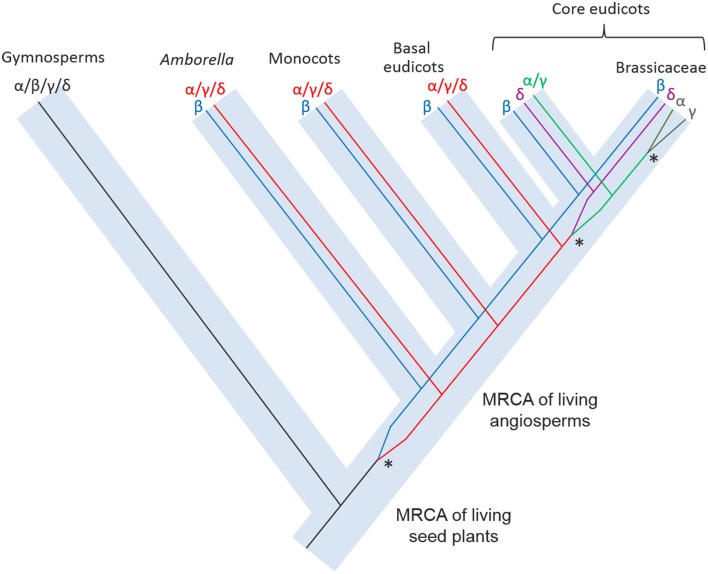
**A schematic reconstruction of the evolution of the VPE family in plants showing the origins of the duplication events that occurred at various evolutionary stages**. Asterisks indicate the gene duplication events that produced the four VPEs in present-day *Arabidopsis*.

The phylogenetic reconstructions presented here (Figure 2; Supplemental Figure S3), incorporating novel sequences from *Amborella* and a wide range of land plants, indicate the four VPE genes in *Arabidopsis* to have been generated through three duplication events that occurred at quite distinct evolutionary stages. The conclusions of this analysis are summarized in Figure 5. In non-seed plants, including bryophytes such as *Physcomitrella* and non-seed, vascular plants such as *Selaginella*, several VPEs are found, though these group together in a clade that is positioned externally to all seed plant VPEs. Thus, the α-, β-, γ-, and δ-VPEs of *Arabidopsis* share no direct one-to-one relationships of orthology with VPEs in non-seed plants. Similarly, multiple gymnosperm VPEs occur together in a clade that groups, though with modest bootstrap support, in a sister position to a clade containing all angiosperm VPEs. Accordingly, the α-, β-, γ-, and δ-VPEs of *Arabidopsis* appear to share no direct one-to-one relationships of orthology with VPEs from gymnosperms.

By contrast, in *Amborella*, the most basally diverging angiosperm, two VPE lineages are present with distinct relationships of orthology to the VPEs from *Arabidopsis*. Accordingly, proteins encoded by *AmTr_2.262-1* and *AmTr_2.262-2* are both orthologous to *Arabidopsis* β–VPE and, from our proteomics data, appear to have conserved a similar expression profile in seeds to that of *Arabidopsis* β–VPE. This result implies that angiosperm β–VPEs have conserved their seed-specific expression pattern since the MRCA of the living flowering plants, at least in the lineages leading to *Amborella* and to *Arabidopsis*. The protein encoded by *AmTr_36.100* is the putative *Amborella* pro-ortholog of all three other *Arabidopsis* VPEs (the α-, γ-, and δ-VPEs) and appears to have conserved a non-seed specific expression profile since the MRCA of living angiosperms.

The role of *Arabidopsis* β-VPE is to process the major protein reserves so these are correctly assembled within PSVs during seed maturation on the mother plant (Shimada et al., 2003). The need for such a role logically arises only in seed plants, and in this regard, the absence of a direct ortholog of β-VPEs in non-seed plants, such as bryophytes and lycophytes, is not incongruent. However, the duplication event that generated the β-VPE lineage appears to be specific to angiosperms, such that the remaining seed plants, the gymnosperms, also lack direct orthologs of the angiosperm β-VPE lineage. This apparent incongruity might be explained in at least two distinct ways, which relate to the functions and expression of VPE orthologs. Firstly, gymnosperms, including *Pinus* and *Picea*, appear like angiosperms to possess multiple VPE isoforms, and these are orthologous as a group to all angiosperm VPEs, including the β-VPEs. Therefore, a careful analysis of VPE expression patterns and/or protein accumulation in gymnosperms might show that some gymnosperm VPEs, like angiosperm β-VPEs, play a specific role in the mobilization of seed protein reserves. A second explanation for the differences between the VPE family in angiosperms and gymnosperms might relate to a possibly lesser functional specificity of VPEs in gymnosperms compared to angiosperms, such that the same VPEs in gymnosperms might be responsible both for the mobilization of seed protein reserves and for the processing of proteins in vegetative tissues.

The separate origins of the α-, γ-, and δ-VPE lineages, as these occur in *Arabidopsis*, have been considerably elucidated in our phylogenetic analyses (Figure 2; Supplemental Figure S3). Accordingly, the δ-VPE lineage appears to have arisen by a gene duplication in a common ancestor of widely diverged core eudicot taxa including *Arabidopsis, Medicago, Populus*, and *Vitis*. The δ-VPE lineage is thus not present as a separate lineage in basal eudicots, monocots or *Amborella*, but is represented in these taxa by single or multiple pro-orthologs of all core-eudicot α-, γ-, and δ-VPEs. Interestingly, the very long branches within the core eudicot δ-VPE clade indicate this gene lineage to have evolved very rapidly, which may have been due to intense positive selection pressure, or to a very relaxed selection, leading to rapid, neutral evolution. Finally, the α- and γ-VPEs of *Arabidopsis* show direct one-to-one relationships of orthology only to VPEs from species of closely related Brassicaceae. These two genes therefore must have arisen through a gene duplication event within Brassicaceae.

The rapidity of evolution of the δ-VPE lineage compared to the remaining α/γ-lineage within the core eudicots suggests that the α- and γ-VPEs of *Arabidopsis* may have largely conserved the ancestral functions of the α/γ/δ lineage, while the δ-VPE lineage may have either acquired a novel function, or become specialized through sub-functionalization. Further experiments (e.g., RNASeq, RT-PCR) are needed to carefully analyze the expression of the three *Amborella* genes in seeds and vegetative tissues and different developmental stages. For example, it would be interesting to characterize the expression patterns of seed VPEs at the stage at which unprocessed pro-globulins forms accumulate in maturating seeds. Accordingly, it would be worth performing a thorough expression analysis of all three *Amborella* VPE genes in both vegetative and seed tissues. In particular, this would allow us to determine whether one of these genes is expressed in the seed coat, as is δ-VPE in *Arabidopsis*.

### Gene sequence polymorphism along VPE genes in natural populations

The intra-specific genetic diversity of *Amborella* for the three VPE genes was explored at both the exon and intron levels using 12 individuals that are considered representative of *Amborella's* extant geographical distribution and genetic diversity in New Caledonia (Poncet et al., 2013). While SNPs in introns were most frequent among all SNPs located in the protein-coding regions of the three genes, most of them were synonymous or led to changes for amino acid with similar polarities, and never in the enzyme active sites, suggesting low impact on the protein function. Moreover, the polymorphism pattern across individuals was totally congruent with the genetic structure previously observed with neutrally behaving markers, SSRs (Poncet et al., 2012, 2013) or with SNPs distributed throughout the genome (*Amborella* Genome Project, 2013) on the same populations (Figures 3, 4; Supplemental Figure S4).

If there were selective pressure acting on SNP polymorphisms, these three genes would not have co-varied with neutrally-behaving markers (SSRs and SNPs), and population divergence. Because no obvious signature of natural selection was detected either in exons or introns, the observed patterns of polymorphism were probably shaped by neutral demographic forces only, i.e., genetic drift and migration.

The present geographical distribution of *Amborella* in New Caledonia has been suggested to be a signature of the impact of ancient climatic changes, and notably of the severe restriction of favorable environments during the last glacial maximum (LGM) (*ca.* 22,000 years BP) and Holocene (*ca.* 12,000 years BP) when *Amborella* experienced a dramatic reduction (about 96%) in suitable area (Poncet et al., 2013). The survival and expansion of at least two lineages from putative refugia might have generated major diversity groups. The present data on VPE polymorphisms across the species provides an additional signature of the biogeographical history of *Amborella*.

### Conflict of interest statement

The authors declare that the research was conducted in the absence of any commercial or financial relationships that could be construed as a potential conflict of interest.

## Abbreviations

1D: one dimensional
ACN: acetonitrile
AGP: *Amborella* Genome Project
CDS: coding sequence
CHAPS: 3-[(3-cholamidopropyl) dimethylammonio]-1-propanesulfonate
DTT: dithiothreitol
ER: endoplasmic reticulum
HPLC: high-performance liquid chromatography
i.d.: internal diameter
LC-MS/MS: liquid chromatography coupled to tandem mass spectrometry
MAF: minor allele frequency
ML: maximum likelihood
MRCA: most recent common ancestor
Mya: millions years ago
PCD: programmed cell death
PSV: protein storage vacuole
SDS-PAGE: sodium dodecylsulfate polyacrylamide gel electrophoresis
SNP: single nucleotide polymorphism
SSR: simple sequence repeats
TAIR: The *Arabidopsis* Information Resource
TFA: trifluoroacetic acid
VPE: vacuolar processing enzyme.
