# Supplementary material for: The Amborella vacuolar processing enzyme family
Source: Front Plant Sci. 2015 Aug 21;6:618. doi: 10.3389/fpls.2015.00618 (PMC4544213; doi:10.3389/fpls.2015.00618)
Supplement: Supplementary file 1 [file Table1.PDF]

Supplemental Table S1: LC-MS/MS data for *Amborella* VPEs

Summary of identified VPEs

Description	log(E value)	Coverage	MW	Spectra	Uniques	PAI	Redundancy
evm_27.model.AmTr_v1.0_scaffold00002.262	-9.66254	7	50.3	2	2	0.125	2
evm_27.model.AmTr_v1.0_scaffold00002.263	-9.66254	11	30.1	2	2	0.25	2

Summary of identified VPE peptides

Description	Rt	Sequence	Modifications	E-value	Charge	MH+ Obs	MH+ theo	Delta-MH+	Delta-ppm
evm_27.model.AmTr_v1.0_scaffold00002.262	26.2	GIIINHPQGEDVYAGVPK		0.0025	3	1907.0104	1907.0026	0.0078	4.090188
evm_27.model.AmTr_v1.0_scaffold00002.262	28.82	HQADVCHAYQLLLK	C6: +57.02146	8.7E-8	3	1695.864	1695.864	1.0E-4	0.05896699

log(E value) E-value of the protein, expressed in log
Coverage Coverage of the protein expressed in %.
MW Molecular weight, kDa.
Spectra Number of spectra allowing the identification of the protein
Uniques Number of unique peptides allowing identification of the protein
PAI Protein Abundance Index
Redundancy Number of proteins identified with the same pool of spectra
Rt Retention time of the peptide
Sequence Identified peptide sequence
Modifications Identified modifications of the peptide
MH+ Obs Observed monoisotopic mass for the identified peptide with the addition of one proton (MH+)
MH+ theo Theoretical monoisotopic mass for the identified peptide with the addition of one proton (MH+)
Delta-MH+ Difference in mass between MH+ Obs and MH+ theo (Da)
Delta-ppm Difference in mass between MH+ Obs and MH+ theo (ppm)
