# Supplementary material for: The Amborella vacuolar processing enzyme family
Source: Front Plant Sci. 2015 Aug 21;6:618. doi: 10.3389/fpls.2015.00618 (PMC4544213; doi:10.3389/fpls.2015.00618)
Supplement: Supplementary file 2 [file Image1.PDF]

```
           1                                                50
   262-1   MATSDRRSSL FYFLLLSLLL FRQRSFGVRV HEWVDSVIRM PSQKEGGEGV
   262-2   MATSGLRRSL FYFLLLSLLL FRQRSFGVRV NEWVDSVIRM PSQKEGGEGV
  36.100        MAFSG KSVLFLAVFM AFSGVYG-RY STWSD-FLRM PQTEDS---V
Consensus  mats..r.Sl fyfLlLsll$ frqrs%GvRv .eWvDsviRM Psqk#ggegV

           51                                              100
   262-1   GTRWAVLVAG SSGFGNYRHQ ADVCHAYQLL LKGGLKEENI VVFMHDDIAY
   262-2   GTRWAVLVAG SSGFGNYRHQ ADVCHAYQLL LKGGLKEENI VVFMHDDIAY
  36.100   GTRWAVLVAG SSGYGNYRHQ ADICHAYQTM IRGGLKEKNI VVFMYDDIAY
Consensus  GTRWAVLVAG SSG%GNYRHQ AD!CHAYQl$ lkGGLKEeNI VVFMhDDIAY

           101                                             150
   262-1   NEFNPKKGII INHPQGEDVY AGVPKDYTGK QVHTKNLYAV LLGNNSAVTG
   262-2   NEFNPKKGII INHPQGEDVY AGVPKDYTGK QVHTKNLYAV LLGNNSAVTG
  36.100   NEENPRPGVI INRPHGEDVY AGVPKDYVGD DVNVDNLFAV ILGNKSALTG
Consensus  NEfNPkkG!I INhPqGEDVY AGVPKDYtGk #VhtkNL%AV lLGNnSAvTG

           151                                             200
   262-1   GSGKVINSKA EDRIFIYYSD HGGPGVLGMP NMPFLYANDL MEVLKKKHKS
   262-2   GSGKVINSKA EDRIFIYYSD HGGPGVLGMP NMPFLYANDL MEVLKKKHKS
  36.100   GSGKVVDSGP DDHIFIFYSD HGGAGVLGMP TYPYLYADDL VNVLKKKHVS
Consensus  GSGKV!#Ska #DrIFI%YSD HGGpGVLGMP nmP%LYA#DL m#VLKKKHkS

           201                                             250
   262-1   KGYKEMVIYV EACESGSIFE GLMTEDLNIY VTTASNAQES SWGTYCPGMD
   262-2   KGYKEMVIYV EACESGSIFE GLMTEDLNIY VTTASNAQES SWGTYCPGMD
  36.100   GTYKSLVFYL EACESGSIFE GLLPEGLNIY ATTASNAVES SWGTYCPDDS
Consensus  kgYKe$ViYv EACESGSIFE GL$tEdLNIY vTTASNAqES SWGTYCPgmd

           251                                             300
   262-1   PPPPPEFMTC LGDLYSVAWM EDSETHNLKE ETIQKQYVRV KSRTSNYNTY
   262-2   PPPPPEFMTC LGDLYSVAWM EDSETHNLKE ETIQKQYVRV KSRTSNYNTY
  36.100   PDFPQEYDTC LGDLYSVSWM EDSDIHNLQF ETLKQQYELV KMRTSNFETY
Consensus  PppPpE%mTC LGDLYSVaWM EDS#tHNLke ETiqkQYvrV KsRTSN%#TY

           301                                             350
   262-1   TAGSHVMEYG DKSIKSERLY LYQGFDPANA NLSDNSLPLQ PNRMDVVNQR
   262-2   TAGSHVMEYG DKSIKSERLY LYQGFDPANA NLSDNSLPLQ PNRMDVVNQR
  36.100   MFGSHVMQYG DSGLGKEQLV LYMGSNPAND NSTFISRNEL PSFSKAVNQR
Consensus  taGSHVM#YG DksiksErLy LYqGf#PANa NlsdnSlplq PnrmdvVNQR

           351                                             400
   262-1   DADLLFLWQR YKRSTEGSEE KVAFRNEMTE KMAHREHLDK SVDLIGRLLF
   262-2   DADLLFLWQR YERSTEGSEE KVAIRNEMTE KMAHREHLDK SVDLIGRLLF
  36.100   DADLVYFWNK YRKSPVGSIK KRNAQKELFD VMAHRLHLDN SIELIGKLLF
Consensus  DADLl%lW#r Y.rSteGSee Kva.rnE$t# kMAHReHLDk S!#LIGrLLF

           401                                             450
   262-1   GWDKGSNVLG AKRPSGKALV DDWSCLKSMV RAFEEKCGPL TQYGMKHMRA
   262-2   GWDKGSNVLG AKRPSGKALV DDWSCLKSMV RAFEEKCGPL TQYGMKHMRA
  36.100   GSEKGPEILK TVRTTGLPLV DDWDCLKAMV RTFETKCG
Consensus  Gw#KGs#!Lg akRpsGkaLV DDWsCLKsMV RaFEeKCGpl tqygmkhmra

           451                                     491
   262-1   FANICNEGIS LEVMSKACEE VCGRTYKHGI LNVASHHGFS G
   262-2   FANICNEGIS LEVMSKACEE VCGRTYKHGI LNVASHHGFS G
  36.100
Consensus  fanicnegis levmskacee vcgrtykhgi lnvashhgfs g
```

Supplemental Figure S1: Alignment of the amino acid sequences of the three VPEs present in the *Amborella* genome. The proteins predicted by the *Amborella* genes *AmTr_261-1*, *AmTr_262-2* and *AmTr_36.100* are referred to as 261-1, 262-2 and 36.100, respectively. Protein sequence alignments were performed at http://multalin.toulouse.inra.fr/multalin/multalin.html (Corpet, 1988). Signal peptide predictions (http://www.cbs.dtu.dk/services/TargetP/; Emanuelsson et al., 2007) are shown in pink (the scores for prediction as secreted proteins were 0.915, 0.922 and 0.85 for 261-1, 262-2 and 36.100, respectively). The VPE catalytic residues are shown in green. The experimental peptides obtained for 261-1 and/or 262-2 by shotgun MS/MS analysis of embryo *Amborella* seeds (GIIINHPQGEDVYAGVPK and HQADVCHAYQLLLK; Supplemental Table S1) are shown in yellow.

Corpet, F. (1988) Multiple sequence alignment with hierarchical clustering. *Nucleic Acids Res.* **16**, 10881-10890

Emanuelsson, O., Brunak, S., von Heijne, G., and Nielsen, H. (2007) Locating proteins in the cell using TargetP, SignalP, and related tools. *Nature Protocols* **2**, 953-971
