# Supplementary material for: The Amborella vacuolar processing enzyme family
Source: Front Plant Sci. 2015 Aug 21;6:618. doi: 10.3389/fpls.2015.00618 (PMC4544213; doi:10.3389/fpls.2015.00618)
Supplement: Supplementary file 3 [file Image2.PDF]

Supplemental Figure S2: Amino acid sequences of plant VPEs

A) Correspondence between *Amborella* and *Arabidopsis* VPE proteins. Amino acid sequences of *Amborella* Vacuolar Processing Enzyme (VPE) proteins, encoded by *AmTr_262-1*, *AmTr_262-2* and *AmTr_36.100*, were blasted at https://www.arabidopsis.org/cgi-bin/Blast/TAIRblast.pl and the best *Arabidopsis* hits selected (TAIR BLAST 2.2.8, last access 23 April 2015).

Amborella* Gene *AmTr_262-1

MATSDRRSSLFYFLLLSLLLFRQRSFGVRVHEWVDSVIRMPSQKEGGEGVGTRWAVLVAGSSGFGNYRHQADVCHAYQLLLKGGLKEENIVVFMHDDIAYNEFNPKK
GIIINHPQGEDVYAGVPKDYTGKQVHTKNLYAVLLGNNSAVTGGSGKVINSKAEDRIFIYYSDHGGPGVLGMPNMPFLYANDLMEVLKKKHKSKGYKEMVIYVEACE
SGSIFEGLMTEDLNIYVTTASNAQESSWGTYCPGMDPPPPPEFMTCLGDLYSVAWMEDSETHNLKEETIQKQYVRVKSRTSNYNTYTAGSHVMEYGDKSIKSERLYL
YQGFDPANANLSDNSLPLQPNRMDVVNQRDADLLFLWQRYKRSTEGSEEKVAFRNEMTEKMAHREHLDKSVDLIGRLLFGWDKGSNVLGAKRPSGKALVDDWSCLKS
MVRAFEEKCGPLTQYGMKHMRAFANICNEGISLEVMSKACEEVCGRTYKHGILNVASHHGFSG

Arabidopsis *sequences producing significant alignments*:		Score (bits)	E Value
AT1G62710.1	Symbols: BETA-VPE, BETAVPE	626	e-180
AT4G32940.1	Symbols: GAMMA-VPE, GAMMAVPE	541	e-154
AT2G25940.1	Symbols: ALPHA-VPE, ALPHAVPE	523	e-148
AT3G20210.1	Symbols: DELTA-VPE, DELTAVPE	410	e-114
AT3G20210.2	Symbols: DELTA-VPE	406	e-113

Amborella* Gene *AmTr_262-2

MATSGLRRSLFYFLLLSLLLFRQRSFGVRVNEWVDSVIRMPSQKEGGEGVGTRWAVLVAGSSGFGNYRHQADVCHAYQLLLKGGLKEENIVVFMHDDIAYNEFNPKK
GIIINHPQGEDVYAGVPKDYTGKQVHTKNLYAVLLGNNSAVTGGSGKVINSKAEDRIFIYYSDHGGPGVLGMPNMPFLYANDLMEVLKKKHKSKGYKEMVIYVEACE
SGSIFEGLMTEDLNIYVTTASNAQESSWGTYCPGMDPPPPPEFMTCLGDLYSVAWMEDSETHNLKEETIQKQYVRVKSRTSNYNTYTAGSHVMEYGDKSIKSERLYL
YQGFDPANANLSDNSLPLQPNRMDVVNQRDADLLFLWQRYERSTEGSEEKVAIRNEMTEKMAHREHLDKSVDLIGRLLFGWDKGSNVLGAKRPSGKALVDDWSCLKS
MVRAFEEKCGPLTQYGMKHMRAFANICNEGISLEVMSKACEEVCGRTYKHGILNVASHHGFSG

Arabidopsis *sequences producing significant alignments*:		Score (bits)	E Value
AT1G62710.1	Symbols: BETA-VPE, BETAVPE	626	e-179
AT4G32940.1	Symbols: GAMMA-VPE, GAMMAVPE	541	e-154
AT2G25940.1	Symbols: ALPHA-VPE, ALPHAVPE	524	e-149
AT3G20210.1	Symbols: DELTA-VPE, DELTAVPE	410	e-115
AT3G20210.2	Symbols: DELTA-VPE	406	e-113

Amborella* Gene *AmTr_36.100

MAFSGKSVLFLAVFMAFSGVYGRYSTWSDFLRMPQTEDSVGTRWAVLVAGSSGYGNYRHQADICHAYQTMIRGGLKEKNIVVFMYDDIAYNEENPRPGVIINRPHGE
DVYAGVPKDYVGDDVNVDNLFAVILGNKSALTGGSGKVVDSGPDDHIFIFYSDHGGAGVLGMPTYPYLYADDLVNVLKKKHVSGTYKSLVFYLEACESGSIFEGLLP
EGLNIYATTASNAVESSWGTYCPDDSPDFPQEYDTCLGDLYSVSWMEDSDIHNLQFETLKQQYELVKMRTSNFETYMFGSHVMQYGDSGLGKEQLVLYMGSNPANDN
STFISRNELPSFSKAVNQRDADLVYFWNKYRKSPVGSIKKRNAQKELFDVMAHRLHLDNSIELIGKLLFGSEKGPEILKTVRTTGLPLVDDWDCLKAMVRTFETKCG
SISQYGMKHMRSMANICNAGISKEVMAEASAEACTRIPTTS

Arabidopsis *sequences producing significant alignments*:		Score (bits)	Value
AT4G32940.1	Symbols: GAMMA-VPE, GAMMAVPE	661	0.0
AT2G25940.1	Symbols: ALPHA-VPE, ALPHAVPE	640	0.0
AT1G62710.1	Symbols: BETA-VPE, BETAVPE	578	e-165
AT3G20210.1	Symbols: DELTA-VPE, DELTAVPE	452	e-127
AT3G20210.2	Symbols: DELTA-VPE	447	e-126

B) Amino acid sequences of the plant VPEs used to construct the phylogenetic tree of Figure 2 and Supplemental Figure S3.

Phylogenetic trees of plant Vacuolar Processing Enzymes (VPE). The amino acid sequences used to construct the phylogenetic tree in Figure 2 and Supplemental Figure S3 are listed. For a detailed tree, see Supplemental Figure S3. For a simplified tree, see Figure 2. For the clarity of presentation, a simplified nomenclature has been used in Supplemental Figure S3 consisting of a three-letter code for the species followed by the accession number of the VPE protein (e.g., Ptr-gi224141591 for a Populus trichocarpa VPE).

Databases used to retrieve the VPE amino acid sequences. Short VPE names indicated on the detailed tree of Supplemental Figure S3 are shown. Sequences labeled with NCBI accession number, eg., >gi:gi:xxxxxx were retrieved from NCBI.
Other sequences, eg., >MA_XXXX (*Picea abies*), >PITA_VVVVV (*Picea taeda*) were retrieved at http://congenie.org.

VPE amino acid sequences:

Non seed plant VPEs

>gi:168013224 VPE Physcomitrella patens
MALSVEGIFLILLCSLGAMAVAAREWDGKIVMPTEEGSKDPQPTEDGQRWAVLVAGSSGYGNYRHQADVCHAYQILKKGGMKDENIVVFMFDDIAHNRHNPRPGVIL
NHPNGEDVYHGVPKDYTGKNVTVNNLLAVLLGDKKTLKGGSGKVVNSGPNDHIFIYYSDHGGPGVLGMPTNPNLYADDLLKTFKKMHEAKTYKEMVFVVYIEACESG
SIFQGLLPKDLNIYATTAANAEESSWGTYCPGMFPAPLEEFDTCLGDLYSVAWMEDTEVENLKKETLRDQYMIVKSRTSNHNTYKSGSHVLEFGDLKMKPEELDQYL
GYDPANENVTGPIFLREYLAIRLGGVEERHINQRDADLVHYWHRYHKSKVGSTAKAEAELDLMRILSHRMYIDKSVDLVGRLLFGVEAGPTTLSAVRPDGLPLTDDW
ACLKSMVSAFELSCGELSEYGMKHMRAFANICNAGVEPSKMSGVAAEACAVSAFGSGTLQIPTTGFSA

>gi:168033758 VPE Physcomitrella patens
MPTGEGHKGEEGTRWAILIAGSAGYWNYRHQADVCHAYQILKRGGLKDENIIVFMHDDIAYHPENPYPGTIINKPDGPDVYQGVPKDYTGSDVTVSNLYAAILGDKS
AIEGGTGKVVDSGPNDHIFIYYSDHGGPGVLGMPNPPNLYADDFVGILKKKAAAGTFKELVIYLEACESGSIFEGLLPEGLNIYVTTASNAVESSWGTYCPGMYPSP
PSEYGTCLGDLYSVAWMEDTEKENLKKETLEDQYLIVKSRTSNHNTYRSGSHVMQYGDLKIDVEELERYLGFDPANENVTKPGLSELSPVNSDIVTHVPQREADLVH
LKHKFYNAKKGSLREANAASELAKTILHRRHLDDSVRLIGELLFAGEDALQKLGAVRPAGSVVVDDWACLKNMVRIFEASCGPLTQYGMKHMRAFANICNAGINSSR
MSLASLEVCKISTSVDLGIWSPVTSGFSA

>gi:168005016 VPE Physcomitrella patens
MPTEEDAEKGTRWAILIAGSSGYWNYRHQADVCHAYQILKRGGLKEENIVVFMYDDIAYSTENPHPGKIINKPDGPDVYQGVPKDYTGADVTVSNFYAALLGDKDAI
KGGSGKVVNSGPNDHIFIYYTDHGGAGVLGMPTSPNLYADDFVDTLKKKAAGTFKELVIYLEACESGSIFEGLLPEGLNIYVTTASNAEESSWGTYCPGMYPPPPP
EYDTCLGDLYSVAWMEDTEIENLKKETLEDQYVIVKSRTSNHNTYRTGSHVMQYGDVKLDVEELARYLGYDPANENVTKPELPEFLSAHTEILTHVDQREADLIHLR
YKFRNAVKGSLREANAATELAKTIVHRKHLDDSVQLIGEILFAGENALEKLTAVRPAGSVVVDDWACLKTMVRTFEASCGPLTQYGMKHMRAFANICNARIDPAKMA
VASSEACKLSTSAGSGIWSPVTSGFSA

>gi:168065024 VPE Physcomitrella patens
MPIDFNSELKGVRWAILIAGSSGYGNYRHQADICHAYQILKRGGLKEENIVVFMYDDIANNEENPHRGKVFNKPYGPDVYPGVPKDYTGENITVSNFYAAILGDADA
TKGGSGKVVASGPNDHVFIYYADHGGAGVLGMPNDPILYADEFVDTLKKKAAAGTFKKMVIYVEACESGSIFDGLLPTGLNIYVTTASDPDENSWGTYCPTMIPPPP
PEFGTCLGDLYSVSWMEDAEMENLKKETLNDQYRIVKSRTSDNDTYMTGSHVMQYGDIEIDAEEVERYLGFDPANENVTRPELPVSKAPATASGMHVMQREAELLHL
WHKYHKAVDGSKKESAGMELTRTIAHRMHVDNSIKLIGDHMFGLDTSLLRLKAVRPAGQVLVDDWSCLKAMVRTFEASCGPLTQYGMKHMRAFASICNAGIDLDTMK
KATSQACGFSETDTDLRTADSPRFSASAKEFVKTIEF

>SM00084G00480 Legumain Selaginella moellendorffii
MPSSEEVASGTRWALLVAGSSGFGNYRHQADVCHAYQLLRSNGLKEENIVVMMFDDIANNTDNPRPGTIINHPQGSDVYAGVPKDYTGAAVTAENFLAVLLGDKNST
SGGTGKVVSSGPEDHVFLFYSDHGGPGVLGMPGESNLYANDLIDVIKKKHASGGYREMVIYIEACESGSMVEGLLPLGLGLYVTTASNAIESSWGTYCPGMVPSAPP
EYDTCLGDLYSVAWMEDSEVHNLKRETLLQQYLDVKDRTSNHNTYEAGSHVMQYGDVELNSNPLSMFLGFDPAIADGNGDLIIPSSANGVSQRDADLLHLWSKYRRA
KDGSDSKREARERMMNALAHRQHVDESVDRVGERLFGSKAAASKVLSTVRGSGLALVDDWTCLKSLASVQAFETSCGLLGQYGMKHMRAFANLCNEGVDVPRMAAAS
AEACSSTAQAL

>SM00000G12900 Legumain Selaginella moellendorffii
MGRFRLECVLWLLILAQQACAARKFEWKRDSSASDEFLEDQSARGTRWAVLLAGSAGYWNYRHQADVCHAYQLLRRGGMREENIVVFMYDDIANNFANPRPGVMINH
PNGDNVYAGVPKDYTGDQVTVNNFLAVLRGDKEALQGGSGKVVESGPNDHIFVFYSDHGGPGVLGMPVTPYLYAVDLVTTLQDMHDNNKYKEMVLYIEACESGSIFE
GLLPKNLNIFVTTASNAVESSWGTYCPGMEPSPPPEYDTCIGDLYSVAWMEDSEVHNLDHERLKDQYNTVKARTSDANTYRMGSHVMKYGDTNMDKERLSLYLGFDP
ANANLTSYNKPASSIGQRDADLLHFWQKYKNSKENSLEKSKALQEFLDVIGRRTQIDRSVELVGSVLLGSESASQILNSVRPEGHPLVDNWDCLKEMVRVFETKCGP
LGQYGMKHMRAFANLCNAGVDPERMKSAAGATCGGISYVDGHDTASHSSF

Gymnosperm VPEs

>MA_60392g0010 VPE Picea abies
MGFVINIVLYCVLFLGLNGVLLYARPDDEFAGNNVIRMPIYKQERHPSESKEEDEGGSISTRWAVLLAGSSGYGNYRHQADVCHAYQILRKGGLKEENIIVFMYDDI
ANNSDNPRPGTIINHPNGKDVYAGVPKDYTGDNVNVNNFLAVLLGDKSSVKGGSGKVVDSGPNDHIFIYYSDHGGPGVLGMPIPPYLYAHDLVEVLKKKHAAGAYKE
MVIYIEACESGSIFEGLLPKGLNIYVTTAANGEESSWGTYCPGMYPPPPPEYETCLGDLYSVAWMEDSEKHNLKTETIKQQYQLVKFRTSDHNTYQAGSHVMQYGDI
PISKEHLFLYIGSDPANANATFIYDNGFPEFPDEKDVMRAVNQRDADLLYLWQKYKRSKEGSIEKLESQKHMVDLMTHRMHLDKSVNLIGKLLFGSVRGLNVLNTVR
PPGQPLVNDWDCLKTMVRTFEKHCGSLSQYGMKHMRSLANICNEGVTKNTMAVVSAEACNQMSSRFRTSLHRGFSA

>MA_86205g0010 VPE Picea abies
MFSVSLFLLFCLLGFHVIVDASRFHFDSKIRLSTAGEDEDIGTQWAVLLAGSAGYSNYRHQADVCHAYQILKRGGLKDENIVVFMYDDIANNPVNPRPGIIINHPEG
SDVYAGVPKDYTGKEVTVDNFFAVILGDKDSVKGGSGKVVDSGPNDHIFIYYTDHGGPGVLGMPSGHMLYAKDLIDVLKKKHAADTYKQMVIYVEACESGSIFEGLL
PEGMNIYVTTASNAEESSWGTYCPGMKPSPPLEYDTCLGDLYSVAWMEDSEVHNTMKETLKQQYQVVKERTSNHQTYGMGSHVMQYGDIPISEDPLSLYIGFDPANA
DAIFENRLPQYLREKDAAAINQRDADLLYLWQKYKRSKPDSTEKLEAQQELIESMSHRLYLDKSINFIGKILFGSDTGTAVLNAVRPSGQPLVDDWDCLKTMVRTFE
SHCGSLSQYGMKHVRALANICNNGVSVDTMAEVSAEACTQISSGKWRSLQVGYSA

>gi:148910236 C13 peptidase Picea sitchensis
MGSSKGFQLGLLCYFLLLSLDSSKVADGARRDWNSLLKLPTNHVDADSDRIGTEWAVLLAGSSGYWNYRHQADVCHAYQILRRGGLKEENIVVFMYDDIAYDEENPH
PGTIINHPQGSDVYAGVPKDYTGEDVTVNNFFAAILGNKSLVTGGSGKVVESGPNDRIFIYYSDHGGPGVLGMPLPPYLYANDFVQVLKKKHDAGSYREMVIYVEAC
ESGSIFEGLLPTDLNIYVTTASNAEENSWGTYCPGMDPPPPPEYDTCLGDLYSVAWMEDSEINNLKEETLLQQYDLVKLRTSNHNTYMSGSHVMQYGNITISQEELY
LYMGFDSANSNASLVLENSPLLEKTEAKAINQRDADLLYMWQKYKKSKEDSPERLTAQTQLLEFMAHRMHVDKSVKLVGNLLFGPEKGPAVFNAVRPQGEPLVDDWD
CLKKMVRTFEGHCGSLAQYGMKHMRALANICNEGISMDTMATVSAEACTQFPAGSWSSLQRGFSA

>gi:116789977 C13 peptidase Picea sitchensis
MSPVMFSVSLFLLFCLLGFHVIVDASRFHFDSKIRLSTEGEDENIGTQWAVLLAGSAGYSNYRHQADVCHAYQILKRGGLKDENIVVFMYDDIANNPVNPRPGIIIN
HPEGSDVYAGVPKDYTGKEVTVDNFFAVILGDKDSVKGGSGKVVDSGPNDHIFIYYTDHGGPGVLGMPSGHMLYAKDLVDVLKKKHAADTYKQMVIYVEACESGSIF
EGLLPEGMNIYVTTASNAEESSWGTYCPGMKPSPPLEYDTCLGDLYSVAWMEDSEVHNTMKETLKQQYQVVKERTSNHQTYGMGSHVMQYGDIPISEDPLSLYIGFD
PANADAIFENRLPQYLREKDAAAINQRDADLLYLWQKYKRSKPDSTEKLEAQQELIESMSHRLYLDKSINFIGKLLFGSDMGTAVLNAVRPSGQPLVDDWDCLKTMV
RTFESHCGSLSQYGMKTHACSSKYL

>PITA_000008627 VPE Picea taeda
MMRLCTSAMGSSKGFQLGLLCYFLLLLLDSSKVADGARRGWDSVLKMPTDHIDDDSDAIGTQWAILLAGSSGYWNYRHQADICHAYQILRKGGLKEENIVVFMYDDI
AQDEENPNPGTIINHPQGSDVYAGVPKDYTGENVTVNNFFAVLLGNKSLVTGGSGKVVESDPNDRIFIYYSDHGGPGVLGMPLPPYLYANDFIQVLKQKHAAGSYRE
MVIYIEACESGSIFEGLLPTDMNIYATTASNAEENSWGTYCPGMDPPPPPEYDTCLGDLYSVAWMEDSEMNNLKEETLLQQYNLVKRRTSNHNTYMTGSHVMQYGNL
TISQEELYLYIGFDSANSNASLVLENTLLLERTEAKAINQRDADLLYMWQKYKKSKEDSPERFTAQTQLLEFMAHRMHVDKSVKLVGNLLFGPEKGSAVLKAVRPQG
EPLVDDWDCLKKMVRTFERHCGSLAQYGMKHMRALANICNEGISMDTMATVSAEACTQFPAGSWSSLQRGYSA

>PITA_000069534 VPE Picea taeda
MGFINNMVIVLSCVLFLGLNGVLLHARPDEFTGDGFIRMPVDKQHSFESKEEEDGDSIGTRWAVLLAGSSGYGNYRHQADVCHAYQILRKGGLKEENIIVFMYDDIA
NNSDNPRPGTIINHPNGKDVYAGVPKDYTGDNVNVNNFLAVLLGDKSSVKGGSGKVVDSGPNDHIFIYYSDHGGPGVLGMPIPPYLYAHDLVEVLKKKHAANAYKEM
VIYIEACESGSIFEGLLPKGLKIYVTTAANGEESSWGTYCPGMYPPPPPEYETCLGDLYSIAWMEDSEKHNLKTETIKQQYQLVKFRTSDHNTYRAGSHVMQYGDIP
ISKEQLFLYIGSDPANANATFTYDNGFPEFPDEKDVRAVNQRDADLLYLWQKYKRSKEGSIKKLESQRQMVDLMTHRMHVDKSMNLIGKLLFGSARGPNVLNTVRPP
GQPLVDDWDCLKTMVRTFEKHCGSLSQYGMKHMRSLANMCNEGVTKNTMTAVSAEACNQMPSRFRASLHRGFSA

>PITA_000008629 VPE Picea taeda
MGSVMFSLCLFLLFCLLGFHGIGIVDASRLHFDSKIRLSTDGKDENLGTQWAVLLAGSAGYSNYRHQVIFCMVVPTKVCIMQADVCHAYQILKRGGLKDENIIVFMY
DDIANNPANPRPGIIINHPEGSDVYAGVPKDYTGKEVTVDNLFAVILGDKDSVKGGSGKVVDSGPNDHIFIYYTDHGGPGVLGMPIGPSLYANDLIDVLKKKHAADS
YKQMVIYVEACESGSIFEGLLPEGMNIYVTTASNAEESSWGTYCPGMEPSPPLEYDTCLGDLYSVAWMEDSEVHNTMKETLDQQYQVVKERTSNHHTYGMGSHVMQY
GDIPISEDPLSLYIGFDPANANVTFENSLPQYLREKDATVINQRDADLLYLWQKYKRSKPDSTEKLEAQQELIESMTHRLHLDKSINFIGKLLFGSEMGTTVLKAVR
PSGQPLVDDWDCLKTMVIFKCSMVICISSVLQPTRL

Amborella alpha/gamma/delta VPE pro-ortholog

>36.100 Amborella trichopoda
MAFSGKSVLFLAVFMAFSGVYGRYSTWSDFLRMPQTEDSVGTRWAVLVAGSSGYGNYRHQADICHAYQTMIRGGLKEKNIVVFMYDDIAYNEENPRPGVIINRPHGE
DVYAGVPKDYVGDDVNVDNLFAVILGNKSALTGGSGKVVDSGPDDHIFIFYSDHGGAGVLGMPTYPYLYADDLVNVLKKKHVSGTYKSLVFYLEACESGSIFEGLLP
EGLNIYATTASNAVESSWGTYCPDDSPDFPQEYDTCLGDLYSVSWMEDSDIHNLQFETLKQQYELVKMRTSNFETYMFGSHVMQYGDSGLGKEQLVLYMGSNPANDN
STFISRNELPSFSKAVNQRDADLVYFWNKYRKSPVGSIKKRNAQKELFDVMAHRLHLDNSIELIGKLLFGSEKGPEILKTVRTTGLPLVDDWDCLKAMVRTFETKCG
SISQYGMKHMRSMANICNAGISKEVMAEASAEACTRIPTTS

Monocot alpha/gamma/delta VPE pro-orthologs

>gi:475545141 VPE Aegilops tauschii
MAMASFRPLPLALLLAACLSAFVLAVAHARTPGLDPTIRLPSQRAAGQEDDDSVGTRWAVLIAGSNGYYNYRHQADICHAYQIMKKGGLKDENIIVFMYNDIAHNPE
NPRPGVIINHPQGGDVYAGVPKDYTGKEVNVKNFFAVLLGNKTAVSGGSGKVVDSGPNDHIFVFYSDHGGPGVLGMPTYPYLYGDDLVDVLKKKHAAGTYKSLVFYL
EACESGSIFEGLLPNDIGVYATTASNAEESSWGTYCPGEYPSPPPEYDTCLGDLYSISWMEDRANLMAMELGASFAVNAPCRNSCSCCTQPYSYEIATEHHLDVHNL
RTESLKQQYNLVKKRTAAQDSYSYGSHVMQYGSLDLNAEHLFSYIGSNPANENTTFVEDNALPSFSRAVNQRDADLVYFWQKYRKLAESSPEKNDARKQLLEMMGHR
SHIDNSVELIGNLLFGSAGGPMVLKAVRPAGEPLVDDWSCLKSTVRTFESQCGSLAQYGMKHMRSFANICNAGIVPEAMAKVAAQACTSIPTNPWSATHKGFSA

>gi:357135238 VPE Brachypodium distachyon
MASFRLLPLALLLCACLSAHARTSLLEQTIRLPSQRGAAGQQEVDDDSVGTRWAVLIAGSNGYYNYRHQADICHAYQIMKKGGLKDENIIVFMYDDIAHNPENPRPG
VIINHPQGGDVYAGVPKDYTGKEVNVKNFFAVLLGNKAAVSGGSGKVVDSGPNDHIFVFYSDHGGPGVLGMPTYPYLYGDDLVDVLKKKHAAGTYKSLVFYLEACES
GSIFEGLLPNDIGIYATTASNAEESSWGTYCPGEYPSPPPEYDTCLGDLYSIAWMEDSDVHNLRTESLKQQYDLVKKRTAPENSYSYGSHVMQYGSLDLNAEHLFLY
IGSNPANDNTTFVEGNSLPSFSRAVNQRDADLVYFWQKYRKLAESSPAKNDARKELLEMMAHRSHVDNSVELTGNLLFGSEDGPMVLKTVRTAGEPLVDDWGCLKST
VRAFESQCGSLAQYGMKHMRSFANICNAGILPEATAKVAAQACPSIPANPWSATHKGFSA

>gi:194352742 Legumain Hordeum vulgare
MAMASFRLLPLALLLSVAHARTPRLEPTIRLPSQRAAGQEDDDSVGTRWAVLIAGSNGYYNYRHQADICHAYQIMKKGGLKDENIIVFMYDDIARNPENPRPGVIIN
HPQGGDVYAGVPKDYTGKEVNVKNFFAVLLGNKTAVNGGSGKVVDSGPNDHIFVFYSDHGGPGVLGMPTYPYLYGDDLVDVLKKKHAAGTYKSLVFYLEACESGSIF
EGLLPNDIGVYATTASNAEESSWGTYCPGEYPSPPPEYDTCLGDLYSISWMEDSDVHNLRTESLKQQYNLVKKRTAAQDSYSYGSHVMQYGSLDLNAEHLFSYIGSN
PANENTTFVEDNALPSLSRAVNQRDADLVYFWQKYRKLAESSPAKNNARKQLLEMMGHRSHIDSSVELIGNLLFGSAGGPMVLKTVRPAGEPLVDDWSCLKSTVRTF
ESQCGSLAQYGMKHMRSFANMCNAGIVPEAMAKVAAQACTSFPTNPWSATHKGFSA

>gi:573916715 VPE Oryza brachyantha
MAPAPRLLPLLPPLAALLLCAHLAVAVARPRMEPWGSRGPRMEPWDSSDNLLLPSEREWERERERSVEDEDAAEAAVGTRWAVLIAGSNGYYNYRHQADVCHAYQIM
KKGGLKDENIIVFMYDDIAHNSENPRPGVIINHPQGGDVYAGVPKDYTGKDVNVKNLFAVLLGDKTAVSGGSGKVLDSGPNDHIFIFYSDHGGPGVLGMPTYPYLYG
DDLVDVLKKKHAAGTYKSLVFYLEACESGSIFEGLLPDDINVYATTASNADESSWGTYCPGEYPSPPPEYDTCLGDLYSVAWMEDCDVHNLRTESLRQQYNLVKERT
SVQHTYDSGSHVMQYGSIELNAHHLFLYMGSNPANDNSTFVEDNSLPSFSRAVNQRDADLVYFWQKYRKLAEGSPEKNEARKQLLEMMAHRSHVDNSVELIGNLLFG
SEEGPRVLKAVRATGEPLIDDWSCLKSMVRAFEAQCGSLAQYGMKHMRSFANICIAGISAEEMAKVAAQACTSIPSNPWSSTHRGFSA

>gi:115437636 C13 peptidase Oryza sativa Japonica
MAARARLRLVLPPLAALLLFAHLAAVAVARPRWEEEGSNLRLPSERAVAAGAAADDAAEAAEGTRWAVLIAGSNGYYNYRHQADVCHAYQIMKRGGLKDENIIVFMY
DDIAHNPENPRPGVIINHPQGGDVYAGVPKDYTGKEVNVKNLFAVLLGNKTAVKGGSGKVLDSGPNDHIFIFYSDHGGPGVLGMPTYPYLYGDDLVDVLKKKHAAGT
YKSLVFYLEACESGSIFEGLLPNGINVYATTASNADESSWGTYCPGEYPSPPPEYDTCLGDLYSVAWMEDSDVHNLRTESLKQQYNLVKERTSVQHTYYSGSHVMEY
GSLELNAHHVFMYMGSNPANDNATFVEDNSLPSFSRAVNQRDADLVYFWQKYRKLPESSPEKNEARKQLLEMMAHRSHVDNSVELIGNLLFGSEEGPRVLKAVRATG
EPLVDDWSCLKSMVRTFEAQCGSLAQYGMKHMRSFANICNAGISAEAMAKVAAQACTSIPSNPWSSTHRGFSA

>gi:115465809 C13 peptidase Oryza sativa Japonica
MGRGLLCLLLLQLVGLVVAGGGRWRWQEEFLRLPSSDETTRWAVLIAGSNGFYNYRHQADVCHAYQIMRKGGVEEQNIVVMMYDDIAHNPDNPRPGLIFNHPSGPDV
YAGVPKDYTGDDVNVNNFLAVLLGNRSALTGSGSGKVVASGPNDHVFVYYADHGGPGVLSMPADGEYLYADDLVKALKKKHAGGGYKSLVVYVEACESGSIFEGLLP
SDISVYATTASNAEESSWGTYCPGDDHDAPAAEFDTCLGDLYSVAWMEDAEAHQEGRLAETLRQQYRTVKNRTSDEGTYTLGSHVMQYGDMALAPQSLDLYYMDTSP
ATANDHKLAAAGAKGSHSYTVSVNQRDADLLYLWRKYRRAGEGTAEKVEARERLVQEMGRRSRVDRSVEMIGGLLLGGAKHKQQVVRERAALVEDWECLRSMVRTFE
DQCGSLGQYGIKHMRSFANICNAGVPHHAMAKAASLACPSPPPLHL

>gi:6907094 C13 peptidase Oryza sativa Japonica
MAMGVLLLMLMLMHLQVGLGLGNGGLWQEFLRLPTENGGTKWALLIAGSKGYENYRHQADVCHAYQIMKKGGLKDQNIVVMMYDDIAYNPENPHKGVIINKPNGPNV
YAGVPKDYNGNDVNKNNFLAVLLGKKSALTGAGSGKVISSGPNDHIFVYYSDHGSPGYVCMPSGGNLHANDLSQALKNKNAAGAYKNLVVYVEACESGSMFEGQLLP
SNIGVYAMTASNATENSWATYCDTPEYNTCLGDLFSVAWMEDADARRPGDPETLGQLYDIVAKRTNLSHVSRYGDLSLSSQPVSLYYLPPGPGTSTASAVIDDEGRV
GGVNQRDAGLVYLWRKYYEEKSVEAWERLLREMERRSRVDSSVDLIGDILLGDSSKKKLLHIRRPAGQPLVDDWDCLKSMVRTFEAHCGPLGQYGMKHTRAFANMCN
AALDHNHMAKAASKACMHPPVITY

>gi:373254763 Legumain Saccharum hybrid cv
MVAARLRLALLLLSVCLCSAWARPRLEPTIRLPSERAAAAAGDETDDAVGTRWAVLVAGSSGYYNYRHQADICHAYQIMKKGGLKDENIIVFMYDDIAHSAENPRPG
VVINHPQGGDVYAGVPKDYTGRQVSVNNFFAVLLGNKTALTGGSGKVVDSGPNDHIFVFYSDHGGPGVLGMPTYPYLYGDDLVDVLKKKHAAGTYKSLVFYLEACES
GSIFEGLLPDDINVYATTASNAEESSWGTYCPGEFPSPPPEYDTCLGDLYSVSWMEDSDFHNLRTESLKQQYKLVKDRTAAQDTFSYGSHVMQYGSLELNVQKLFSY
IGTNPANDGNTFVEDNSLPSFFKSCNQRDADLVYFWQKYRKLADGSSKKNEARKELLEVMSHRSHVDNSVELIGSLLFGSEDGPRVLKAVRAAGEPLVDDWSCLKSM
VRTFEAQCGSLAQYGMKHMRTFANICNAGILPEAVSKVAAQACTSIPSNPWSSIDKGFSA

>gi:93139442 Legumain Saccharum officinarum
MVTARLRLALLLLSVFLCSAWARPRLEPTIRLPSERAAAAAGDETDDAVGTRWAVLVAGSSGYYNYRHQADICHAYQIMKKGGLKDENIIVFMYDDIAHSAENPRPG
VVINHPQGGDVYAGVPKDYTGRQVSVNNFFAVLLGNKTALTGGSGKVVDSGPNDHIFVFYSDHGGPGVLGMPTYPYLYGDDLVDVLKKKHAAGSYKSLVFYLEACES
GSIFEGLLPDDINVYATTASNAEESSWGTYCPGEFPSPPPEYDTCLGDLYSVSWMEDSDFHNLRTESLKQQYKLVKDRTAAQDTFSYGSHVMQYGSLELNVQKLFSY
IGTNPANDGNTFVEDNSLPSFSKAVNQRDADLVYFWQKYRKLADGSSKKNEARKELLEVMSHRSHVDNSVELIGSLLFGSEDGPRVLKAVRAAGEPLVDDWSCLKSM
VRTFEAQCGSLAQYGMKHMRTFANICNAGILPEAVSKVAAQACTSIPSNPWSSIDKGFSA

>gi:242053313 C13 peptidase Sorghum bicolor
MVAARLRLALLLLLPVFLCSAWARPRLEPTIRLPSDRADDAVGTRWAVLVAGSNGYYNYRHQADICHAYQIMKKGGLKDENIIVFMYDDIAHSPENPRPGVLINHPQ
GGDVYAGVPKDYTGREVSVNNFFAVLLGNKTALKGGSGKVVDSGPNDHIFVFYSDHGGPGVLGMPTYPYLYGDDLVDVLKKKHAAGTYKSLVFYLEACESGSIFEGL
LPDDINVYATTASNAEESSWGTYCPGEFPSPPPEYDTCLGDLYSVSWMEDSDFHNLRTESLKQQYKLVKDRTAVQDTFSYGSHVMQYGSLELNVQKLFSYIGTNPAN
DGNTFVEDNSLPSFSKAVNQRDADLVYFWQKYRKLADDSSKKNEARKELLEVMAHRSHVDNSVELIGSLLFGSEDGPRVLKAVRAAGEPLVDDWSCLKSMVRTFEAQ
CGSLAQYGMKHMRSFANICNAGILPEAVSKVAAQACTSIPSNPWSSIDKGFSA

>gi:356463706 VPE4 Triticum monococcum
MAMASFRPLPLALLLAACLSALVLAVAHARSPRLEPTIRLPSQRAAGQEDDDSVGTRWAVLIAGSNGYYNYRHQADICHAYQIMKKGGLKDENIIVFMYDDIAHNLE
NPGPGVIINHPQGGDVYAGVPKDYTGKEVNVKNLFAVLLGNKTAVSGGSGKVVDSGPNDHIFVFYSDHGGPGVLGMPTYPYLYGDDLVDVLKKKHAAGTYKSLVFYL
EACESGSIFEGLLPNDIGVYATTASNAEESSWGTYCPGEYPSPPPEYDTCLGDLYSISWMEDSDVHNLRTESLKQQYNLVKKRTAAQDSYSYGSHVMQYGSLDLNAE
HLFSYIGSNPANENTTFVEDNALPSFSRAVNQRDADLVYFWQKYRKLAESSPEKNDARKQLLEMMGHRSHIDNSVELIGNLLFGSAGGPMVLKAVRPAGEPLVDDWS
CLKSTVRTFESQCGSLAQYGMKHMRSFANICNAGIVPEATAKVAAQACTSIPTNPWSATHKGFSA

>gi:195624628 VPE Zea mays
MVAARLRLALLLSVCLCSAWARPRLETAIRLPSQRAAAADETDDGAVGTRWAVLIAGSSGYYNYRHQADICHAYQIMKKGGLKDENIIVFMYDDIAHSPENPRPGVI
INHPQGGDVYAGVPKDYTGRDVNVDNFFAVLLGNKTALRGGSGKVVDSGPDDHIFVFYSDHGGPGVLGMPTYPYLYGDDLVDVLKKKHAAGTYKSLVFYLEACESGS
IFEGLLPNDINVYATTASNAEESSWGTYCPGEFPSPPPEYDTCLGDLYSVAWMEDSDFHNLRTESLKQQYKLVKDRTAVHDTFSYGSHVMQYGALELNVQRLFSYIG
TDPANDGNTFIEDNSLPSFSKAVNQRDADLVYFWQKYRKLADSSPPKSEARKELLEVMAHRSHVDSSVELIGSLLFGSEDGPRVLKAVRAPGEPLVDDWSCLKSIVR
TFEARCGSLAQYGMKHMRSFANMCNAGILPEAVSKVTAQACSSIPSNPWSSIHKGFSA

>ZM08G11940 VPE Zea mays
MASRRLLFAVQLLVLIAAVAGTRWQDFLRLPSESESVGTRWAVLIAGSNGYYNYRHQVVISSITLSLCFATTLVEQILLHAYIHIHGQADVCHAYQVLKKGGLKDEN
IVVFMYDDIADSPDNPRPGVIINHPSGGDVYAGVPKDYTGKDVNANNFLAALLGNRSAVTGGGSGKVVASGPADHVFVYYSDHGGPGVLGMPSSDDYLYAKDLVDAL
RKKHAAGGYRSLVFYLEACESGSIFEGLLPPDIAVYATTAANAEESSWGTYCPGDDPGPPPEFDTCLGDLYSVAWMEDSDARRDRRAETLRQQYLAVKDRTSAHGTY
SLGSHAMEYGDVQGLGAQSLYTFMGSDDATAASLSGRGRGQPAVSQRDADLVYFWRRYRRAAERTPEKAEARTRLLRAVSRRSRVDSIMELIGGLLFGSEGGPRVLG
AVRPAGQPLADDWDCLKSLVRAYERSCGPLGQYGMKHMRGFANICNAGVGEDGMAKVASEACAAVARSDD

>gi:6634705 Legumain Zea mays
MVADRLRLALLLSACLCSAWARPRLEPTIRLPSDRAAADDAVGTRWAVLIAGSNGYYNYRHQADICHAYQIMKKGGLKDENIVVFMYDDIAHSPENPRPGVIINHPQ
GGGVPKDYTDVYAGRDVNVDNFFAVLLGNKTALRGGSGKVVDSGPNDHISVFYSDHGGPGVLGMPTYPYLYGDDLVDVLKKKHAAGTYKSLVFYLEACESGSIFEGL
LPNDINVYATTASNAEESSWGTYCPGEFPSPPPEYDTCLGDLYSVAWMEDSDFHNLRTESLKQQYNLVKDRTAVQDTFSYGSHVMQYGSLGLNVKHLFSYIGTNPAN
DDNTFIEDNSLPSFSKAVNQRDADLVYFWQKYRKLADSSPEKNEARRELLEVMAHRSHVDSSVELIGSLLFGSEDGPRVLKAVRAAGEPLVDDWSCLKSTVRTFEAQ
CGSLAQYGMKHMRSFANICNAGILPEAVSKVAAQACTSIPSNPWSSIHKGFSA

Basal eudicot alpha/gamma/delta VPE pro-ortholog

>gi:310771866 VPE Papaver rhoeas
MVKFLFSVIILFFLLSAVGSSARNIEEDGVIRLPSEVKDFINGKNIDDDSVGGTRWAVLIAGSSGYWNYRHQADVCHAYQVLKRGGVKDENIVVFMYDDIALNEENP
RPGVIINHPKGEDVYAGVPKDYTGRDVTAHNFYSVLLGNKTAVKGGSGKVIDSGPNDHIFIYYSDHGGPGVLGMPTYPYLYADDLVNVLKQKHALGAYKSLVFYLEA
CESGSIFEGILPKGLNIYATTASNAEESSWGTYCPGEFPSPPSEYETCLGDLYSVAWMEDSDVHNLRSETLKQQYHLVKERTQNANSAYGSHVMQYGDLEVSKEDLF
LYMGTNPANDNNKFIEQNSLPSLSGSVNQREADLIHFWQKYRKAPEGSQRKADAQKQFVEVMAHRMHVDHSIKLIGKLLFGFEKGPQVLEAVRPAGQPLVDDWDCLK

Core eudicot delta VPEs

>>gi:657378753 VPE Medicago truncatula MTR_7g079140
MNQIISCWGALITIVWMSVTVTLSKGVVRPMVHKHDEQGNFEVVGKKWALLVAGSKGYSNYRHQSNICHAYHILKSGGLQDENIIVFMYDDIAYHNENPRPGVIINR
PDGPNVYPGVPKDYTGNNTNAENFFAVLNGNLSGITGGSGKVLNSDPNDTIFIYYSGHGYPGLIGMADQSLVYAKDLVDALKKKHASNSYKKMVIYVEACYSASLFE
GLLPNNISIYVTTSANARELGYGFYCPGSINLSSTEYTTCLGDTFGISWMEDSDKNESTNETLQQQYVTVRDRTITSHVTQLGDLNISNDFLDTYIGSAPLNNVSDN
YNLTNTTSVYSFEPFNTSTSLVNQDDAYLLHLKLKLEKAVDGSKDKLKAQNELDAEIAHRKHVDHNIHLIGNILFGEKKSSIMMSDLRSAGQPLIDDWNCLKILFKT
YESHCGILLSTYGRKYSRVFAYMCNIGIFEKQTISAVSQVCSRIHHSS

>gi:355497280 VPE Medicago truncatula MTR_7g032230
MNHKNKYWVALIASIWMSVTDNVFAEGESTTGKKWAFLVAGSNGYVNYRHQADICHAYQILKKGGLKDENIVVFMYDDIAYNPQNPRRGVLINHPNGSDVYNGVPKD
YIGDYGNLENFLAVLSGNKSATKGGSGKVLDTGPDDTIFIFYTDHGSPGSIGIPDGGLLYANDFVDALKKKHDAKSYKKMVIYMEACEAGSMFEGLLPNDINIYVTT
ASNKSENSYGFYCPNSYLPPPPEYDICLGDLYSISWMEDSEKNDMTKEILKEQYETVRQRTLLSHVLQYGDLNISNDTLITYIGADPTNVNDNFNVTSTTNVFSFDD
FKSPNPTRNFGQRDAHLIYLKTKLGRASSGSEDKLKAQKELEVEIARRKHVDNNVHQISDLLFGEEKGSIVMVHVRASGQPLVDNWDCLKTLVKTYESHCGTLSSYG
RKYLRAFANMCNNGITVKQMVAASLQACLEKN

>gi:334185489 VPE delta Arabidopsis thaliana
MSSPLGHFQILVFLHALLIFSAESRKTQLLNDNDVESSDKSAKGTRWAVLVAGSNEYYNYRHQADICHAYQILRKGGLKDENIIVFMYDDIAFSSENPRPGVIINKP
DGEDVYKGVPKDYTKEAVNVQNFYNVLLGNESGVTGGNGKVVKSGPNDNIFIYYADHGAPGLIAMPTGDEVMAKDFNEVLEKMHKRKKYNKMVIYVEACESGSMFEG
ILKKNLNIYAVTAANSKESSWGVYCPESYPPPPSEIGTCLGDTFSISWLEDSDLHDMSKETLEQQYHVVKRRVGSDVPETSHVCRFGTEKMLKDYLSSYIGRNPEND
NFTFTESFSSPISNSGLVNPRDIPLLYLQRKIQKAPMGSLESKEAQKKLLDEKNHRKQIDQSITDILRLSVKQTNVLNLLTSTRTTGQPLVDDWDCFKTLVNSFKNH
CGATVHYGLKYTGALANICNMGVDVKQTVSAIEQASNQENRRNRRYETNIKDRLVSGELSDGSRPARDQNGVPRGHLAVYVGREERQRFVIPTKYLQYPEFRSLMDE
VADEFGYDHEGGIHIPCEESVFEEILIRYMSCDKKK

>Potri.008G003400 C13 peptidase Populus trichocarpa
MSSSSYLCGYGYGTFLFLIALLSSIAQSQGVIINSTSASSLPSSVRRDSTTAEGKQWAVLVAGSAGYENYRHQADVCHAYQILKKGGLKDENIIVFMYDDIAFHVDN
PRPGIIINKPFGHDVYAGVPKDYTGDNCTVDNLFAVLLGNKSALTGGSGKVVDSGPNDNIFIYYADHGAPGLVGMPIGKDLYAKDLIQVLKKQQEANSYKSMVFYLE
ACESGSMFEGLLPSNWSIYAITAANGEESSYGIYCPGYYPAPPPEFLTCLGDVFSISWMEDSDLHDMSQETLQQQYEVVRRRTGFDYEDRSHVMQYGNMELSKELLS
SYLGTNAANDNYATNINIEEYPSMIPRAFDQREATLLHFWHKYQEAPDGSDKKAEAHKDLLRIHSHIRHVDRSLSHIASTLFGDENAANAMKHVRPSGQPLVDDWDC
LKGLVEAYEKQCGGLSWYGKKYTRVIANMCNAGINVEQMIGASTRACSSRTTTTTPTRGSLQNEFDK

>VV09G09760 VPE Vitis vinifera
MNYYIVGILLFITLFSPFTKSEPPKLDRPSNETEFIHGNSKPNCKAAEPAKGKQWAVLIAGSTDYENYRHQADICHAYQILKKGGLKDENIIVFMYDDIAFNVENPR
PGVIINQPGGDDVYEGVPKDYTQSAATVANVFAVLLGNKTAVQGGSGKVLDSGPDDHVFIYYADHGATGIIGMTDGLIYAKDLIDVLKKKHEAKAYKTMVIYIEACE
AGSMFQGLLPNNWDIYATTAANAEENSYGTYCPDDYPSAPSEYDTCLGDTYSVAWLEDSEMHDLRFETLEKQYKTIRRRVFTQDLDFNSHVTQYGDMKLSKEFLFTY
MGTNPDNDNYTSMANSKPSGFSSASQYDAELLHFWYKFHRAPEGSTRKLEAQKELHRKISHRMHVDHSMKEIGKLILGSENSTMMLLKTVRPLDQPVVDDWDCYKML
VKTYEEHCGSLSRYGLKYTRALANMCNAGIKMEQMAVASAQACAKIKP

>VV04G07860 VPE Vitis vinifera
MTIFPAAVAAFLALSTLVAGGRHFAGDNGLLLPSEASRFFRPGGAADDDTGAESAGTRWAVLIAGSNGYWNYRHQADICHAYQLLKKGGLKDENIIVFMYDDISFNE
ENPRPGIIINSPHGEDVYEGVPKDYTGEDVTVDNFFAVILGNKTALSGGSGKVLDSGPNDHIFIYYSDHGGPGVLGMPTSPYLYANDLIEVLKKKHASGTYNSLVFY
LEACESGSIFEGLLPEGLNIYATTAANAEESSWGTYCPGEDPSPPPEYETCLGDLYSVAWMEDSDVHNLRTETLRQQYELVKKRTANDNSVYGSHVMQYGDLGLNKE
DLVLYMGTNPANDNYTFVDNNSLRLPSKAVNQRDADLVHFWDKFRKAPEGSPRKAEAQKQFLEAMSHRTHIDHAIKLVGRLLFGMKKGSEVLKTVRPAGQPLVDDWH
CLKTLVRTFEAHCGSLSQYGMKHMRSIANICNAGIEKEQMAEASAQACVTIPPGPWSSLDKGFSA

Core eudicot alpha/gamma VPE pro-orthologs

>gi:357437513 VPE Medicago truncatula MTR_1g016590
MDFSQFSTILFLTVILTIFAAVSGSRDLPGDYIRLPSQSQASRFFHEPENDDNDQGTRWAILLAGSNGYWNYRHQADVCHAYQLLRKGGLKEENIIVFMYDDIASNV
ENPRPGVIINKPDGGDVYEGVPKDYTGAEVHADNFYAALLGNKSALTGGSGKVVDSGPNDHIFVYYTDHGGPGVLGMPVGPYLYASDLNEVLKKKHASGSYKSLVFY
LEKISISMRQTASNAVESSWGTYCPGEYPPPPPEYSTCLGDLYSIAWMEDSDIHNLRTESLHQQYKLVKDRTINGYYGSHVMEYGDVGLSNNHLFLYLGTNPANDNI
SFVDESSLKLRSPSTAVNQRDADLIHFWDKFRKAPEGSLRKNEAQKEVLEAMSHRMHVDNSVKLIGKLLFGIEKGTELLDNVRPAGSPLVDNWDCLKTMVKTFETHC
GSLSQYGMKHMRSFANICNAGIQTEQMAEASA

>gi:502132679 VPE Cicer arietinum
MYRFPTPTLLFLIVTLIALVSSNPEDFLRLPSESSRFFHSPSADDKENNEGTRWAILIAGSNGYWNYRHQSDVCHAYQVLRKGGLKEENIVVFMYDDIAFNEENPRP
GVIINSPHGDDVYKGVPKDYTGEDVNVDNFFAALLGNKSALTGGSGKVVDSGPNDHIFIYYSDHGGPGVLGMPTSPYMYASDLIEVLKKKHASGTYKSLVFYLEACE
SGSIFEGLLPEGLNIYATTAANADESSWGTYCPGEFPSPPPEYETCLGDLYSVAWMEDSDMHNLQSETLHQQYELVKERTKNGNTLYGSHVMQYGDIGLSENSLFLY
LGTNPANENFTFVGRNSLVPPSKAVNQRDADLVHFWDKFRKAPQGSPRKAAAEKQVLEAMSHRMHIDDSIKLVGKLLFGMEKGPEVLTSVRPAGQPLADDWNCLKTL
VRTFETYCGSLSQYGMKHMRSFANFCNAGIHKEQMAEASAQACVNVPANPWSSLRSGFSA

>gi:351724961 VPE2 Glycine max
MPTFFLPTLLLLLIAFATSVSGRRDLVGDFLRLPSETDNDDNFKGTRWAVLLAGSNGYWNYRHQADVCHAYQILRKGGLKEENIIVFMYDDIAFNGENPRPGVIINK
PDGGDVYKGVPKDYTGEDVTVDNFFAALLGNKSALTGGSGKVVDSGPDDHIFVYYTDHGGPGVLGMPAGPYLYADDLIEVLKKKHASGTYKNLVFYLEACESGSIFE
GLLPEDINIYATTASNAEESSWGTYCPGEYPSPPPEYTTCLGDLYSVAWMEDSDRHNLRTETLHQQYKLVKERTISGDSYYGSHVMQYGDVGLSRDVLFHYLGTDPA
NDNFTFVDENSLWSPSKPVNQRDADLIHFWDKFRKAPEGSLRKNTAQKQVLEAMSHRMHVDNSVKLIGKLLFGIEKGPEVLNAVRPAGSALVDDWHCLKTMVRTFET
HCGSLSQYGMKHMRSFANICNVGIKNEQMAEASAQACVSIPSNPWSSLQRGFSA

>gi:356564077 VPE Glycine max
MATLLLPTLLLLIPFATLVSARPHLAGDFLRLPSETDNDDNVQGTRWAVLLAGSNGYWNYRHQADVCHAYQILRKGGLKEENIIVFMYDDIAFNGENPRPGVIINKP
DGGDVYEGVPKDYTGEDVTVGNFFAALLGNKSALTGGSGKVVDSGPDDHIFVYYTDHGGPGVLGMPAGPYLYADDLIEVLKKKHASGTYKNLVFYLEACESGSIFEG
LLPEDINIYATTASNAEESSWGTYCPGEYPSPPPEYSTCLGDLYSVAWMEDSDRHNLRTETLHQQYKLVKERTISGDSYYGSHVMQYGDVRLSSDVLFHYLGTDPAN
DNFTFVDENSLWSPSKPVNQRDADLIHFWDKFRKAPEGSLRKNAAQKQVLEAMSHRMHVDNSVKLIGKLLFGIEKGPEVLNAVRPAGSALVDDWHCLKTMVRTFETH
CGSLSQYGMKHMRSFANICNVGIKNEQMAEASAQACVSIPSNPWSSLQRGFSA

>gi:443501900 VPE Malus sikkimensis
MTRLASAVVLLFFASVLASAAGSRDLIGDILRLPSEASKFFGRGDDAPDQQDDGTVGTRWAVLIAGSNGYWNYRHQADICHAYQLLKKGGLKDENIVVFMYDDIAYN
EENPRQGVIINSPHGSDVYEGVPKDYTGEDVTVNNFFAAILGNKTALTGGSGKVVDSGPNDHIFIYYTDHGGPGILGMPTSPYIYANDLIEVLKKKHAAGTYKSLVF
YLEACESGSIFEGLLPEGLNIFATTASNAEESSWGTYCPGEYPSPPPEYDTCLGDLYSVAWMEDSDVHNLRSETLHQQYELVKTRTANDNSGFGSHVMQYGDVGLSK
NNLFVYMGTNPANDNHTFLGENSLRPSSKAVNQRDADLLHFWHKYRKAPEGSARKIQAQKDFVEAMSHRMHIDQTMKLIGKLLFGIEKGPQVLNAVRPAGQPLVDDW
DCLKTMVRSFETHCGSLSQYGMKHMRSLANICNAGMTQDQMAEASAQACVSAPSGRWSSLHRGFSA

>gi:357465673 VPE Medicago truncatula
MARFLFLIIATLIPIFSAATATAGDDFLRLPSQASRFFQSDDDNNEGTKWAILIAGSNGYWNYRHQSDVCHAYQVLRKGGLKEENIIVFMYDDIADNQENPRPGVII
NSPHGDDVYKGVPKDYTGDDVNVNNFFAALLGNKSALTGGSGKVVDSGPNDHIFIYYSDHGGPGVLGMPTGPFMYATDLIEVLKKKHASETYKSLVFYLEACESGSI
FEGLLPEGLNIYATTAANAEESSWGTYCPGENPSPPPEYETCLGDLYSVAWMEDSDIHNLQTETLHQQYELVKERTSNGNSIYGSHVMQFGDIGLSRDSLFLYLGSN
PANENFTFMGRNSLVPPSKTVNQRDADLIHFWDKFRKAPQGSPRKVAAQKQVLEAMSHRMHIDESIKLVGKLLFGMKKGPEVLASVRPAGQPVVDDWDCLKSLVRTF
ETYCGSLSQYGMKHMRSFANFCNAGIHSEQMAEASAQACINIPANPWSSLHGGFSA

>gi:48429177 VPE1 Phaseolus vulgaris
MATTTATTSLLALLLLFLVALVSAGRDLVGDFLRLPSDSGNGDNVHGTRWAILFAGSSGYWNYRHQADICHAYQLLRKGGLKDENIIVFMYDDIAFNSENPRRGVII
NSPNGDEVYKGVPKDYTGEDVTAHNFYAALLGDKSKLTGGSGKVVNSGPNDHIFIFYSDHGGPGVLGSPAGPYIYASDLNEVLKKKHASGTYKNLVFYLEACESGSI
FEGLLPEDINVYATTASNADESSWGTYCPGEDPSPPPEYSTCLGDLYSVAWMEDSDRHNLRTETLHQQYKLVKERTISGGLYYGSHVMQYGDVGLSKDILFHYLGTD
PANENLTFVDENSLWSSSKAVNQRDADLVHFWDKFRKAPEGSPKKNEARKQVLEVMSHRMHIDDSVELVGKLLFGIEKAPELLNAVRPAGSALVDDWDCLKTMVRTF
ETHCGSLSQYGMKHMRSFANMCNVGIKKEQMREASAQACVTIPANPWSSLQRGFSA

>gi:561009896 C13 peptidase Phaseolus vulgaris
MDGFPILFLLATLITLVSGGRDEILRLPSEASRFFQAPAADQNQEGTRWALLIAGSNGYWNYRHQSDVCHAYQLLRKGGLKEENIVVFMYDDIAFNEENPRPGVIIN
SPHGNDVYKGVPKDYVGEDVTVNNFFAAILGNKSALTGGSGKVIDSGPNDHIFIYYSDHGGPGVLGMPTSPYMYASDLIEVLKKKHASETYKSLVFYLEACESGSIF
EGLLPEGMNIYATTAANAEESSWGTYCPGEYPSPPPEYETCLGDLYSVAWMEDSDIHNLQTETLHQQFELVKQRTINGNSAYGSHVMQYGDIGLSKNNLSLYLGTNP
ANDNFAFREKNSLVPPSKAVNQRDADLVHLWDKYRKAPVGSSRKSVAQKQILEAMSHRMHIDDSMKLIGKLLFGIEEGPKLLNSVRPAGQPLVDDWDCLKTLVRTFE
THCGSLSQYGMKHMRSFANFCNAGIGKEQMAEASAQACVSIPATPWSSLRSGFSA

>Potri.006G232900 C13 peptidase Populus trichocarpa
MTRLIAGVIFLLISFCGIAVGVRDIVGDVLRLPSEASRFFRPGKFNDDNSDDDSSGTRWAILLAGSNGYWNYRHQADVCHAYQLLRQGGLKEENIIVFMYDDIADNP
ENPRPGVIINNPQGEDVYKGVPKDYTGPDVTVGNFFAAILGNKTALTGGSGKVIDSGPNDHIFIYYTDHGGPGVLGMPTNPYLYADDLIDVLKKKHASGTYKSLVFY
LEACESGSIFEGLLPQGLNIYATTASNAEESSWGTYCPGEYPSPPPEYETCLGDLYSVAWMEDSDIHNLRTETLHQQYELVKRRTSYDNSPYGSHVMQYGDVGLSKD
DLFQYMGTNPANDNYTFVEENSLRPHSKVVNQRDADLVHFWTKYRKAPEGSSRKVEAQKQFVEAMSHRMHIDHSIKLIGKLLFGIEKASEALNTVRPAGQPLVDDWV
CLKTLVRTFETHCGSISQYGMKHMRSLANLCNAGIVKEQMAEASAQACAFS

>gi:224141591 C13 peptidase Populus trichocarpa
MTGLATGAIFLLISLCGIAAAGRDTVGDVLRLPSEASRFFHNDDNSDDDSTGTRWAILLAGSNGYWNYRHQADVCHAYQLLRKGGLKEENIIVFMYDDIAYNSENPR
RGVIINSPQGEDVYKGVPKDYTGEDVTVGNFFAAILGNKTALTGGSGKVVDSGPNDHIFIYYTDHGGPGVLGMPTNPYLYADDLIDVLKKKHASGTYKSLVFYLEAC
ESGSIFEGLLPQGLNIYATTASNAEESSWGTYCPGENPSPPPEYETCLGDLYSVAWMEDSDIHNLQTETLHQQYELVKRRTSNDNSPYGSHVMQYGDVGLSKDNIFL
YMGTNPANDNFTFMDENLLRPRSKAVNQRDADLVHFWDKYRKAPEGSSRKVEAQKQFVEAMSHRMHIDHSIKLIGKLLFGIEKASEVLNAIRPAGQPLVDDWDCLKT
LVRTFETHCGSVSQYGMKHMRSLANLCNAGIGKEQMAEASAQACVSFPSGPWSTLHKGFSA

>gi:462419182 C13 pept Prunus persica
MTRLASAVVLLFLVSLSSFAAGSRDLIGDVLRLPSEASRFFGRGDDGPDEQDDGTVGTRWAVLIAGSNGYWNYRHQADICHAYQLLKKGGLKDENIVVFMYDDIAYN
EENPRPGVIINSPHGDDVYKGVPKDYTGDDVTVNNFFAAILGNKTALTGGTGKVVDSGPNDHIFIYYSDHGGPGVLGMPTSPYIYANDLIEVLKKKHAAGTYKSLVF
YLEACESGSIFEGLLPEGLNIFATTASNAEESSWGTYCPGEYPSPPPEYETCLGDLYSVAWMEDSDIHNLRSETLHQQYELVKTRTANDNSGFGSHVMQYGDVGLSK
ENLFVYMGTNPANDNYTFLGENSLRPSTKAVNQRDADLVHFWHKYRKAPEGSPRKIQAQKDFVEAMSHRMHMDQTMKLIGKLLFGIKKGPEVLNTVRPAGQPLVDDW
DCLKTMVRSFETYCGSLSQYGMKHMRSLANICNAGMTKEQMTEASAQACTSVPSSRWSSLHRGFSA

>gi:255550848 VPE Ricinus communis
MTIRLSTGIILILLLTLCGVVSSSRDIVGDVIRLPSEASRFFRPADGKNGDDDSAGTRWAILIAGSNGYWNYRHQADVCHAYQLLRKGGLKEENIIVFMYDDIAYNEE
NPRQGIIINNPHGEDVYKGVPKDYTGENVTVGNFFAAILGNRTALTGGRGKVVDSGPNDHIFVYYTDHGGPGVLGMPTNPYLYANDLIDVLKKKHASGTYKSLVFYL

EACESGSIFEGLLPEGLNIYATTASNAEESSWGTYCPGEYPSPPPEYETCLGDLYSIAWMEDSDVHNLQTETLHQQYELVKRRTSNGNSAYGSHVMQYGDVGLSREN
LFLYMGTNPANDNYTFVDENSLTPPSKAVNQRDADLVHFWDKYRKAPDGSARKDQAQKQFVEAMSHRMHIDHSVKLIGKLLFGLEKASEVLSTVRPAGQPLVDDWDC
LKKLVRTFETHCGSISQYGMKHMRSLANLCNAGIREEQMAEASAQACITFPSGPWSSLHKGFSA

>gi:590573851 VPE gamma Theobroma cacao
MTRLVSGVILLLLSLTGIVSAGRDITGDVLRLPSEASKFFRGSNDDEVEGTRWAVLIAGSNGYWNYRHQADVCHAYQLLKKGGLKDENIIVFMYDDIAFNEENPRPG
IIINSPHGDDVYEGVPKDYTGEDVTVNNLLAAILGNKTALTGGSGKVVDSGPNDHIFIYYTDHGGPGVLGMPTFPYLYADDLIEVLKKKHASGTYKSLVFYLEACES
GSIFEGLLPEGLNIYATTASNAEESSWGTYCPGEYPSPPPEYETCLGDLYSVAWMEDSDIHNLQTETLHQQYELVKRRTINGNSAYGSHVMQYGDIGLSKDIVFVYL
GTNPANDNFTFVDENSLQPPTKAVNQRDADLVHFWDKYRKAPDGSVRKLEAQKQFVEAMSHRMHIDNSMKLIGKLLFGIEKGPEVMKTVRPAGQPLVDDWKCLKKMV
RTFETHCGSLAQYGMKHMRSLANICNAGIQTEQMAEASAQACVSIPSGHWSSVQKGFSA

Brassicaceae alpha and gamma VPEs

>gi:297825691 VPE alpha Arabidopsis lyrata
MTTVAVTFLALFLYLVAAVSGDVIKLPSQASKFFHPTENDDDSTRWAVLVAGSSGYWNYRHQADVCHAYQLLKKGGVKEENIVVFMYDDIAKNEENPRPGVIINSPN
GEDVYNGVPKDYTGDDVNVDNLLAVILGNKTAVKGGSGKVVDSGPNDHIFIYYSDHGGPGVLGMPTSPYLYANDLNDVLKKKHASGTYKSLVFYLEACESGSIFEGL
LPEGLNIYATTASNAVESSWGTYCPGEDPSPPSEYETCLGDLYSVAWMEDSDIHNLQTETLHQQYELVKKRTAGSGKSFGSHVMEFGDIGLSKEKLVLYMGTNPANE
NFTFVNENSLRPPSRVTNQRDADLVHFWDKYRKAPEGSARKVEAQKQVLEAMSHRLHVDNSILLIGKLLFGLDSPAVLNNVRPSGTPLVDDWDCLKSLVRVFEMHCG
SLSQYGIKHMRSIANICNAGIQMGQMEEAAMQACPTIPASPWSSLERGFSA

>gi:297798670 VPE gamma Arabidopsis lyrata
MATTMTRVPVGAFLLVLLVSLVAVSTARSGPDDVIKLPSQASRFFRPAQDDDDSNAGTRWAVLVAGSSGYWNYRHQADICHAYQLLRKGGLKEENIVVFMYDDIANN
YENPRPGTLINSPHGKDVYQGVPKDYTGDDVNVDNLFAVILGDKTAVKGGSGKVVDSGPNDHIFIFYSDHGGPGVLGMPTSPYLYANDLNDVLKKKHASGTYKSLVF
YLEACESGSIFEGLLPEGLNIYATTASNAEESSWGTYCPGEEPSPPPEYETCLGDLYSVAWMEDSGMHNLQTETLHQQYELVKRRTAPVGYSYGSHVMQYGDVGLSK
DNLDLYMGTNPANDNFTFADANSLKPPSRVTNQRDADLVHFWEKYRKAPEGSARKTEAQKQVLEAMSHRLHVDNSVILVGKILFGISEGPEVLNKVRSAGQPLVDDW
NCLKNLVRAFERHCGSLSQYGIKHMRSFANICNAGIRTEQMEEAASQACTSIPPGPWSSLHRGFSA

>gi:15233996 VPE gamma Arabidopsis thaliana
MATTMTRVSVGVVLFVLLVSLVAVSAARSGPDDVIKLPSQASRFFRPAENDDDSNSGTRWAVLVAGSSGYWNYRHQADICHAYQLLRKGGLKEENIVVFMYDDIANN
YENPRPGTIINSPHGKDVYQGVPKDYTGDDVNVDNLFAVILGDKTAVKGGSGKVVDSGPNDHIFIFYSDHGGPGVLGMPTSPYLYANDLNDVLKKKHALGTYKSLVF
YLEACESGSIFEGLLPEGLNIYATTASNAEESSWGTYCPGEEPSPPPEYETCLGDLYSVAWMEDSGMHNLQTETLHQQYELVKRRTAPVGYSYGSHVMQYGDVGISK
DNLDLYMGTNPANDNFTFADANSLKPPSRVTNQRDADLVHFWEKYRKAPEGSARKTEAQKQVLEAMSHRLHIDNSVILVGKILFGISRGPEVLNKVRSAGQPLVDDW
NCLKNQVRAFERHCGSLSQYGIKHMRSFANICNAGIQMEQMEEAASQACTTLPTGPWSSLNRGFSA

>gi:15225226 VPE alpha Arabidopsis thaliana
MTTVVSFLALFLFLVAAVSGDVIKLPSLASKFFRPTENDDDSTKWAVLVAGSSGYWNYRHQADVCHAYQLLKKGGVKEENIVVFMYDDIAKNEENPRPGVIINSPNG
EDVYNGVPKDYTGDEVNVDNLLAVILGNKTALKGGSGKVVDSGPNDHIFIYYSDHGGPGVLGMPTSPNLYANDLNDVLKKKYASGTYKSLVFYLEACESGSIFEGLL
PEGLNIYATTASNAEESSWGTYCPGEDPSPPSEYETCLGDLYSVAWIEDSEKHNLQTETLHEQYELVKKRTAGSGKSYGSHVMEFGDIGLSKEKLVLFMGTNPADEN
FTFVNENSIRPPSRVTNQRDADLVHFWHKYQKAPEGSARKVEAQKQVLEAMSHRLHVDNSILLIGILLFGLEGHAVLNKVRPSGEPLVDDWDCLKSLVRAFERHCGS
LSQYGIKHMRSIANMCNAGIQMRQMEEAAMQACPTIPTSPWSSLDRGFSA

>gi:565477221 C13 peptidase Capsella rubella
MTRIVAVIVVVLFLSLVAAASDNFIRLPSEASKFFRPNNENDDSTRWAVLVAGSSGYWNYRHQADVCHAYQLLKKGGVKEENIVVFMYDDIANNEENPRPGVIINSP
NGEDVYNGVPKDYTGDEVNVNNFFAVLLGNKTALKGGSGKVVNSGPNDHIFIYYSDHGGPGVLGMPTSPYLYAKDLNDVLKKKHASGTYKSLVFYLEACESGSIFEG
LLPEGLNIYATTASNAEESSWGTYCPGEDPSPPSEYETCLGDLYSVAWMEDSDKHNLQTESLHQQYELVKKRTAGTGSSYGSHVLEFGDIGLSKEKLVLYMGTNPAN
ENFTFVDENSSLRLPSRVTNQRDADLVHFWDKYRKAPEGSARKVEAQKQVLEAMSHRLHVDNSVLLIGKLLFGLEGPAVLNKVRPSGRPLVDDWDCLKSMVRAFERH
CGSLSQYGIKHMRSIANICNAGIQMGLMEEAAKQACPSIPAGPWSSLHRGFSA

>gi:567217560 C13 peptidase Eutrema salsugineum
MTSVAVPLLVLLLSLIAVSAARQGPDDIIKLPSQASMFFRPADDDNDSSAGTRWAVLVAGSNGYWNYRHQADICHAYQLLRKGGVKEDNIVVFMYDDIANNEENPRR
GIIINSPHGKDVYQGVPKDYTGDDVTVDNLFAVILGNKTATKGGSGKVVDSGPNDHIFIFYSDHGGPGVLGMPTSPYLYANDLNDVLKKKHASGTYKSLVFYLEACE
SGSIFEGLLEEGLNIYATTASNAVESSWGTYCPGEDPSLPPEYETCLGDLYSVSWMEDSGMHNLQTETLRQQYELVKRRTAGVGSAYGSHVMQYGDVGLSKDKLDLY
MGTNPANDNFTFVDENSLTPPSRVTNQRDADLVHFWDKYRKAPEGSTRKTEAQKQVLEAMSHRLHVDNSVKLVGKLLFGISEGPEVLNKVRSAGQPLVDDWNCLKNL
VRAFERHCGSLSQYGIKHMRSFANICNAGIQMEQMEEASSQACTTIPPGPWSSLHRGFSA

>gi:312281859 C13 peptidase Thellungiella halophile
MTSVAVPLLVLLLSLIAVSAARQGPDDIIKLPSQASMFFRPADDDNDSSAGTRWAVLVAGSNGYWNYRHQADICHAYQLLRKGGVKEDNIVVFMYDDIANNEENPRR
GIIINSPHGKDVYQGVPKDYTGDDVTVDNLFAVILGNKTATKGGSGKVVDSGPNDHIFIFYSDHGGPGVLGMPTSPYLYANDLNDVLKKKHASGTYKSLVFYLEACE
SGSIFEGLLEEGLNIYATTASNAVESSWGTYCPGEDPSLPPEYETCLGDLYSVSWMEDSGMHNLQTETLRQQYELVKRRTAGVGSAYGSHVMQYGDVGLSKDKLDLY
MGTNPANDNFTFVDENSLTPPSRVTNQRDADLVHFWDKYRKAPEGSTRKTEAQKQVLEAMSHRLHVDNSVKLVGKLLFGISEGPEVLNKVRSAGQPLVDDWNCLKNL
VRAFERHCGSLSQYGIKHMRSFANICNAGIQMEQMEEASSQACTTIPPGP

Amborella VPEs beta

>262-1 Amborella trichopoda
MATSDRRSSLFYFLLLSLLLFRQRSFGVRVHEWVDSVIRMPSQKEGGEGVGTRWAVLVAGSSGFGNYRHQADVCHAYQLLLKGGLKEENIVVFMHDDIAYNEFNPKK
GIIINHPQGEDVYAGVPKDYTGKQVHTKNLYAVLLGNNSAVTGGSGKVINSKAEDRIFIYYSDHGGPGVLGMPNMPFLYANDLMEVLKKKHKSKGYKEMVIYVEACE
SGSIFEGLMTEDLNIYVTTASNAQESSWGTYCPGMDPPPPPEFMTCLGDLYSVAWMEDSETHNLKEETIQKQYVRVKSRTSNYNTYTAGSHVMEYGDKSIKSERLYL
YQGFDPANANLSDNSLPLQPNRMDVVNQRDADLLFLWQRYKRSTEGSEEKVAFRNEMTEKMAHREHLDKSVDLIGRLLFGWDKGSNVLGAKRPSGKALVDDWSCLKS
MVRAFEEKCGPLTQYGMKHMRAFANICNEGISLEVMSKACEEVCGRTYKHGILNVASHHGFSG

>262-2 Amborella trichopoda
MATSGLRRSLFYFLLLSLLLFRQRSFGVRVNEWVDSVIRMPSQKEGGEGVGTRWAVLVAGSSGFGNYRHQADVCHAYQLLLKGGLKEENIVVFMHDDIAYNEFNPKK
GIIINHPQGEDVYAGVPKDYTGKQVHTKNLYAVLLGNNSAVTGGSGKVINSKAEDRIFIYYSDHGGPGVLGMPNMPFLYANDLMEVLKKKHKSKGYKEMVIYVEACE
SGSIFEGLMTEDLNIYVTTASNAQESSWGTYCPGMDPPPPPEFMTCLGDLYSVAWMEDSETHNLKEETIQKQYVRVKSRTSNYNTYTAGSHVMEYGDKSIKSERLYL
YQGFDPANANLSDNSLPLQPNRMDVVNQRDADLLFLWQRYERSTEGSEEKVAIRNEMTEKMAHREHLDKSVDLIGRLLFGWDKGSNVLGAKRPSGKALVDDWSCLKS
MVRAFEEKCGPLTQYGMKHMRAFANICNEGISLEVMSKACEEVCGRTYKHGILNVASHHGFSG

Angiosperm beta VPEs

>gi:672145835 VPE Phoenix dactylifera
MATVTCSAIGWIALLSALLLPIGHCAGRVAGGWDSVIRMPAEMGSDDGDEEIGTRWAVLVAGSFGYGNYRHQADVCHAYQLLKKGGLKEENIVVMMHDDIANNPLNP
RPGVIINHPDGEDVYAGVPKDYTGEQVTTKNLYAVILGNKSAVEGGSGKVVDSKPVDRIFIYYSDHGGPGVLGMPNMPFLYAADFIDVLKKKHASGSYREMVI
YVEACESGSIFEGLLPEGLNIYVTTASNAEESSWGTYCPGMDPAPPPEYITCLGDLYSVAWMEDSATHNLKEETIEKQYEVVKERTSNYNTYSAGSHVMEYGDKSFK
DDKLYLFQGFDPSNANLSGNGLPPTMPTGAINQRDADLLFLWKRYEQLDARSEKKTQVLKEITETMMHRLHLDNSINLIGKLIFGSENGPSILNAVRPSGQALVDDW
NCLKTMVQVFQSYCGPLTQHGMRHMRAFANLCNRGISNDAMVEACVNTCGSHISAKWSPSSREYSAF

>gi:743827934 VPE Elaeis guineensis
MATATSSAIGWIVLLSALVLPIGHSAFRVGGGWESVIRMPTESGGDDGEQEIGTRWAVLVAGSFGYGNYRHQADVCHAYQLLKKGGLKEENIVVMMHDDIANNPLNP
RPGVIINHPQGEDVYAGVPKDYTGEHVTTTNLYAVILGNKSAVEGGSGKVVDSKPNDRIFIYYSDHGGPGVLGMPNMPFLYAADFIDVLKKKHASGGYKEMVIYVEA
CESGSIFEGLMPEDLNIYVTTASNAEESSWGTYCPGMDPSPPPEFITCLGDLYSVAWMEDSETHNLKEETIQKQYEEVKERTSNYNTYNTGSHVMEYGDKSFKDDKL
YLFQGFDPSNANLSGNALAPTMPMEAINQRDADLLFLWKRYEQFDEGSKKKTEALREITETLVHRLHLDNSINLIGKLIFGSENGPSILNAVRPSGQALVDDWNCLK
TMVQVFQTYCGPLTQYGMRHMRAFANICNRGSSKDAMVEACLNACESHISAKWSSSSQEYNA

>gi:695070617 legumain Musa acuminata subsp. Malaccensis
MATSSSPAAARRALFSFAVMLLLQMSLSASRPSLTRAAARWDPIIRLPSSRLVPEPGEGGRQDEEEETGTKWALLVAGSSGYGNYRHQADVCHAYQLLRRGGLKEEN
IVVMMHDDIAHNPLNPRQGVIINHPQGQDVYAGVPKDYTKEQVTAKNLYAVLLGDRSAIEGGSGKVIDSKPDDRIFIYYSDHGGPGVLGMPNMPFLYAADFIEVLKM
KHASNGYKEMVIYVEACESGSIFEGLMPENLDIYVTTASNAVESSWGTYCPGMDPPPPREFTTCLGDLYSVAWMEDSETHNLKEETVGKQFEEVKMRTSNHDTYNTS
HVMEYGDKSVKSDVLSLYQGFEPAIANVTENALRLRMPMGVINQRDADLLFMWKMYEQSDERSSKKKEILEEITKTLMHRVHLDSSIDYIGNQIFGSEIGPSILRAV
RPSGQALVDDWECLKSMVRAFESHCGSLTQYGMKHMRAFANICNEGISKDVMEAACSRSCKSYDGAAAMWSPSHRGFSA

>gi:695022010 VPE Musa acuminata subsp. Malaccensis
MMYDDIANSPLNPRPGVIINHPQGHDVYAGVPKDYTGKQVTSKNLYAVLLGIKSAVTGGSGKVIDSKPNDRIFIYYSDHGGPGVLGMPNMPYLYAVDFIEVLKKKHA
MNSYKEMVIYVEACESGSIFEGLMPKDLNIYVTTASNAEESSWGTYCPGMDPPPPPEYITCLGDLYSVAWMEDSETHNLKEETVSKQYEAVKVRTSNYNTYSVGSHV
MEYGDKNIKPEKLYLYQGFDPANANITENGLSQRMQMGTINQRDADLLFLWKRYERLAESSEDKRRTVMEITETMMHRTHLDRSIDLIGKLIFGSNSGPAILRAVRP
YGQALVDDWDCLKSMVRSFESHCGSLTQYGMKHMRAFANICNRGISRDAIKEASASACGNYNSAMWSSSMRGYSA

>gi:356463708 VPE2 Aegilops speltoides
MAPRWCFALLLLLCAAAGAGADASKGKWDPVIRMPGEEEPATGDESSEEGEDGVGTRWAVLVAGSSGYGNYRHQADICHAYQILRKGGVKEENIVVFMYDDIANNPL
NPRPGVIINHPEGEDVYAGVPKDYTGEAVTAKNFYAVLLGNNTAVTGGSKKVIDSKPNDHIFIYYSDHGGPGVLGMPNLPYLYAADFIKVLQEKHASNTYAKMVIYV
EACESGSIFEGLMPADLNIYVTTASNAEESSWGTYCPGMEPSPPSEYITCLGDLYSISWMEDSETNNLKEETIKKQYEVVKKRTSDMNSYSAGSHVMEYGDKTFKDE
KLYLYQGFNPANTNITNKLFLQAPKAAINQRDADLLFLWRRYELLHEKSKEKANVLREISETVAHRKHLDSSIDFIGKLLFGFENGPWELQAVRPSGKPLVDDWDCL
KRMVRIFESHCGSLTQYGMKHMRAFANICNNGVSGTTMNEASIGACGVQNSARWSTLIQGYSA

>gi:356463714 VPE2 Aegilops tauschii
MAPRWCFALLLLLCAAAGADASKGKWDPVIRMPGEEEPATGDDSSEEGEDGVGTRWAVLVAGSSGYGNYRHQADICHAYQILRKGGVKEENIVVFMYDDIANNPLNP
RPGVIINHPEGEDVYAGVPKDYTGEAVTAKNFYAVLLGNKTAVTGGSKKVIDSKPNDHIFIYYSDHGGPGVLGMPNLPYLYAADFIKVLQEKHASNTYAKMVIYVEA
CESGSIFEGLMPADLNIYVTTASNAEESSWGTYCPGMEPSPPSEYITCLGDLYSISWMEDSETNNLKEETIKKQYEVVKKRTSDMNSYSAGSHVMEYGDMTFKDEKL
YLYQGFNPANTNITNKLFLQAPKAAINQRDADLLFLWRRYELLHGKSKEKANVLTEIGETVAHRKHLDNSIDFIGKLLFGFENGPSELQAVRPSGKPLVDDWDCLKR
MVRIFESHCGSLTQYGMKHMRAFANICNNGVSGTTMKEASINTCGGHNSARLSTLIQGYSA

>gi:357136769 VPE beta Brachypodium distachyon
MAAWWCFALLLVLCAPAGADVSKGKWEPLIRMPGEKEPATARGFEGPEEEDGVGTRWAVLIAGSSGYGNYRHQADICHAYQVLRKGGLKEENIVVFMYDDIANSALN
PRPGVIINHPQGEDVYAGVPKDYTGEQVTAKNLYAVLLGNKTAVTGGSKKVIDSQPKDHIFIYYSDHGGPGVLGMPNLPYLYAGDFIKILQQKHASNTYAKMVIYVE
ACESGSIFEGLMPADLNIYVTTASNAEESSWGTYCPGMEPSPPSEYITCLGDLYSVSWMEDSENHNLKEETIKKQYEVVKRRTSDLNSYSAGSHVMEYGDKTFKDEK
LYLYQGFNPANANITNKLFWQAPRAAINQRDADLLFLWRRYEMLHEKSKEKVKVLREISETVMHRKHLDNSVDLIGQLLFGFENGPSVLQAVRPSGKPLVDDWDCLK
RMVRIFESHCGPLTQYGMKHMRAFANICNNGIPGSTMKEGSISACGSRNIARWSPLIQGYSA

>gi:6630462 VPE beta Arabidopsis thaliana
MAKSCYFRPALLLLLVLLVHAESRGRFEPKILMPTEEANPADQDEDGVGTRWAVLVAGSSGYGNYRHQADVCHAYQILRKGGLKEENIVVLMYDDIANHPLNPRPGT
LINHPDGDDVYAGVPKALHNNYSDSDCRDICYGKPNFMCGPFIGIAPRFLIATICSVIYVLKYLFQDYTGSSVTAANFYAVLLGDQKAVKGGSGKVIASKPNDHIFV
YYADHGGPGVLGMPNTPHIYAADFIETLKKKHASGTYKEMVIYVEACESGSIFEGIMPKDLNIYVTTASNAQESSYGTYCPGMNPSPPSEYITCLGDLYSVAWMEDS
ETHNLKKETIKQQYHTVKMRTSNYNTYSGGSHVMEYGNNSIKSEKLYLYQGFDPATVNLPLNELPVKSKIGVVNQRDADLLFLWHMYRTSEDGSRKKDDTLKELTET
TRHRKHLDASVELIATILFGPTMNVLNLVREPGLPLVDDWELKSMVRVFEEHCGSLTQYGMKHMRAFANVCNNGVSKELMEEASTAACGGYSEARYTVHPSILGYSA

>gi:1346432 Legumain Canavalia ensiformis
MVMMLVMLSLHGTAARLNRREWDSVIQLPTEPVDDEVGTRWAVLVAGSNGYGNYRHQADVCHAYQLLIKGGVKEENIVVFMYDDIAYNAMNPRPGVIINHPQGPDVY
AGVPKDYTGEDVTPENLYAVILGDKSKVKGGSGKVINSNPEDRIFIFYSDHGGPGVLGMPNAPFVYAMDFIDVLKKKHASGGYKEMVIYIEACESGSIFEGIMPKDL
NIYVTTASNAQENSFGTYCPGMNPPPPEEYVTCLGDLYSVSWMEDSETHNLKRETVQQQYQSVRKRTSNSNSYRFGSHVMQYGDTNITAEKLYLYHGFDPATVNFPP
HNGNLEAKMEVVNQRDAELLFMWQMYQRSNHQPEKKTHILEQITETVKHRNHLDGSVELIGVLLYGPGKSSSVLHSVRAPGLPLVDDWTCLKSMVRVFETHCGSLTQ
YGMKHMRAFGNVCNSGVSKASMEEACKAACGGYDAGLLYPSNTGYSA

>gi:565489031 C13 peptidase Capsella rubella
FSLVSKKASLSLSFLFLSVPTMAKSYYFRPALLLLLLLLLVRAESRGWFEPKILMPTEEAKPADQDEDEDGVGTRWAVLVAGSSGYGNYRHQADVCHAYQILRKGGL
KEENIVVMMYDDIANHQLNPRPGTLINHPNGEDVYAGVPKDYTGSNVTAANFYAVLLGDQKAVKGGSGKVIASKPNDHIFVYYADHGGPGVLGMPNTPHIYAADFIE
TLKKKHASGTYKEMVIYVEACESGSIFEGIMPKDLNIYVTTASNAQESSYGTYCPGMNPSPPSEYITCLGDLYSVAWMEDSENHNLKKETIKQQYQTVKMRTSNYNT
YSAGSHVMEYGNDSIKAEKLYLYQGFDPATVSLPPNELPVKSQVGVVNQRDADLLFLWHMYRRSEDGSRKKDDTLKELTETTRHRKHLDASVELIDTILFGPAMNVL
NSIREPGLPLVDDWECLKSTVRVFETHCGSLTQYGMKHMRAFANVCNNGISKELMEEASTAACGGYIEARYMLHPSILDYSA

>gi:502175795 VPE Cicer arietinum
MAVDSKKMCSKNVLSSWPLILLLLSFHGTAARLNRFEWDSVIRLPTEPVDADSDEGGTRWAVLVAGSSGYGNYRHQADVCHAYQLLVKGGVKEENIVVFMYDDIAQN
ELNPRPGVIINHPQGPDVYAGVPKDYTGDSVTAENLYAVILGDKSKVKGGSGKVINSKAEDRIFIYYSDHGGPGVLGMPNMPYLYAMDFINVLKKKHASRGYKKMVI
YVEACESGSIFEGIMPNNLNIYVTTASNAQENSWGTYCPGLDPAPPPEFITCLGDLYSVAWMEDSETHNLKRETVKEQYKSVKERTSNSNNYALGSHVMQYGDTNIT
DEKLYLYHGFNPATVNFPPHNGRLETKMEVVNQRDAELFFMWQLYKRLDHEAEKKRDILEKIAETVKHRNHLDGSVELVGVLLFGPEKGSSVLQSVRAPGLPLVDDW
ECLKSRVRVFETHCGSLTQYGMKHMRSFANICNNDISETSFEEACLKACGGYDVGLLHPSNGGYSA

>gi:567895782 C13 peptidase Citrus clementine
MLFKYDFLRSYHLTLPLFVLFLDCFFITNFLNFFHALSMATAHHRSVFFLLVLVLVLFTGGVQAARFNRRDSAILLPSEKQEPPAEEGGEPVGTRWAVLVAGSSGYA
NYRHQADVCHAYQLLRKGGLKEEHIVVFMYDDIAMHELNPRPGVIINHPQGENLYDGVPKDYTGEHVTAQNLYAVLLGDRKAVKGGSGKVVNSKANDRIFIFYSDHG
GPGVLGMPNMPYVYAMEFIDVLKKKHAAKSYKGMVIYVEACESGSIFEGVMPKDLDIYVTTASNAQESSFGTYCPGMDPSPPPEYITCLGDLYSVAWMEDSETHNLK
RETISQQYQAVKERTSNFNNYNSGSHVMEYGNTSVKSEKLYLYQGFDPASTNFPPNKLQPDQMGVVNQRDADLLFMWHMYKNAAEGSEKKSEMLKQITETMRHRKHL
DASIDMIGVILFGPDKGSRILNSVGARGLPLVDDWQCLKSMVRVFETHCGSLTQYGMKHMRAFANICNSGVSQALMEETSEAACSGNELRQWHPAIRGYSA

>gi:568846845 VPE Citrus sinensis
MLFKYDFLHSYHLTLSVFVLFLDCFFITNFLNFFHALSMATAHHRSVFFLLVLVLVLFTGGVQAARFNQRDSAILLPSEKQEPPAEEGGEPVGTRWAVLVAGSSGYA
NYRHQADVCHAYQLLRKGGLKEEHIVVFMYDDIAMHELNPRPGVIINHPQGENLYDGVPKDYTGEHVTAQNLYAVLLGDRKAVKGGSGKVVNSKANERIFIFYSDHG
GPGVLGMPNMPYVYAMEFIDVLKKKHAAKSYKEMVIYVEACESGSIFEGVMPKDLDIYVTTASNAQESSFGTYCPGMDPSPPPEYITCLGDLYSVAWMEDSETHNLK
RETINQQYQAVKERTSNFNNYNSGSHVMEYGNTSVKSEKLYLYQGFDPASANFPPNKLLPDQMGVVNQRDADLLFMWHMYKNAAEGSEKKSEMLKQITETMRHRKHL
DASIDMIGVILFGPDKGSRILNSVRARGLPLVDDWQCLKSMVRVFETHCGSLTQYGMKHMRAFANICNSGVSQALMEETSEAACSGNELRQWHLAIRGYSA

>gi:449459758 VPE Cucumis sativus
MASPSTSNLVLFLLLFLAYGCADASPWDRWERTIRMPTEKEEMGGAGDRKVGTRWAVLIAGSSGFGNYRHQADICHAYQLLKKGGLKDENIVVFMYDDIATNVLNPR
PGIIINHPQGEDVYAGVPKDYTGEHVTAQNLYAVLLGNRTAVDGGSGKVVDSKPNDRIFVYYSDHGGPGVLGMPNLPFVYAMDFIEVLKKKHAAKGYKEMVIYVEAC
ESGSIFEGILPKDLNIYVTTASNAQESSFGTYCPGMEPAPPPEYMTCLGDLYSVAWMEDSETHNLKRETIDQQYRTVKERTSNPNNLNTGSHVMEYGNSSIKAERLY
LYQGFDPASVNLPPYNGRYEMKSMDAINQRDADIFFLWQMYRKFEDGTNERAQVLEEIRETVTHRTHLDGSIRMIGFLLFGPEKGSNILDDVRASGLPLVDDWECLK
SMVRVLESYCGSLTQYGMKHMRAIANICNRGVSKASMREASMVACNGGSYGLWHPSNRGYSA

>gi:567127595 C13 peptidase Eutrema salsugineum
MAMSFCFRSALLLLLLLLLVRTESRGRFEPKILMPTEKTKPVEQEEAAIGTRWAVLVAGSSGYGNYRHQADVCHAYQILRKGGLKEENIVVLMYDDIANHPLNPRPGT
LINHPDGEDVYAGVPKDYTGHDVTAANFYAVLLGDKKAVKGGSGKVIASKPNDHIFVYYSDHGGPGVLGMPNTPHLYAADFIETLKKKHAAGTYREMVIYVEACESG
SVFEGIMPKDLNIYVTTASNAQESSYGTYCPGMNPSPPSEYITCLGDLYSVAWMEDSETHNLKKETIKQQYQTVKMRTSNYNSYSEGSHVMEYGNNTIKAEKLYLYQ
GFDPATVNLPPNELPVKSPVGVVNQRDADLLFLWHMYRTSEDGSRKDEILKEITDQMRHRKHLDASVELIGMILFGPTTNVLNSVREPGLPLVDDWECLKSMVRVFE
THCGSLTQYGMKHMRAFANVCNNGVSKELMEKASAAACGGYESRFMSHPSIVGYSA

>gi:470125355 VPE Fragaria vesca
MAARLSKLLLLFVALVIQTTIGGGGRAAARLDFWDSAAIRLPSEKDKTEDAVEQSGTTWAVLVAGSNGYGNYRHQADVCHAYQILKKGGLKEENIVVFMYDDIAMHE
MNPRKGIIINHPQGQDVYAGVPKDYTGEHVNAANLYAVLLGDKKAVKGGTGKVVASKPNDRIFLFYSDHGGPGVLGMPNMPFLYAMDFINVLKKKHASGSYKEMVIY
VEACESGSIFEGIMPSDLNIYVATASNAEENSFGTYCPGMNPPPPPEYITCLGDLYSVAWMEDSERHNLKKETIKEQYQTVKERTSNFNNYNIGSHVMQYGSKNLTE
EKLYLYLGFDPASVNFPPNNGQLEQHMEVVNQRDAEIFFMWQLYKRSEHGSEKKREILKQIRDTMNHRNHIDGSIKFIGTFLYGPKNGATVLNSVRPLGLPLVDDWE
CLKSMVRVFETHCGSLTQYGMKHMRAFANICNSGVSQAQMEEASWAACDGHDLGHLHPSNKGYSA

>gi:527209183 VPE Genlisea aurea

MASLYFKVIVVVLLAAVALDSVDGRRWDPIIRWPFHRRGTPENGTRWAVLVAGSNGFGNYRHQADVCHAYQILKRGGLKEENIVVFMYDDIADSEMNPRKGTIINHP
NGRDVYEGVPKDYTGENVTAANFYAVIQGNKTGVKGGSGKVVDSKPNDHVFIYYSDHGGPGVLGMPVMPFVYGKDLIEALKKKHASGTYEEMMIYVESCESGSVFEG
IMPTDLNVYVTTASNAEESSWGTYCPGMDPAPPPEYMTCLGDLYSVAWMEDSETHNLKKETVKEQYERVKERTSNFNSYNAGSHVMEYGNKSIKAEKLCLYQGFDPK
AETLLGSNGIRRRPLQVVNQRDADLLFMWERYKKLKDNSEEKSEHLKKSIIDAIAYRKHLDVSVETIGFILFGPANDLKSTRSQGLPLVDDWDCLKSMVQVFEERCG
SLTQYGMKHMRAFANICNNGVTTEDMAEASAVACSRSFGVYSE

>gi571453325 C13 peptidase Glycine max
MAVDRSLTRCCSLVLWSWMLLRMMMAQGAAARANRKEWDSVIKLPAEPVDADSDHEVGTRWAVLVAGSNGYGNYRHQADVCHAYQLLIKGGLKEENIVVFMYDDIAT
DELNPRPGVIINHPEGQDVYAGVPKDYTGENVTAQNLFAVILGDKNKVKGGSGKVINSKPEDRIFIYYSDHGGPGVLGMPNMPYLYAMDFIEVLKKKHASGGYKKMI
YVEACESGSMFEGIMPKDLQIYVTTASNAQENSWGTYCPGMDPSPPPEYITCLGDLYSVAWMEDSETHNLKRESVKQQYKSVKQRTSNFNNYAMGSHVMQYGDTNIT
AEKLYLYQGFDPAAVNFPPQNGRLETKMEVVNQRDAELFFMWQMYQRSNHQPEKKTDILKQIAETVKHRKHIDGSVELIGVLLYGPGKGSSVLQSMRAPGLALVDDW
TCLKSMVRVFETHCGTLTQYGMKHMRAFANICNSGVSEASMEEVCVAACEGYDSGLLHPSNKGYSA

>gi:351720847 VPE Glycine max
MALDRSIISKTTWYSVVLWMMVVLVRVHGAAARPNRKEWDSVIKLPTEPVDADSDEVGTRWAVLVAGSNGYGNYRHQADVCHAYQLLIKGGLKEENIVVFMYDDIAT
NELNPRHGVIINHPEGEDLYAGVPKDYTGDNVTTENLFAVILGDKSKLKGGSGKVINSKPEDRIFIYYSDHGGPGILGMPNMPYLYAMDFIDVLKKKHASGSYKEMV
IYVEACESGSVFEGIMPKDLNIYVTTASNAQENSWGTYCPGMDPSPPPEYITCLGDLYSVAWMEDSEAHNLKRESVKQQYKSVKQRTSNFNNYAMGSHVMQYGDTNI
TAEKLYLYQGFDPATVNFPPQNGRLETKMEVVNQRDAELFLLWQMYQRSNHQSENKTDILKQIAETVKHRKHIDGSVELIGVLLYGPGKGSSVLQSVRAPGSSLVDD
WTCLKSMVRVFETHCGTLTQYGMKHMRAFANICNSGVSEASMEEACLAACEGYNAGLFHPSNRGYSA

>gi:194352740 Legumain Hordeum vulgare
MAAWWVCGVLPLLEVAAAAAEPLIRLPTESGHAPATVPAPAPGPSAPAEEVTKWAVLVAGSSGYENYRHQADVCHAYQILKKGGLKDENIVVFMYDDIANSPENPRR
GVVINHPKGKDVYHGVPKDYTGDQVTAKNFYAVLLGNKTAVTGGSRKVINSKPEDHIFIYYTDHGGAGSLGMPNVPFVYAGDFIKVLRQKHASKSYSKMVVYVEACE
SGSIFEGLMPRDHNIYVTTAANAEESSWAAYCPGMEIPPPSEYYTCLGDAYSVSWMEDSETHNLKKETIKQQYEVVKARTAPRNKSSIGSHVMEYGDKTFKDEMLFL
YQGFDPAKSSITNRLPLPILKGAINQRDADVLFMWKKYEQLNGGSEEKHRALRDIKETVLHRKHLDSSIDFIGKLVFGFDKGPSMLQAARGSGQPLVDDWDCLKRMV
RVFESQCGSLTQYGMKHMRAFANICNNGVSEAEMKEASISACGGYDMGRWNPLVLGHSA

>gi:194352736 Legumain Hordeum vulgare
MAPWWCFGLLLLLCALAGADASKGRWDPVIRMPGEEEPATARGDGGSQEEEEDGVGTRWAVLVAGSSGYGNYRHQADICHAYQILRKGGVKEENIVVFMYDDIAKNA
LNPRPGVIINHPEGEDVYAGVPKDYTGEAVTAKNFYAVLLGNKTAVTGGSKKVIDSKSNDHIFIYYSDHGGPGVLGMPNLPYLYAADFIKVLQEKHASNTYAKMVIY
VEACESGSIFEGLMPADLNIYVTTASNAEESSWGTYCPGMEPSPPSEYITCLGDLYSISWMEDSETNNLKEETIKKQYEVVKKRTSDMNSYSAGSHVMEYGDKTFKD
EKLYLYQGFNPANTNITNKLFWQARKAAINQRDADLLFLWRRYELLHEKSKEKVNVLREISETVTHRKHLDSSIDFIGKLLFGFENGPSMLETVRPSGIPLVDDWDC
LKRMVRIFESHCGSLTQYGMKHMRAFANICNNGISGTSMKEASISTCGGGHNSARLSTLIQGYSA

>gi313660970 VPE2d Hordeum vulgare
MNSRVTMAAWWVCGVLPLLEVAAAAAEPLIRLPTESGHAPATVPAPAPGPSAPAEEVTKWAVLVAGSSGYENYRHQADVCHAYQILKKGGLKDENIVVFMYDDIANS
PENPRRGVVINHPKGKDVYHGVPKDYTGDQVTAKNFYAVLLGNKTAVTGGSRKVINSKPEDHIFIYYTDHGGAGSLGMPNVPFVYAGDFIKVLRQKHASKSYSKMVV
YVEACESGSIFEGLMPQDHNIYVTTAANAEESSWAAYCPGMEIPPPSEYYTCLGDAYSVSWMEDSETHNLKKETIKQQYEVVKARTAPGNKSSIGSHVMEYGDKTFK
DEMLFLYQGFDPAKSSITNRLPLPILKGAINQRDADVLFMWKKYEQLNGGSEEKHRALRDIKETVLHRKHLDSSIDFIGKLVFGFDKGPSMLQAARGSGQPLVDDWD
CLKRMVRVFESQCGSLTQYGMKHMRAFANICNNGVPEAEMKEASINACDGYDMGRWNPLVLGHSA

>gi:313660968 VPE2c Hordeum vulgare
MNSRVAMRAWWVCGFLPLLAVAADRAKASWEPLIRLPTDKGAAAPAPAPAPAAAAPVAEEGVTKWAVLVAGSSGYGNYRHQADVCHAYQILRQGGLKEENIVVFMFD
DIAKNHLNPRPGVIINHPKGEDVYAGVPKDYTGGQVTAKNFFAVLLGNKTAVTGGSGKVINSKPKDHIFIYYADHGGPGVLGMPNTPYLYAGDFIRVLREKHASKSY
SKMIIYVEACESGSIFEGLLPEDLNIYVTTASNAVENSWGAYCPGMKSSPPTEYDTCIGDIYSVSWMEDSETHNLKKETLKQQYEVVKSRTSKSKEFDKGSHVMEYG
DKTFKDEKLFHYQGFNPANSNVANRLLLPDLEGAINQRDADILFMWKRYEKLNGGSEEKLGVLREIKETVAHRKHLDSSIDFIGKLVFGFENGPSVLEAARSSGQPL
VDDWDCLKRMVRVFESQCGSLTQYGMKHMRAFANICNNGVSEAQMREASISACGGYNLAKWSPLDLGHSA

>gi:357476961 VPE Medicago truncatula
MFSIVLSLWSWLLLLLTLDGLVARPNHLEWDPVIRLPGEVVDDAEVDEVGTRWAVLVAGSSGYGNYRHQADVCHAYQLLIKGGVKEENIVVFMYDDIANNELNPRPG
VIINHPQGPNVYVGVPKDYTGDNVTAENLYAVILGDKSKVKGGSGKVINSKSEDRIFIYYSDHGGPGVLGMPNMPYVYAMDFIDVLKKKHASGGYKKMVVYIEACES
GSMFEGVMPKDLNVYVTTASNAQESSWGTYCPGVEPAPPPEYITCLGDLYSVAWMEDSESHNLKRETVKQQYKSVKERTSNYNNYALGSHVMQYGDTNITDEKLYLY
QGFDPATVNLPPHNDKLESKMEVVNQRDAEILFMWEMYKRLDHQTEKKREILEKIAETVKHRNHLDGSVELIGVLLFGPTKGSSVLQAVRATGLPLVDDWECLKSRV
RLFETHCGSLTQYGMKHMRAFANICNSGISEDSMEKACMVACGGYKLELLHPSNRVYSA

>gi:217074670 C13 peptidase Medicago truncatula
MAVHSKNMFSIVLSLWSWLLLLLTLDGLVARPNHLEWDPVIRLPGEVVDDAEVDEVGTRWAVLVAGSSGYGNYRHQADVCHAYQLLIKGGVKEENIVVFMYDDIANN
ELNPRPGVIINHPQGPNVYVGVPKDYTGDNVTAENLYAVILGDKSKVKGGSGKVINSKSEDRIFIYYSDHGGPGVLGMPNMPYVYAMDFIDVLKKKHASGGYKKMVV
YIEACESGSMFEGVMPKDLNVYVTTASNAQESSWGTYCPGVEPAPPPEYITCLGDLYSVAWFLLFV

>gi:587864848 VPE Morus notabilis
MGDQYYHPVLRGKKNNSICAMLVIMILVVLSVENCRAARFERWESAIRMPTDKDEYSDQDKELSTRWAVLVAGSSGYGNYRHQADVCHAYQLLRKGGVKEENIVVFM
YDDIAMHGLNPRPGIIINHPQGDDVYAGVPKDYTGQYVTTENLYAVLLGDKKAVKGGSGKVVDSKPNDRIFLYYSDHGGPGVLGMPNMPFLYAMDFVEVLKKKHASG
TYKEMIIYVEACESGSIFEGIMPRDMNIYVTTASNAQENSWGTYCPGMEPSPPPEYITCLGDLYSVAWMEDSETHNLKRETIKQQYETVKKRTANANDFTAGSHVME
YGNKSIKGEKLYLYQGFDPATLNFPPNDPNKLDMRMEVVNQRDAELFFMWQMYKRTESRNKKREILNQIKEATRHRTHLDKSIELIGSLLYGPQKGSLVLNSVRSPG
QPLVDDWACLKSMVKSFESHCGSLTQYGMKHMRAFANICNSGVSVASMEEAALAVCSGHDVGQWHPSNGGFSA

>gi:40809676 Legumain Nicotiana tabacum
MGSFSFAVCVSLMLLVMVVAIPFELPKNGRRIGRLHRWWDPLIRSPVDRDDESEDKDGVRWAVLVAGSNGYGNYRHQADVCHAYQILKRGGLKDENIVVFMYDDIAK
SELNPRPGVIINHPNGSDVYAGVPKDYTGEHVTAANLYAVLLGDKSAVKGGSGKIVDSKPNDRIFLYYSDHGGPGVLGMPNMPFLYAKDFIEVLKKKHAAGTYKEMV
LYIEACESGSVFEGMMPEDLNIYVTTASNAEESSWGTYCPGMDPPPPPEYITCLGDLYSVAWMEDSESHNLKKETIKQQYEKVKERTSNFNNYNAGSHVMEYGSKEI
KPEKVYLYQGFDPATANLSANKIAFAHVEVVNQRDADLLFLWERYKELADNSLEKAKLRKEITDTMLHRKHLDGSVDAIGVFLFGPTKGSSVLNSVREPGLPLVDDW
DCLKSTVRLFELHCGSLTQYGMKHMRAFANICNNGVSRDAMEEAFMAACNERKREEYTAANRGFSA

>gi:573919988 VPE Oryza brachyantha
MAAQWCFALLLALSAAAEAGAKRMWEPVIRMPGEVVVEEEVVGGIGTRWAVLVAGSSGYGNYRHQADVCHAYQILRKGGLKEENIVVFMYDDIANNALNPRPGVIVN
HPQGEDVYAGVPKDYTGDEVNTKNFYAVLLGNKTAVTGGSRKVIDSKPNDHIFIFYSDHGGPGVLGMPNLPYLYAADFMKVLQEKHVSNTYAKMVIYVEACESGSIF
EGLMPEDLNVYVTTASNAEESSWGTYCPGMEPSPPAEYITCLGDLYSVSWMEDSETHNLKEESIKEQYEVVKKRTSDMNSYGAGSHVMEYGDKTFKGEKLYLYQGFD
PANAEVKNKLLLDDPKAAVNQRDADLLFLWRRYELLHEKSEEKLKVLREISEAVTHRKHLDSSVDFVGKLLFGFGNGPTVLQHVRPSGQPLVDDWDCLKRMVRIFES
HCGSLTQYGMKHMRAFANICNNGIPDAAMKDASIVACSSDNSARWSSLVQGYSA

>gi:26006020 C13 peptidase Oryza sativa Japonica
MAARWCFALLLALSAAAAGAGAKRTWEPVIRMPGEVVEEEVATVPRGSEGTEEEEKDGVGTRWAVLVAGSSGYGNYRHQADVCHAYQILRKGGLKEENIVVFMYDDI
ANNILNPRPGVIVNHPQGEDVYAGVPKDYTGDEVTAKNFYAVLLGNKTAVTGGSRKVIDSKPNDHIFIFYSDHGGPGVLGMPNLPYLYAADFMKVLQEKHASNTYAK
MVIYVEACESGSIFEGLMPEDLNIYVTTASNAEESSWGTYCPGMEPSPPSEYITCLGDLYSVSWMEDSETHNLKEESIKKQYEVVKKRTSDMNSYGAGSHVMEYGDR
TFKDDKLYLYQGFDPANAEVKNKLSWEGPKAAVNQRDADLLFLWRRYELLHDKSEEKLKALREISDTVMHRKLLDSSVDLVGKLLFGFGNGPSVLQAVRPSGQPLVD
DWDCLKRMVRIFESHCGPLTQYGMKHMRAFANICNNGISGASMKEASIATCSSHNSGRWSSLVQGYSA

>gi:38567871 C13 peptidase Oryza sativa Japonica
MAARCWVWGFVVALLAVAAAADGEEEEGKWEPLIRMPTEEGDDAEAAAPAPAPAAADYGGTRWAVLVAGSSGYGNYRHQADVCHAYQILQKGGVKEENIVVFMYDDI
AHNILNPRPGTIINHPKGGDVYAGVPKDYTGHQVTTENFFAVLLGNKTAVTGGSGKVIDSKPEDHIFIYYSDHGGPGVLGMPNLPYLYAGDFIKVLQKKHASNSYSK
MVIYVEACESGSIFEGLMPENLNIYVTTASNAVENSWGTYCPGEEPSPPPEYITCLGDMYSVAWMEDSETHNLKKETIEDQYELVKKRTSNANKLNEGSHVMEYGDK
TFKDEKLFLYQGFNPANGNITNELIWPVPKATVNQRDADLLFMWKRDNGVEFARVVSFMLIWLTSPYEQLNGVSEDKLRALREIEDTIAHRKHLDSSIDFIGKLVFG
FENGPLALEAARSSGQPLVDNWDCLKKMVRIFESQCGSLTQYGMKYMRAFANICNNGVSEAKMMEASINACGRYNSARWSPMTEGGHSA

>gi:561028978 C13 peptidase Phaseolus vulgaris
MAVHRSLLNKPTWCSVAFWWWMLVMVMRIQGTNGKEQDSVIKLPTQEVDAESDEVGTRWAVLVAGSNGYGNYRHQADVCHAYQLLIKGGVKEENIVVFMYDDIATHE
LNPRPGVIINNPQGPDVYAGVPKDYTGESVTSRNFFAVLLGDKSKVKGGSGKVINSKPEDRIFVYYSDHGGPGVLGMPNMPYLYAMDFIDVLKKKHASGGYKEMVIY

VEACESGSIFEGIMPKDLNIYVTTASNAQENSWGTYCPGMYPPPPPEYITCLGDLYSVAWMEDSESHNLKKESVEQQYQSVKQRTSNFEAYAMGSHVMQYGDANMTA
EKLYLYHGFDPATVNFPPHNGRLKSKMEVVNQRDAELLFMWQMYQRSNHLPEKKTDILKQIEEIVKHRKHLDGSVELIGVLLYGPEKASSVLRSVRTTGLPLVDDWT
CLKSMVRVYETHCGSLTQYGMKHMRAFANICNSGVSETSMEKACVAACGGYHAGLLHPSNTGYSA

>gi:566162122 C13 peptidase Populus trichocarpa
METHKPYFLSAIFVLVMLSFLHEQSSQAARLNPVEAGILMPTEKDGPEVDDDDGKEIGTRWAVLVAGSNGYGNYRHQADVCHAYQLLRKGGIKEENIVVFMYDDIAK
HEFNPRPGVIINHPQGDDVYAGVPKDYTGVQVTTENLYAVLLGNKSAVKGGSGKVVDSMPNDRIFLYYSDHGGPGVLGMPTMPFLYAMDFIEVLKKKHASGSYKEMV
MYIEACESGSIFEGIMPKDINIYVTTASNAEENSWGTYCPGMEPSPPPEYFTCLGDLYSVSWMEDSGKHNLRRETIEQQYHSVKERTSNYNTFTSGSHVMQYGNKSI
KGEKLYLYQGFNPASVNFPPNNVHIGGRMDVVNQRDAELVFLWQMYKRSEDGSEKKTQILNQIKETMRHRTQLDSSMELIGTLLFGRKKGSAILKSVREPGSPLVDD
WICLKSMVRRFETHCGSLTQYGMKHMRAFANICNGGVSQASMEEACIAACSGHEFGDLRPSDQGYSA

>Potri.001G119800 C13 peptidase Populus trichocarpa
METHKAYFLSAILVLAMLSFLHVQSVQAARLSPVEPRILMPTGKDEPEVDDDGEEIGSRWAVLVAGSSGYGNYRHQADVCHAYQLLRKGGIKEENMVVFMYDDIAMH
HLNPRPGVIINHPQGDDVYAGVPKDYTGEQVNTENLYAVLLGNKSAVKGGSGKVVDSKPNDRIFLYYSDHGGPGVLGMPNMPFLYAMDFIEVLKKKHASGSYKEMVM
YIEACESGSIFEGIMPKDLNIYYVTCLGDLYSVAWMEDSETHNLKKETIKQQYHSVKERTSNYNAFTSGSHVMQYGNESLKGEKLFLYQGFDPASVNFPPNNGHIGA
RMDVVNQRDAELVFLWQMYKRAEGGSEKKTQILNQIKETMRHRTHLDSSMELIGTLLLGPKKGSAILKSVREPDSPLVDDWRCLKSMVRLFETHCGSLTQYGMKHMR
AFANICNGGVSLASMEEACVAACSGHDAGELHPSNQGYST

>gi:462405686 C13 peptidase Prunus persica
MAVVQYQSRLILLLVLFVLLVGGGRVARLDLWDSTIRLPSDKDKPEDANHKEAGTRWAVLVAGSSGYGNYRHQADVCHAYQLLKKGGLKEENIVVFMYDDIANHEIN
PRPGVIINHPQGQDVYAGVPKDYTGKQVTAANLYAVLLGDKKAVKGGSGKVVDSKPNDRIFLYYSDHGGPGVLGMPNMPFLYAMDFIQVLKKKHASGSYKEMVIYVE
ACESGSIFEGIMPSDLNIYVTTASNAQENSFGTYCPGMDPSPPPEYITCLGDLYSVAWMEDSERHNLKKETIKQQYQNVKKRTSNSNNYDVGSHVMQYGSKNITVEK
LYLYQGFDPASLNFPPNNGQLEKPMEVVNQRDAELFFMWQMYKRSEHGSEKKTHILEQIKETMGHRTHLDASIEFIGTFLYGPGKGFSTLNSVRALGLPLVDDWECL
KSMVRVFETQCGLLTQYGMKHMRAFANICNSGVTQSEMEEACSAACNGHDPGQLHPSNKGYSA

>gi:255537021 VPE Ricinus communis
METHKSLLFFTNYVLFLVFTLSFLPIPGLLASRLNPFEPGILMPTEEAEPVQVDDDDQLGTRWAVLVAGSMGFGNYRHQADVCHAYQLLRKGGLKEENIIVFMYDDI
AKNELNPRPGVIINHPQGEDVYAGVPKDYTGEHVTAKNLYAVLLGDKSAVQGGSGKVVDSKPNDRIFLYYSDHGGPGVLGMPNLPYLYAMDFIEVLKKKHAAGGYKK
MVIYVEACESGSIFEGIMPKDVDIYVTTASNAQESSWGTYCPGMEPSPPPEFTTCLGDLYSVAWMEDSESHNLKKETVKQQYSSVKARTSNYNTYAAGSHVMQYGNQ
SIKADKLYLFQGFDPASVNFPPNNAHLNAPMEVVNQRDAELHFMWQLYKRSENGSEKKKEILQQIKDAIKHRSHLDSSMQLIGDLLFGPKKASAILKSVREPGSPLV
DDWGCLKSMVRVFETCCGSLTQYGMKHMRTFANICNAGVSHTSMEEACNAACSGHDAGQWHPTNQGYSA

>gi:9622221 C13 peptidase Sesamum indicum
MAFSCFAGRLMILVLCVVVALPFAAAGGGRRSGPWDPIIRWPLDRRETEDNATRWAVLVAGSNGFGNYRHQADVCHAYQILKKGGLRDENIIVFMYDDIAMNELNPR
KGVIINHPTGGDVYAGVPKDYTGEQVTAENLYAVILGDKSAIKGGSGKVVDSKPNDRIFIYYSDHGGPGVLGMPNMPYLYANDFIEVLKKKHASGTYKEMVIYVEAC
ESGSVFEGLMPDDLDIYVTTASNAEESSWGTYCPGMDPPPPPEYITCLGDLYSVAWMEDSESHNLKRETVEQQYQQVKERTSNFNTYNAGSHVMEYGNKSIKSEKLY
LYQGFDPATENMPPSENHLKPHMDVVNQRDADLLFLWERYKRLDGGAKKKSELFKLITDTMLHRKHMDDSIDIIGAFLFGPENGPSILKSVRDRGLPLADDWDCLKS
MVRLFEAHCGSLTQYGMKHTRAFANICNSRVSSADMEDACMAACRGHDFAGWSPLNRGYSA

>gi:514715547 VPE beta Setaria italica
MAVCRCLVVLVAALAAAAAGAGTEEGDWDPVIRMPGEEEPAAARGGEPLDEEEDDGVGTRWAVLVAGSSGYGNYRHQADICHAYQILLKGGLKEENIVVFMYDDVAN
SVLNPRQGVIINHPEGEDVYAGVPKDYTGEQVTAKNFYAVLLGNKDAVTGGSRKVINSKPNDHIFIYYSDHGGPGVLGMPNLPYIYAGDFMKVLREKHASNSYAKMV
IYVEACESGSIFEGLMPEDLNIYVTTASNAEESSWGTYCPGMIPSPPSEYITCLGDLYSVSWMEDSETHNLKEETIKEQYEAVKERTSDSNSYGAGSHVMEYGDKTF
KGEKLYLYQGFNPENADITYKLLWQGQKSVVNQRDADILFLWKRYELLNEKSKEKLEVLREITGTVTHRKHLDSSVDFIGKLLFGVENGPSTLGAVRSPGQPLVDDW
DCLKRMVRIFESHCGSLTQYGMKHMRAFANICNRGTPATAMKEASISACGSYNSARWSPLVQGYSA

>gi:514802965 VPE Setaria italica
MAAAAWLCGLLSLLAVAAAASVDGAEEEWEPLIRMPTEKGGNAAAAAPAAEEDEVGTRWAVLVAGSSGYGNYRHQADVCHAYQILLKGGVKEENIVVFMYDDIAHNI
LNPRPGVIINHPKGENVYPGVPKDYTGDQVTTENFFAVLLGNRSAITGGSKKVIDSKPNDHIFIYYSDHGGPGVLGMPNLPYLYAGDFIKVLKKKHASNSYSKMVIY
VEACESGSIFEGLMPQDLNIYVTTASNPVENSWGTYCPGMDPSPPPEYITCLGDLYSVSWMEDSQTHNLMKETIKDQYEVVKTRTSNLKKYKEGSHVMEYGDKTFTN
EKLFLYQGFDPANANAANTLLWPGPKGAVNQRDADLLFMWKRYEQLDGGSEEKLRALREIKETVQHRKHLDSSIDFIGRLVFGFENGPKMLEAVRASGQPLVDDWDC
LKRMVRIFEAQCGSLTQYGMKYMRAFANICNSGISEAKMRESSISACGGYNSARWSPMAQGHSA

>gi:460400421 VPE Solanum lycopersicum
MMGSCNFTVCVTLMLLVMVGAISIEPKIDSRRLGRPHRFWDPLIRSPVDRDDDDETEEGGGGVRWAVLVAGSNGYGNYRHQADVCHAYQILKRGGLNDENIVVFMYD
DIAKSELNPRPGVIINHPNGSDVYAGVPKDYTGEHVTAANLYAVLLGDKSAVKGGSGKVVNSGPNDRIFLYYSDHGGPGVLGMPNMPYLYGKDLIEVLKKKYAARTY
KEMVLYIEACESGSVFEGLMPENLNIYVMTASNAEESSWGTYCPGMDPPPPSEYITCLGDLYSVAWMEDSESHNLKKETIKQQYEKVKERTSNSNNYNAGSHVMEYG
SKEIKPEKVYLYQGFDPATVNLPANKIDFARLEVVNQRDADLLFLWERYKKLEDNSLEKAKLRKEITETMLHRQHLDGSIDAVGVFLFGPIKGGSVLSSVRKPGLPL
VDDWECLKSTVRLFEAHCGSLTQYGMKHMRAFANICNNGISSDAMEDAFMAACNGHSLEEYTTANRGFSA

>gi:565378091 VPE beta Solanum tuberosum
MGSCNFTVCVTLMLLVMVGAISFEPKIDTRIGRAHRLWDPLIRSPVDRDDDEMEEDGGGVRWAVLVAGSNGYGNYRHQADVCHAYQILKRGGLKDENIVVFMYDDIA
KSELNPRPGVIINHPNGSDVYAGVPKDYTGEHVTAANLYAVLLGDKSAVKGGSGKVVNSGPNDRIFLYYSDHGGPGVLGMPNMPYLYGKDLIEVLKKKYAAGTYKEM
VLYIEACESGSVFEGLMPENLNIYVTTASNAEESSWGTYCPGMDPPPPSEYITCLGDLYSVAWMEDSESHNLKKETIKQQYEKVKERTSNSNNYNAGSHVMEYGSKE
IKPEKVYLYQGFDPATVNLPANKIDFARLEVVNQRDADLLFLWERYKKLEDNSLEKAKLRKEITETLQHRQHLDGSIDAVGVFLFGPIKGGSVLSSVRKPGLPLVDD
WECLKSTVRLFEAHCGSLAQYGMKHMRAFANICNNGISRDAMEEAFMAACNGHTEEYSAANRGFSA

>gi:242063120 C13 peptidase Sorghum bicolor
MAARWCLVLLVLVLAAAAAGAEKGEWDPVIRMPGEEEPAASSHSHSGEGFEGEEDVAVGTRWAVLVAGSSGYGNYRHQADICHAYQILRKGGIKEENIVVFMYDDVA
TSALNPRQGVIINHPQGEDVYAGVPKDYTGDQVTAKNFFAVLLGNKTAVTGGSRKVINSKPDDHIFICYSDHGGPGVLGMPNLPYLYAGDFMKVLREKHASNSYAKM
VIYIEACESGSIFEGLMPEDLNIYVTTASNAEESSWGTYCPGMEPSPPSEYITCLGDLYSVSWMEDSETHNLKEETIKEQYEVVKERTSDSNSYGAGSHVMEYGDKT
FKGEKLYLYQGFDPANANVTNKLLRPGLEAVVNQRDADILFLWKRYELLHEKSEEKQEVLREITGTVRHRKHLDSSIDFIGKLLFGIEKGPFTLQAVRPSGQPLVDD
WDCLKQMVRIFESHCGSLTQYGMKHMRAFANICNSGTPGASMKQASMGACGSYNSARWSPLVQGYSA

>gi:242076602 C13 peptidase Sorghum bicolor
MAAAAWLCGLLWLLAHAAAVASAADGADGGWEPLIRMPTGKGGDAAARAVEEDDEVGTRWAVLVAGSSGYGNYRHQADVCHAYQILRKGGVKEENIVVFMYDDIAHN
ILNPRPGVIINHPKGENVYNGVPKDYTGDQVTTENFFAVLLGNKSAITGGSKKVIDSKPNDHIFIYYSDHGGPGVLGMPNLPYLYAGDFIKVLKKKHACNSYSKMVI
YVEACESGSIFEGLMPEDLNIYVTTASNPVENSWGTYCPGMEPSPPPEYITCLGDLYSVSWMEDSQTHNLKKETIKDQYEVVKTRTSNSNKYKEGSHVMEYGDKTFK
DEKLFLYQGFDPANANIANMLLWPGPKGAVNQRDADLLFMWKRYEQLNGESVEKLRALIEIKETVQHRKHLDSSIDFIGRLLFGFEKGPSMLEAVRASGLPLVDDWD
CLKRMVRIFESQCGSLTQYGMKYMRAFANICNSGISEMKMRESSISACSSYNSARWSPMAQGHSA

>gi:590677122 VPE beta Theobroma cacao
METATLSLTRNSLPDSILHPCFPFKHQFPLPFVSFSSYLFTAFLNWIHYLLVISMAKQDSVFIKYLSLLLVLLLLFEAGRAARLNQWESGIRLSTDIDEPQDVDDQQ
LGTRWAVLVAGSSGYANYRHQADVCHAYQLLRKGGLKEENIVVFMYDDIAMHKLNPRPEVIINHPKGDDVYAGVPKDYTGVHVTAANLYAVLLGNNSALSGGSGKVV
DSKPNDRIFLFYSDHGGPGVLGMPNLPFLYAMDFLDVLKKKHAAGSYKEMVIYVEACESGSVFEGIMPKDLNIYVTTASNAQESSWGTYCPGMEPSPPPEYTTCLGD
LYSVAWMEDSETHNLKRETVKQQYETVRERTSNFNSYTLGGSHVMEYGNASIKAEKLCSYQGFDPSSENFPPNELTHMEAVNQRDADILFLWHMYKNSEDGSKKTEI
LRQITETIRHRIHLDGSIDLIGTLLYGPAKGSGILNSVREPGLPLVDDWQCLKSMVRLFETHCGLLTQYGMKHMRAFANLCNSGVSQSLMEQACVAACSGHDTRQWH
PSNQGYSA

>gi:356463700 VPE1 Triticum monococcum
MAPRWCFALLLLLCAAARPGADASKGKWDPVIRMPGEEEPATGDESSEEGEDGVGTRWAVLVAGSSGYGNYRHQADICHAYQILRKGGVKEENIVVFMYDDIANNPL
NPRPGVIINHPEGEDVYAGVPKDYTGEEVTAKNFYAVLLGNKTAVTGGSKKVIDSKPNDHIFIYYSDHGGPGVLGMPNLPYLYAADFIKVLQEKHASNTYAKMVIYV
EACESGSIFEGLMPADLNIYVTTASNAEESSWGTYCPGMEPSPPSEYITCLGDLYSISWMEDSETNNLKEETIKKQYEVVKKRTSDMNSYSAGSHVMEYGDKTFKDE
KLYLYQGFNPANTNITNMLLLQAPKAAINQRDADLLFLWRRYELLHEKSKEKGNVLREISETVTHRKHLDSSIDFIGKLLFGFENGPSVLQAVRPSGKPLVDDWDCL
KRMVRIFESHCGSLTQYGMKHMRAFANICNNGISGTTMKEASIGACGVQNSARWSSLIQGYSA

>gi:474124510 VPE Triticum urartu

MAAWWAPPPAPGPAASAPEERVTKWAVLVAGSSGYENYRHQADVCHAYQILKKGGLKDENIVVFMYDDIANSPENPRRGVVINHPKGKDVYHGVPKDYTGEHVTAKN
LYAVLLGNKTAVTGGSRKVINSKPNDHIFIYYTDHGGAGSLGMPNVPFVYAGDFIKVLRQKHASKSYSKMIIYVEACESGSIFEGLMPQDHNIYVTTAANAEESSWA
AYCPGMEVPPPSEYKTCLGDAYSVSWMEDSETHNLKKESIKQQYEVVKARTAPPNESSIGSHVMEYGDKTFKGDMLFLYQGFDPAKSYSSYFRQRLPSLKGAINQRD
ADILFMWKKYEQLNGGSEEKQRALTEIKETVLHRKHLDSSIDFIGKLVFGFEKGPSVLDAARGSGQPLVDDWDCLKTMVRIFESQCGSLTQYGMKHMRAFANICNNG
VSEAEMKEASISACDDYDMGKWSPLVRGHSA

>gi:2414681 C13 peptidase Vicia narbonensis
MFSDVLASWLVLLLLSSLHGSVAARPNRLEWEPVIRLPGEPVDADVEDEMGTRWAVLVAGSNGYGNYRHQADVCHAYQLLIKGGVKEENIVVFMYDDIAYNEMNPRP
GVIINHPQGPNVYDGVPKDYNGDFVTAENFYAVILGDKSKVRGGSGKVINSKAEDRIFIYCSDHGGPGVLGMPNMPYVYAMDFIDVLKKKHASGGYKKMVIYVEACE
SGSIFEGIMPKDINVYVTTASNAQENSWGTYCPGVDPAPPPEYITCLGDLYSVAWMEDSETHNLKRETVKQQYMSVRERTSNYKNYPLGSHVMQYGDTNITDEKLYL
YHGFDPADGEPSSNNDILEAKMEVVNQRDAEILFMWHMYQRLDHQTEKKKDTLEKISETVKHRNHLDGSVELIGVLLFGPTKGSSVLQSVRASGLPLVDDWECLKSR
VRVFETHCGSLTQYGMKHMRAFANICNSGISEDSMEEACMAACGGYDVGLLHPSNKGYSA

>gi:3452551 C13 peptidase Vicia sativa
MSVYRCHSVFLSKNMFSDVLASWLVLLLFLSSLHGSVARPNRLEWEPVIRLPGEPVDADVEDEIGTRWAVLVAGSNGYGNYRHQADVCHAYQLLIKGGVKEENIVVF
MYDDIAYSEFNPRPGVIINHPQGPNVYDGVPKDYTGDFVTADNLYAVILGDKSKVRGGSGKVINSKAEDRIFIYYSDHGGPGVLGMPNMPYVYAMDFIDVLKKKHAS
RGYQQMVIYVEACESGSVFQGIMPKDIDVYVTTASNAEESSWGTYLVPGVYPASPPEYITCLGDLYSVAWMEDSETHNLKRETLKQQFASVKERTLNNNNYGLGSHV
TEYGDTNITDEKLYLYHGFDPASVNLPPNNGRLESKMEVVNQRDAEILFMWQMYQRLDHQSEKKRDILKKISETVKHRNHLDGSVELIGVLLFGPTRGSSVLQSVRA
SGLPLVDDWECLKSRVRVFETHCGSLTQYGMKHMRAFANICNRGISEDLMEETCMVACGGYDVGLLHPSNKGYSA

>gi:442540375 VPE beta Vitis vinifera
MALHRSVCLLNSALLLLLLLLAEGTGGARALRLSRWDQKIWMPTDKVEAEEDGEERGTKWAVLVAGSYGYGNYRHQADVCHAYQLLKRGGLKDENIVVFMYDDIATH
DFNPRPGVIINHPQGDDVYAGVPKDYTGEDVTAQNLFAVLLGDKSLLKGGSGKVVESKPNDRIFLYYSDHGGQGVLGMPNMPFLYAKDFIDVLKMKHASGSYKEMVL
YVEACESGSIFEGLMPDDLNIYVTTASGPDEESWGTYCPGMEPAPPPEYITCLGDLLSVAWLEDSETHNLKKQTIEDQYQRVKVRTSNHNTYSVGSHVMVYGNESIK
TELLYLYQGFDPATDKLPQNKFDLDIRMDVINQRDADLLFLWQRYKRSKAGSEKKEILKQLTQTMQHRVHLDQSIELIGMLLLGPENGPPLLNAVRPRGLPVVDDWE
CLKSMVVVFETRCGSLTQYGMKHMRAFANICNNGISLTAMEEACRSACSSHTILDQWSPTIRGYSA

>gi:296085092 C13 peptidase Vitis vinifera
MNAYFAVSYSRYQRPLASGSDTNHLRTHKSEPLCMRVTHLFCMSPYTASSLQPSHFKSHALHATPSKHFSLSMALHRSVCLLNSALLLLLLLLAEGTGGARALRLNR
WDQKIWMPTDKVEAEEDGEERGTRWAVLVAGSYGYGNYRHQADVCHAYQLLKRGGLKDENIVVFMYDDIATHDFNPRPGVIINHPQGDDVYAGVPKDYTGEDVTAQN
LFAVLLGDKSLLKGGSGKVVESKPNDRIFLYYSDHGGQGVLGMPNMPFLYAKDFIDVLKMKHASGSYKEMVLYVEACESGSIFEGLMPDDLNIYVTTASGPDEESWG
TYCPGMEPAPPPEYITCLGDLFSVAWLEDSETHNLKKQTIEDQYQRVKVRTSNHNTYSVGSHVMVYGNESIKTELLYLYQGFDPATDKLPQNKFDLDIRMDVINQRD
ADLLFLWQRYKRSKADSEKKEILKQLTQTMQHRVHLDQSIELIGMLLLGPENGPPLLNAVRPRGLPVVDDWECLKSMVVVFETRCGSLTQYGMKHMRAFANICNNGI
SLTAMEEACRSACSSHTILDQWSPTIRGYSA

>gi:413937993 VPE1 Zea mays
MAVRWCLLLLVLVLAAAAACAEKGEWDPVIRMPGEKEPAGSHSHSGEGFDGEVDDAVGTRWAVLVAGSSGYGNYRHQADICHAYQILQKGGIKEENIVVFMYDDIAN
SALNPRQGVIINHPEGEDVYAGVPKDYTGDQVTTKNFYAVLLGNKTAVTGGSRKVINSKADDHIFIYYSDHGGPGVLGMPNLPYLYAGDFMKVLREKHASNSYAKMV
IYIEACESGSIFEGLMPEDLNIYVTTASNAEESSWGTYCPGMEPPPPSEYITCLGDLYSVSWMEDSETNNLKEETIKEQYEVSQHCAQVKKRTSDFNSYGAGSHVME
YGDKTFKGEKLYLYQGFDPANANVTNKLLWSGQEAVVNQRDADILFLWKRYELLHEKSEEKQEVLREITGTVRHRKHLDNSINFIGKLLFGAEKGPSTLEAVRPPGQ
PLVDDWDCLKQMVRIFESHCGSLTQYGMRHMRAFANICNSGTPGASMKRASMGACGGYNSARWSPLAQGYSA

>gi:414586098 VPE beta Zea mays
MMAAAAWLCGLLSLLALAGAASAADGAEGEWEPLIRMPTAKGSDAASAPAAEDDEVGTRWAVLVAGSFGYGNYRHQADVCHAYQILQKGGVKKENIVVFMYDDIAHN
ILNPRPGVIINHPKGANVYDGVPKDYTGDQVTTENFFAVLLGNRSATTGGSKKVIDSKPNDHIFIYYSDHGGPGVLGMPNLPYLYAGDFIKVLKKKHASNSYSKMVI
YVEACESGSIFEGLMPEDLNIYVTTASNPVENSWGTYCPGMEPSPPPEYITCLGDLYSVSWMEDSETHNLKKETIKDQYEVVKTRTSNSNKYKEGSHVMEYGDKTFK
DEKLSFYQGFDPANANIANMLLWPGPKGAVNQRDADLLFMWKRYEQLNGGTEEKLRALIEIKETVQHRKHLDSSIDFVGRLVFGFEKGPSMLEAVRTSGQPLVDDWD
CLKRMVRIFESQCGSLTQYGMKYMRAFANICNSGISEMKMRESSISACSSYNSARWSPMARGHSA

>gi:226493414 C13 peptidase Zea mays
MPTAKGSDAASAPAAEDDEVGTRWAVLVAGSFGYGNYRHQADVCHAYQILQKGGVKKENIVVFMYDDIAHNILNPRPGVIINHPKGANVYDGVPKDYTGDQVTTENF
FAVLLGNRSATTGGSKKVIDSKPNDHIFIYYSDHGGPGVLGMPNLPYLYAGDFIKVLKKKHASNSYSKMVIYVEACESGSIFEGLMPEDLNIYVTTASNPVENSWGT
YCPGMEPSPPPEYITCLGDLYSVSWMEDSETHNLKKETIKDQYEVVKTRTSNSNKYKEGSHVMEYGDKTFKDEKLSFYQGFDPANANIANMLLWPGPKGAVNQRDAD
LLFMWKRYEQLNGGTEEKLRALIEIKETVQHRKHLDSSIDFVGRLVFGFEKGPSMLEAVRTSGQPLVDDWDCLKRMVRIFESQCGSLTQYGMKYMRAFANICNSGIS
EMKMRESSISACSSYNSARWSPMARGHSA
