# Supplementary material for: The Amborella vacuolar processing enzyme family
Source: Front Plant Sci. 2015 Aug 21;6:618. doi: 10.3389/fpls.2015.00618 (PMC4544213; doi:10.3389/fpls.2015.00618)
Supplement: Supplementary file 4 [file Image3.PDF]

Supplemental Figure S3: A phylogeny of an ML Phylogeny of plant VPE (Vacuolar Processing Enzymes) proteins, rooted between seed and non-seed plant VPEs, showing *Amborella* proteins in basal positions of two well-supported clades of angiosperm α/γ/δ-VPEs and β-VPEs, respectively. Figures at nodes give percentage bootstrap support values based on 500 replicate trees.

The figure shows a detailed tree. For a simplified tree, see Figure 2. The amino acid sequences used to construct the phylogenetic tree are listed in Supplemental Figure S3. For the clarity of presentation, a simplified nomenclature has been used consisting of a three-letter code for the species followed by the accession number of the VPE protein (e.g., Ptr-gi224141591 for a *Populus trichocarpa* VPE, accession number gi224141591).

Accession numbers and species-of-origin are shown in Supplemental Figure S2.

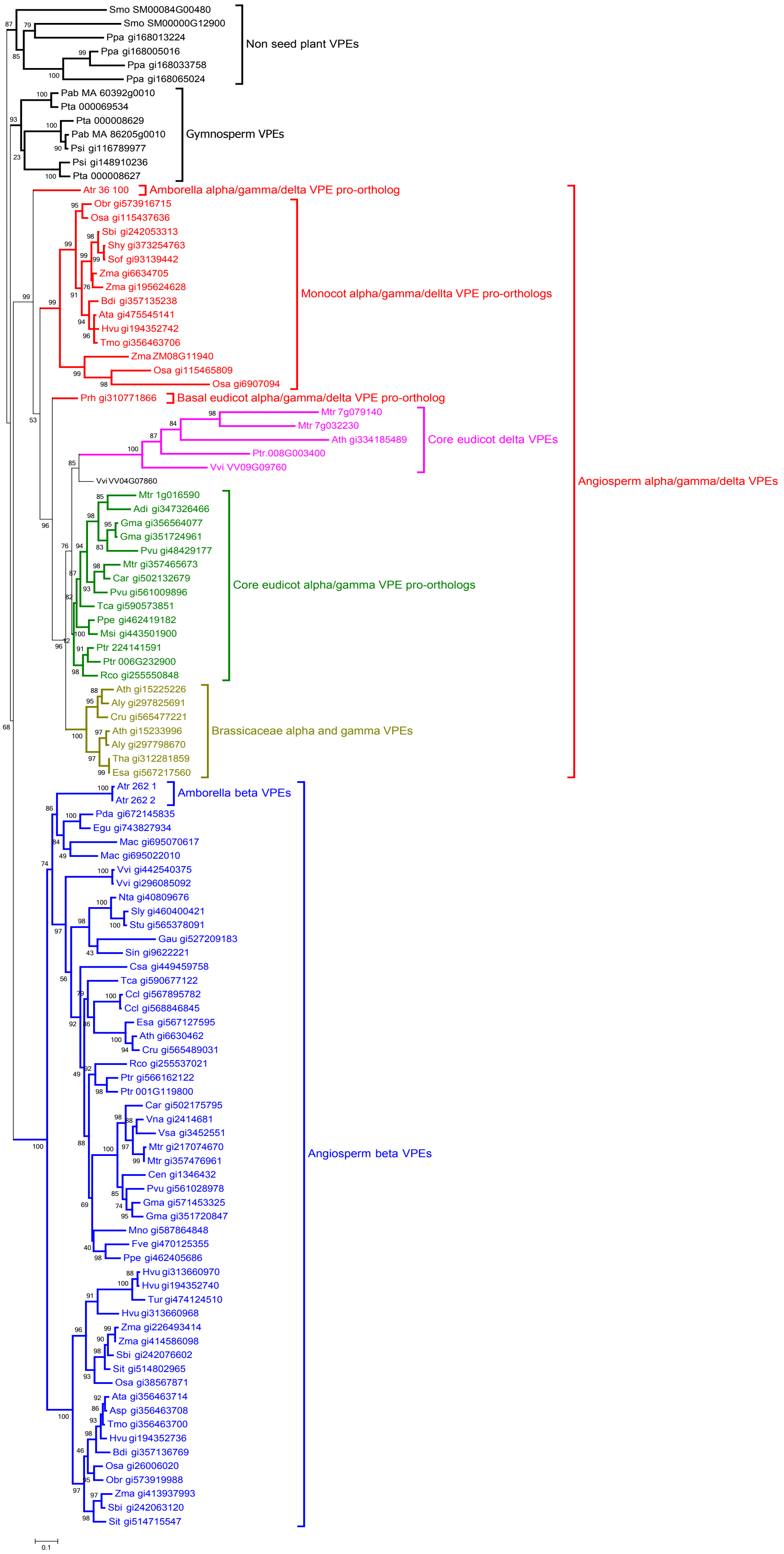
Smo SM00084G00480
Smo SM00000G12900
Ppa gi168013224
Ppa gi168005016
Ppa gi168033758
Ppa gi168065024
Non seed plant VPEs
Pab MA 60392g0010
Pta 000069534
Pta 000008629
Pab MA 86205g0010
Psi gi116789977
Psi gi148910236
Pta 000008627
Gymnosperm VPEs
Atr 36 100
Amborella alpha/gamma/delta VPE pro-ortholog
Obr gi573916715
Osa gi115437636
Sbi gi242053313
Shy gi373254763
Sof gi93139442
Zma gi6634705
Zma gi195624628
Bdi gi357135238
Ata gi475545141
Hvu gi194352742
Tmo gi356463706
Zma ZM08G11940
Osa gi115465809
Osa gi6907094
Monocot alpha/gamma/dellta VPE pro-orthologs
Prh gi310771866
Basal eudicot alpha/gamma/delta VPE pro-ortholog
Mtr 7g079140
Mtr 7g032230
Ath gi334185489
Ptr.008G003400
Vvi VV09G09760
Core eudicot delta VPEs
Vvi VV04G07860
Mtr 1g016590
Adi gi347326466
Gma gi356564077
Gma gi351724961
Pvu gi48429177
Mtr gi357465673
Car gi502132679
Pvu gi561009896
Tca gi590573851
Ppe gi462419182
Msi gi443501900
Ptr 224141591
Ptr 006G232900
Rco gi255550848
Core eudicot alpha/gamma VPE pro-orthologs
Ath gi15225226
Aly gi297825691
Cru gi565477221
Ath gi15233996
Aly gi297798670
Tha gi312281859
Esa gi567217560
Brassicaceae alpha and gamma VPEs
Angiosperm alpha/gamma/delta VPEs
Atr 262 1
Atr 262 2
Amborella beta VPEs
Pda gi672145835
Egu gi743827934
Mac gi695070617
Mac gi695022010
Vvi gi442540375
Vvi gi296085092
Nta gi40809676
Sly gi460400421
Stu gi565378091
Gau gi527209183
Sin gi9622221
Csa gi449459758
Tca gi590677122
Ccl gi567895782
Ccl gi568846845
Esa gi567127595
Ath gi6630462
Cru gi565489031
Rco gi255537021
Ptr gi566162122
Ptr 001G119800
Car gi502175795
Vna gi2414681
Vsa gi3452551
Mtr gi217074670
Mtr gi357476961
Cen gi1346432
Pvu gi561028978
Gma gi571453325
Gma gi351720847
Mno gi587864848
Fve gi470125355
Ppe gi462405686
Hvu gi313660970
Hvu gi194352740
Tur gi474124510
Hvu gi313660968
Zma gi226493414
Zma gi414586098
Sbi gi242076602
Sit gi514802965
Osa gi38567871
Ata gi356463714
Asp gi356463708
Tmo gi356463700
Hvu gi194352736
Bdi gi357136769
Osa gi26006020
Obr gi573919988
Zma gi413937993
Sbi gi242063120
Sit gi514715547
Angiosperm beta VPEs
0.1

Supplemental Figure S3, following: Amino acid sequence alignments of the plant VPEs are shown.

Alignment: C:\Users\cscutt\Desktop\regionsphy
Seaview [blocks=10 fontsize=10 A4] on Tue May 05 20:03:34 2015

```
                   1
Mtr_1g016590       IRLPSQGTRW AILLAGSNGY WNYRHQADVC HAYQLLRKGG LKEENIIVFM YDDIASNVEN
Mtr_7g032230       VFAEGEGKKW AFLVAGSNGY VNYRHQADIC HAYQILKKGG LKDENIVVFM YDDIAYNPQN
Mtr_7g079140       VVRPMVGKKW ALLVAGSKGY SNYRHQSNIC HAYHILKSGG LQDENIIVFM YDDIAYHNEN
Pda_gi672145835    IRMPAEGTRW AVLVAGSFGY GNYRHQADVC HAYQLLKKGG LKEENIVVMM HDDIANNPLN
Egu_gi743827934    IRMPTEGTRW AVLVAGSFGY GNYRHQADVC HAYQLLKKGG LKEENIVVMM HDDIANNPLN
Mac_gi695022010    ---------- ---------- ---------- ---------- --------MM YDDIANSPLN
Mac_gi695070617    IRLPSSGTKW ALLVAGSSGY GNYRHQADVC HAYQLLRRGG LKEENIVVMM HDDIAHNPLN
Atr_262_1          IRMPSQGTRW AVLVAGSSGF GNYRHQADVC HAYQLLLKGG LKEENIVVFM HDDIAYNEFN
Atr_262_2          IRMPSQGTRW AVLVAGSSGF GNYRHQADVC HAYQLLLKGG LKEENIVVFM HDDIAYNEFN
Atr_36_100         LRMPQTGTRW AVLVAGSSGY GNYRHQADIC HAYQTMIRGG LKEKNIVVFM YDDIAYNEEN
Asp_gi356463708    IRMPGEGTRW AVLVAGSSGY GNYRHQADIC HAYQILRKGG VKEENIVVFM YDDIANNPLN
Ata_gi356463714    IRMPGEGTRW AVLVAGSSGY GNYRHQADIC HAYQILRKGG VKEENIVVFM YDDIANNPLN
Ata_gi475545141    IRLPSQGTRW AVLIAGSNGY YNYRHQADIC HAYQIMKKGG LKDENIIVFM YNDIAHNPEN
Aly_gi297825691    IKLPSQSTRW AVLVAGSSGY WNYRHQADVC HAYQLLKKGG VKEENIVVFM YDDIAKNEEN
Aly_gi297798670    IKLPSQGTRW AVLVAGSSGY WNYRHQADIC HAYQLLRKGG LKEENIVVFM YDDIANNYEN
Ath_gi334185489    TQLLNDGTRW AVLVAGSNEY YNYRHQADIC HAYQILRKGG LKDENIIVFM YDDIAFSSEN
Ath_gi6630462      ILMPTEGTRW AVLVAGSSGY GNYRHQADVC HAYQILRKGG LKEENIVVLM YDDIANHPLN
Ath_gi15233996     IKLPSQGTRW AVLVAGSSGY WNYRHQADIC HAYQLLRKGG LKEENIVVFM YDDIANNYEN
Ath_gi15225226     IKLPSLSTKW AVLVAGSSGY WNYRHQADVC HAYQLLKKGG VKEENIVVFM YDDIAKNEEN
Adi_gi347326466    LRLPSEGTKW AVLLAGSNGY WNYRHQADIC HAYQLLRSGG VKEENIIVFM FDDIAYSEEN
Bdi_gi357136769    IRMPGEGTRW AVLIAGSSGY GNYRHQADIC HAYQVLRKGG LKEENIVVFM YDDIANSALN
Bdi_gi357135238    IRLPSQGTRW AVLIAGSNGY YNYRHQADIC HAYQIMKKGG LKDENIIVFM YDDIAHNPEN
Cen_gi1346432      IQLPTEGTRW AVLVAGSNGY GNYRHQADVC HAYQLLIKGG VKEENIVVFM YDDIAYNAMN
Cru_gi565477221    IRLPSESTRW AVLVAGSSGY WNYRHQADVC HAYQLLKKGG VKEENIVVFM YDDIANNEEN
Cru_gi565489031    ILMPTEGTRW AVLVAGSSGY GNYRHQADVC HAYQILRKGG LKEENIVVMM YDDIANHQLN
Car_gi502175795    IRLPTEGTRW AVLVAGSSGY GNYRHQADVC HAYQLLVKGG VKEENIVVFM YDDIAQNELN
Car_gi502132679    LRLPSEGTRW AILIAGSNGY WNYRHQSDVC HAYQVLRKGG LKEENIVVFM YDDIAFNEEN
Ccl_gi567895782    ILLPSEGTRW AVLVAGSSGY ANYRHQADVC HAYQLLRKGG LKEEHIVVFM YDDIAMHELN
Ccl_gi568846845    ILLPSEGTRW AVLVAGSSGY ANYRHQADVC HAYQLLRKGG LKEEHIVVFM YDDIAMHELN
Csa_gi449459758    IRMPTEGTRW AVLIAGSSGF GNYRHQADIC HAYQLLKKGG LKDENIVVFM YDDIATNVLN
Esa_gi567127595    ILMPTEGTRW AVLVAGSSGY GNYRHQADVC HAYQILRKGG LKEENIVVLM YDDIANHPLN
Esa_gi567217560    IKLPSQGTRW AVLVAGSNGY WNYRHQADIC HAYQLLRKGG VKEDNIVVFM YDDIANNEEN
Fve_gi470125355    IRLPSEGTTW AVLVAGSNGY GNYRHQADVC HAYQILKKGG LKEENIVVFM YDDIAMHEMN
Gau_gi527209183    IRWPFHGTRW AVLVAGSNGF GNYRHQADVC HAYQILKRGG LKEENIVVFM YDDIADSEMN
Gma_gi351724961    LRLPSEGTRW AVLLAGSNGY WNYRHQADVC HAYQILRKGG LKEENIIVFM YDDIAFNGEN
Gma_gi356564077    LRLPSEGTRW AVLLAGSNGY WNYRHQADVC HAYQILRKGG LKEENIIVFM YDDIAFNGEN
Gma_gi571453325    IKLPAEGTRW AVLVAGSNGY GNYRHQADVC HAYQLLIKGG LKEENIVVFM YDDIATDELN
Gma_gi351720847    IKLPTEGTRW AVLVAGSNGY GNYRHQADVC HAYQLLIKGG LKEENIVVFM YDDIATNELN
Hvu_gi194352742    IRLPSQGTRW AVLIAGSNGY YNYRHQADIC HAYQIMKKGG LKDENIIVFM YDDIARNPEN
Hvu_gi194352740    IRLPTEVTKW AVLVAGSSGY ENYRHQADVC HAYQILKKGG LKDENIVVFM YDDIANSPEN
Hvu_gi194352736    IRMPGEGTRW AVLVAGSSGY GNYRHQADIC HAYQILRKGG VKEENIVVFM YDDIAKNALN
Hvu_gi313660970    IRLPTEVTKW AVLVAGSSGY ENYRHQADVC HAYQILKKGG LKDENIVVFM YDDIANSPEN
Hvu_gi313660968    IRLPTDVTKW AVLVAGSSGY GNYRHQADVC HAYQILRQGG LKEENIVVFM FDDIAKNHLN
Msi_gi443501900    LRLPSEGTRW AVLIAGSNGY WNYRHQADIC HAYQLLKKGG LKDENIVVFM YDDIAYNEEN
Mtr_gi357476961    IRLPGEGTRW AVLVAGSSGY GNYRHQADVC HAYQLLIKGG VKEENIVVFM YDDIANNELN
Mtr_gi217074670    IRLPGEGTRW AVLVAGSSGY GNYRHQADVC HAYQLLIKGG VKEENIVVFM YDDIANNELN
Mtr_gi357465673    LRLPSQGTKW AILIAGSNGY WNYRHQSDVC HAYQVLRKGG LKEENIIVFM YDDIADNQEN
Mno_gi587864848    IRMPTDSTRW AVLVAGSSGY GNYRHQADVC HAYQLLRKGG VKEENIVVFM YDDIAMHGLN
Nta_gi40809676     IRSPVDGVRW AVLVAGSNGY GNYRHQADVC HAYQILKRGG LKDENIVVFM YDDIAKSELN
Obr_gi573916715    LLLPSEGTRW AVLIAGSNGY YNYRHQADVC HAYQIMKKGG LKDENIIVFM YDDIAHNSEN
Obr_gi573919988    IRMPGEGTRW AVLVAGSSGY GNYRHQADVC HAYQILRKGG LKEENIVVFM YDDIANNALN
Osa_gi26006020     IRMPGEGTRW AVLVAGSSGY GNYRHQADVC HAYQILRKGG LKEENIVVFM YDDIANNILN
Osa_gi115437636    LRLPSEGTRW AVLIAGSNGY YNYRHQADVC HAYQIMKRGG LKDENIIVFM YDDIAHNPEN
Osa_gi115465809    LRLPSSTTRW AVLIAGSNGF YNYRHQADVC HAYQIMRKGG VEEQNIVVMM YDDIAHNPDN
Osa_gi6907094      LRLPTEGTKW ALLIAGSKGY ENYRHQADVC HAYQIMKKGG LKDQNIVVMM YDDIAYNPEN
Osa_gi38567871     IRMPTEGTRW AVLVAGSSGY GNYRHQADVC HAYQILQKGG VKEENIVVFM YDDIAHNILN
Prh_gi310771866    IRLPSEGTRW AVLIAGSSGY WNYRHQADVC HAYQVLKRGG VKDENIVVFM YDDIALNEEN
Pvu_gi48429177     LRLPSDGTRW AILFAGSSGY WNYRHQADIC HAYQLLRKGG LKDENIIVFM YDDIAFNSEN
Pvu_gi561009896    LRLPSEGTRW ALLIAGSNGY WNYRHQSDVC HAYQLLRKGG LKEENIVVFM YDDIAFNEEN
Pvu_gi561028978    IKLPTQGTRW AVLVAGSNGY GNYRHQADVC HAYQLLIKGG VKEENIVVFM YDDIATHELN
Ppa_gi168013224    IVMPTEGQRW AVLVAGSSGY GNYRHQADVC HAYQILKKGG MKDENIVVFM FDDIAHNRHN
Ppa_gi168033758    --MPTGGTRW AILIAGSAGY WNYRHQADVC HAYQILKRGG LKDENIIVFM HDDIAYHPEN
Ppa_gi168005016    --MPTEGTRW AILIAGSSGY WNYRHQADVC HAYQILKRGG LKEENIVVFM YDDIAYSTEN
Ppa_gi168065024    --MPIDGVRW AILIAGSSGY GNYRHQADIC HAYQILKRGG LKEENIVVFM YDDIANNEEN
Pab_MA_60392g0010  IRMPIYSTRW AVLLAGSSGY GNYRHQADVC HAYQILRKGG LKEENIIVFM YDDIANNSDN
Pab_MA_86205g0010  IRLSTAGTQW AVLLAGSAGY SNYRHQADVC HAYQILKRGG LKDENIVVFM YDDIANNPVN
Psi_gi148910236    LKLPTNGTEW AVLLAGSSGY WNYRHQADVC HAYQILRRGG LKEENIVVFM YDDIAYDEEN
Psi_gi116789977    IRLSTEGTQW AVLLAGSAGY SNYRHQADVC HAYQILKRGG LKDENIVVFM YDDIANNPVN
Pta_000008627      LKMPTDGTQW AILLAGSSGY WNYRHQADIC HAYQILRKGG LKEENIVVFM YDDIAQDEEN
Pta_000069534      IRMPVDGTRW AVLLAGSSGY GNYRHQADVC HAYQILRKGG LKEENIIVFM YDDIANNSDN
Pta_000008629      IRLSTDGTQW AVLLAGSAGY SNYRHQADVC HAYQILKRGG LKDENIIVFM YDDIANNPAN
Ptr_006G232900     LRLPSEGTRW AILLAGSNGY WNYRHQADVC HAYQLLRQGG LKEENIIVFM YDDIADNPEN
Ptr_gi566162122    ILMPTEGTRW AVLVAGSNGY GNYRHQADVC HAYQLLRKGG IKEENIVVFM YDDIAKHEFN
Ptr_001G119800     ILMPTGGSRW AVLVAGSSGY GNYRHQADVC HAYQLLRKGG IKEENMVVFM YDDIAMHHLN
Ptr_224141591      LRLPSEGTRW AILLAGSNGY WNYRHQADVC HAYQLLRKGG LKEENIIVFM YDDIAYNSEN
```

Ptr.008G003400	SSLPSSGKQW	AVLVAGSAGY	ENYRHQADVC	HAYQILKKGG	LKDENIIVFM	YDDIAFHVDN
Ppe_gi462419182	LRLPSEGTRW	AVLIAGSNGY	WNYRHQADIC	HAYQLLKKGG	LKDENIVVFM	YDDIAYNEEN
Ppe_gi462405686	IRLPSDGTRW	AVLVAGSSGY	GNYRHQADVC	HAYQLLKKGG	LKEENIVVFM	YDDIANHEIN
Rco_gi255537021	ILMPTEGTRW	AVLVAGSMGF	GNYRHQADVC	HAYQLLRKGG	LKEENIIVFM	YDDIAKNELN
Rco_gi255550848	IRLPSEGTRW	AILIAGSNGY	WNYRHQADVC	HAYQLLRKGG	LKEENIIVFM	YDDIAYNEEN
Shy_gi373254763	IRLPSEGTRW	AVLVAGSSGY	YNYRHQADIC	HAYQIMKKGG	LKDENIIVFM	YDDIAHSAEN
Sof_gi93139442	IRLPSEGTRW	AVLVAGSSGY	YNYRHQADIC	HAYQIMKKGG	LKDENIIVFM	YDDIAHSAEN
Smo_SM00084G00480	--MPSSGTRW	ALLVAGSSGF	GNYRHQADVC	HAYQLLRSNG	LKEENIVVMM	FDDIANNTDN
Smo_SM00000G12900	SSASDEGTRW	AVLLAGSAGY	WNYRHQADVC	HAYQLLRRGG	MREENIVVFM	YDDIANNFAN
Sin_gi9622221	IRWPLDATRW	AVLVAGSNGF	GNYRHQADVC	HAYQILKKGG	LRDENIIVFM	YDDIAMNELN
Sit_gi514715547	IRMPGEGTRW	AVLVAGSSGY	GNYRHQADIC	HAYQILLKGG	LKEENIVVFM	YDDVANSVLN
Sit_gi514802965	IRMPTEGTRW	AVLVAGSSGY	GNYRHQADVC	HAYQILLKGG	VKEENIVVFM	YDDIAHNILN
Sly_gi460400421	IRSPVDGVRW	AVLVAGSNGY	GNYRHQADVC	HAYQILKRGG	LNDENIVVFM	YDDIAKSELN
Stu_gi565378091	IRSPVDGVRW	AVLVAGSNGY	GNYRHQADVC	HAYQILKRGG	LKDENIVVFM	YDDIAKSELN
Sbi_gi242063120	IRMPGEGTRW	AVLVAGSSGY	GNYRHQADIC	HAYQILRKGG	IKEENIVVFM	YDDVATSALN
Sbi_gi242076602	IRMPTGGTRW	AVLVAGSSGY	GNYRHQADVC	HAYQILRKGG	VKEENIVVFM	YDDIAHNILN
Sbi_gi242053313	IRLPSDGTRW	AVLVAGSNGY	YNYRHQADIC	HAYQIMKKGG	LKDENIIVFM	YDDIAHSPEN
Tha_gi312281859	IKLPSQGTRW	AVLVAGSNGY	WNYRHQADIC	HAYQLLRKGG	VKEDNIVVFM	YDDIANNEEN
Tca_gi590677122	IRLSTDGTRW	AVLVAGSSGY	ANYRHQADVC	HAYQLLRKGG	LKEENIVVFM	YDDIAMHKLN
Tca_gi590573851	LRLPSEGTRW	AVLIAGSNGY	WNYRHQADVC	HAYQLLKKGG	LKDENIIVFM	YDDIAFNEEN
Tmo_gi356463700	IRMPGEGTRW	AVLVAGSSGY	GNYRHQADIC	HAYQILRKGG	VKEENIVVFM	YDDIANNPLN
Tmo_gi356463706	IRLPSQGTRW	AVLIAGSNGY	YNYRHQADIC	HAYQIMKKGG	LKDENIIVFM	YDDIAHNLEN
Tur_gi474124510	ASAPEEVTKW	AVLVAGSSGY	ENYRHQADVC	HAYQILKKGG	LKDENIVVFM	YDDIANSPEN
Vna_gi2414681	IRLPGEGTRW	AVLVAGSNGY	GNYRHQADVC	HAYQLLIKGG	VKEENIVVFM	YDDIAYNEMN
Vsa_gi3452551	IRLPGEGTRW	AVLVAGSNGY	GNYRHQADVC	HAYQLLIKGG	VKEENIVVFM	YDDIAYSEFN
Vvi_gi442540375	IWMPTDGTKW	AVLVAGSYGY	GNYRHQADVC	HAYQLLKRGG	LKDENIVVFM	YDDIATHDFN
Vvi_gi296085092	IWMPTDGTRW	AVLVAGSYGY	GNYRHQADVC	HAYQLLKRGG	LKDENIVVFM	YDDIATHDFN
Vvi_VV04G07860	LLLPSEGTRW	AVLIAGSNGY	WNYRHQADIC	HAYQLLKKGG	LKDENIIVFM	YDDISFNEEN
Vvi_VV09G09760	LDRPSNGKQW	AVLIAGSTDY	ENYRHQADIC	HAYQILKKGG	LKDENIVVFM	YDDIAFNVEN
Zma_gi413937993	IRMPGEGTRW	AVLVAGSSGY	GNYRHQADIC	HAYQILQKGG	IKEENIVVFM	YDDIANSALN
Zma_gi414586098	IRMPTAGTRW	AVLVAGSFGY	GNYRHQADVC	HAYQILQKGG	VKKENIVVFM	YDDIAHNILN
Zma_gi226493414	ASAPAAGTRW	AVLVAGSFGY	GNYRHQADVC	HAYQILQKGG	VKKENIVVFM	YDDIAHNILN
Zma_gi195624628	IRLPSQGTRW	AVLIAGSSGY	YNYRHQADIC	HAYQIMKKGG	LKDENIIVFM	YDDIAHSPEN
Zma_ZM08G11940	LRLPSEGTRW	AVLIAGSNGY	YNYRHQADVC	HAYQVLKKGG	LKDENIVVFM	YDDIADSPDN
Zma_gi6634705	IRLPSDGTRW	AVLIAGSNGY	YNYRHQADIC	HAYQIMKKGG	LKDENIVVFM	YDDIAHSPEN

```
                      61
Mtr_1g016590          PRPGVIINKP DGGDVYEGVP KGAEVHADNF YAALLGNKSA LGGSGKVVDS GPNDHIFVYY
Mtr_7g032230          PRRGVLINHP NGSDVYNGVP KGDYGNLENF LAVLSGNKSA TGGSGKVLDT GPDDTIFIFY
Mtr_7g079140          PRPGVIINRP DGPNVYPGVP KGNNTNAENF FAVLNGNLSG IGGSGKVLNS DPNDTIFIYY
Pda_gi672145835       PRPGVIINHP DGEDVYAGVP KGEQVTTKNL YAVILGNKSA VGGSGKVVDS KPVDRIFIYY
Egu_gi743827934       PRPGVIINHP QGEDVYAGVP KGEHVTTTNL YAVILGNKSA VGGSGKVVDS KPNDRIFIYY
Mac_gi695022010       PRPGVIINHP QGHDVYAGVP KGKQVTSKNL YAVLLGIKSA VGGSGKVIDS KPNDRIFIYY
Mac_gi695070617       PRQGVIINHP QGQDVYAGVP KKEQVTAKNL YAVLLGDRSA IGGSGKVIDS KPDDRIFIYY
Atr_262_1             PKKGIIINHP QGEDVYAGVP KGKQVHTKNL YAVLLGNNSA VGGSGKVINS KAEDRIFIYY
Atr_262_2             PKKGIIINHP QGEDVYAGVP KGKQVHTKNL YAVLLGNNSA VGGSGKVINS KAEDRIFIYY
Atr_36_100            PRPGVIINRP HGEDVYAGVP KGDDVNVDNL FAVILGNKSA LGGSGKVVDS GPDDHIFIFY
Asp_gi356463708       PRPGVIINHP EGEDVYAGVP KGEAVTAKNF YAVLLGNNTA VGGSKKVIDS KPNDHIFIYY
Ata_gi356463714       PRPGVIINHP EGEDVYAGVP KGEAVTAKNF YAVLLGNKTA VGGSKKVIDS KPNDHIFIYY
Ata_gi475545141       PRPGVIINHP QGGDVYAGVP KGKEVNVKNF FAVLLGNKTA VGGSGKVVDS GPNDHIFVFY
Aly_gi297825691       PRPGVIINSP NGEDVYNGVP KGDDVNVDNL LAVILGNKTA VGGSGKVVDS GPNDHIFIYY
Aly_gi297798670       PRPGTLINSP HGKDVYQGVP KGDDVNVDNL FAVILGDKTA VGGSGKVVDS GPNDHIFIFY
Ath_gi334185489       PRPGVIINKP DGEDVYKGVP KKEAVNVQNF YNVLLGNESG VGGNGKVVKS GPNDNIFIYY
Ath_gi6630462         PRPGTLINHP DGDDVYAGVP KGSSVTAANF YAVLLGDQKA VGGSGKVIAS KPNDHIFVYY
Ath_gi15233996        PRPGTIINSP HGKDVYQGVP KGDDVNVDNL FAVILGDKTA VGGSGKVVDS GPNDHIFIFY
Ath_gi15225226        PRPGVIINSP NGEDVYNGVP KGDEVNVDNL LAVILGNKTA LGGSGKVVDS GPNDHIFIYY
Adi_gi347326466       PRPGVIINKP DGGDVYKGVP KGKDVNVNNF FAALLGNKSA LGGSGKVVDS GPNDHIFVFY
Bdi_gi357136769       PRPGVIINHP QGEDVYAGVP KGEQVTAKNL YAVLLGNKTA VGGSKKVIDS QPKDHIFIYY
Bdi_gi357135238       PRPGVIINHP QGGDVYAGVP KGKEVNVKNF FAVLLGNKAA VGGSGKVVDS GPNDHIFVFY
Cen_gi1346432         PRPGVIINHP QGPDVYAGVP KGEDVTPENL YAVILGDKSK VGGSGKVINS NPEDRIFIFY
Cru_gi565477221       PRPGVIINSP NGEDVYNGVP KGDEVNVNNF FAVLLGNKTA LGGSGKVVNS GPNDHIFIYY
Cru_gi565489031       PRPGTLINHP NGEDVYAGVP KGSNVTAANF YAVLLGDQKA VGGSGKVIAS KPNDHIFVYY
Car_gi502175795       PRPGVIINHP QGPDVYAGVP KGDSVTAENL YAVILGDKSK VGGSGKVINS KAEDRIFIYY
Car_gi502132679       PRPGVIINSP HGDDVYKGVP KGEDVNVDNF FAALLGNKSA LGGSGKVVDS GPNDHIFIYY
Ccl_gi567895782       PRPGVIINHP QGENLYDGVP KGEHVTAQNL YAVLLGDRKA VGGSGKVVNS KANDRIFIFY
Ccl_gi568846845       PRPGVIINHP QGENLYDGVP KGEHVTAQNL YAVLLGDRKA VGGSGKVVNS KANERIFIFY
Csa_gi449459758       PRPGIIINHP QGEDVYAGVP KGEHVTAQNL YAVLLGNRTA VGGSGKVVDS KPNDRIFVYY
Esa_gi567127595       PRPGTLINHP DGEDVYAGVP KGHDVTAANF YAVLLGDKKA VGGSGKVIAS KPNDHIFVYY
Esa_gi567217560       PRRGIIINSP HGKDVYQGVP KGDDVTVDNL FAVILGNKTA TGGSGKVVDS GPNDHIFIFY
Fve_gi470125355       PRKGIIINHP QGQDVYAGVP KGEHVNAANL YAVLLGDKKA VGGTGKVVAS KPNDRIFLFY
Gau_gi527209183       PRKGTIINHP NGRDVYEGVP KGENVTAANF YAVIQGNKTG VGGSGKVVDS KPNDHVFIYY
Gma_gi351724961       PRPGVIINKP DGGDVYKGVP KGEDVTVDNF FAALLGNKSA LGGSGKVVDS GPDDHIFVYY
Gma_gi356564077       PRPGVIINKP DGGDVYEGVP KGEDVTVGNF FAALLGNKSA LGGSGKVVDS GPDDHIFVYY
Gma_gi571453325       PRPGVIINHP EGQDVYAGVP KGENVTAQNL FAVILGDKNK VGGSGKVINS KPEDRIFIYY
Gma_gi351720847       PRHGVIINHP EGEDLYAGVP KGDNVTTENL FAVILGDKSK LGGSGKVINS KPEDRIFIYY
Hvu_gi194352742       PRPGVIINHP QGGDVYAGVP KGKEVNVKNF FAVLLGNKTA VGGSGKVVDS GPNDHIFVFY
Hvu_gi194352740       PRRGVVINHP KGKDVYHGVP KGDQVTAKNF YAVLLGNKTA VGGSRKVINS KPEDHIFIYY
Hvu_gi194352736       PRPGVIINHP EGEDVYAGVP KGEAVTAKNF YAVLLGNKTA VGGSKKVIDS KSNDHIFIYY
Hvu_gi313660970       PRRGVVINHP KGKDVYHGVP KGDQVTAKNF YAVLLGNKTA VGGSRKVINS KPEDHIFIYY
Hvu_gi313660968       PRPGVIINHP KGEDVYAGVP KGGQVTAKNF FAVLLGNKTA VGGSGKVINS KPKDHIFIYY
Msi_gi443501900       PRQGVIINSP HGSDVYEGVP KGEDVTVNNF FAAILGNKTA LGGSGKVVDS GPNDHIFIYY
Mtr_gi357476961       PRPGVIINHP QGPNVYVGVP KGDNVTAENL YAVILGDKSK VGGSGKVINS KSEDRIFIYY
Mtr_gi217074670       PRPGVIINHP QGPNVYVGVP KGDNVTAENL YAVILGDKSK VGGSGKVINS KSEDRIFIYY
Mtr_gi357465673       PRPGVIINSP HGDDVYKGVP KGDDVNVNNF FAALLGNKSA LGGSGKVVDS GPNDHIFIYY
Mno_gi587864848       PRPGIIINHP QGDDVYAGVP KGQYVTTENL YAVLLGDKKA VGGSGKVVDS KPNDRIFLYY
Nta_gi40809676        PRPGVIINHP NGSDVYAGVP KGEHVTAANL YAVLLGDKSA VGGSGKIVDS KPNDRIFLYY
Obr_gi573916715       PRPGVIINHP QGGDVYAGVP KGKDVNVKNL FAVLLGDKTA VGGSGKVLDS GPNDHIFIFY
Obr_gi573919988       PRPGVIVNHP QGEDVYAGVP KGDEVNTKNF YAVLLGNKTA VGGSRKVIDS KPNDHIFIFY
Osa_gi26006020        PRPGVIVNHP QGEDVYAGVP KGDEVTAKNF YAVLLGNKTA VGGSRKVIDS KPNDHIFIFY
Osa_gi115437636       PRPGVIINHP QGGDVYAGVP KGKEVNVKNL FAVLLGNKTA VGGSGKVLDS GPNDHIFIFY
Osa_gi115465809       PRPGLIFNHP SGPDVYAGVP KGDDVNVNNF LAVLLGNRSA LSGSGKVVAS GPNDHVFVYY
Osa_gi6907094         PHKGVIINKP NGPNVYAGVP KGNDVNKNNF LAVLLGKKSA LAGSGKVISS GPNDHIFVYY
Osa_gi38567871        PRPGTIINHP KGGDVYAGVP KGHQVTTENF FAVLLGNKTA VGGSGKVIDS KPEDHIFIYY
Prh_gi310771866       PRPGVIINHP KGEDVYAGVP KGRDVTAHNF YSVLLGNKTA VGGSGKVIDS GPNDHIFIYY
Pvu_gi48429177        PRRGVIINSP NGDEVYKGVP KGEDVTAHNF YAALLGDKSK LGGSGKVVNS GPNDHIFIFY
Pvu_gi561009896       PRPGVIINSP HGNDVYKGVP KGEDVTVNNF FAAILGNKSA LGGSGKVIDS GPNDHIFIYY
Pvu_gi561028978       PRPGVIINNP QGPDVYAGVP KGESVTSRNF FAVLLGDKSK VGGSGKVINS KPEDRIFVYY
Ppa_gi168013224       PRPGVILNHP NGEDVYHGVP KGKNVTVNNL LAVLLGDKKT LGGSGKVVNS GPNDHIFIYY
Ppa_gi168033758       PYPGTIINKP DGPDVYQGVP KGSDVTVSNL YAAILGDKSA IGGTGKVVDS GPNDHIFIYY
Ppa_gi168005016       PHPGKIINKP DGPDVYQGVP KGADVTVSNF YAALLGDKDA IGGSGKVVNS GPNDHIFIYY
Ppa_gi168065024       PHRGKVFNKP YGPDVYPGVP KGENITVSNF YAAILGDADA TGGSGKVVAS GPNDHVFIYY
Pab_MA_60392g0010     PRPGTIINHP NGKDVYAGVP KGDNVNVNNF LAVLLGDKSS VGGSGKVVDS GPNDHIFIYY
Pab_MA_86205g0010     PRPGIIINHP EGSDVYAGVP KGKEVTVDNF FAVILGDKDS VGGSGKVVDS GPNDHIFIYY
Psi_gi148910236       PHPGTIINHP QGSDVYAGVP KGEDVTVNNF FAAILGNKSL VGGSGKVVES GPNDRIFIYY
Psi_gi116789977       PRPGIIINHP EGSDVYAGVP KGKEVTVDNF FAVILGDKDS VGGSGKVVDS GPNDHIFIYY
Pta_000008627         PNPGTIINHP QGSDVYAGVP KGENVTVNNF FAVLLGNKSL VGGSGKVVES DPNDRIFIYY
Pta_000069534         PRPGTIINHP NGKDVYAGVP KGDNVNVNNF LAVLLGDKSS VGGSGKVVDS GPNDHIFIYY
Pta_000008629         PRPGIIINHP EGSDVYAGVP KGKEVTVDNF FAVILGDKDS VGGSGKVVDS GPNDHIFIYY
Ptr_006G232900        PRPGVIINNP QGEDVYKGVP KGPDVTVGNF FAAILGNKTA LGGSGKVIDS GPNDHIFIYY
Ptr_gi566162122       PRPGVIINHP QGDDVYAGVP KGVQVTTENL YAVLLGNKSA VGGSGKVVDS MPNDRIFLYY
Ptr_001G119800        PRPGVIINHP QGDDVYAGVP KGEQVNTENL YAVLLGNKSA VGGSGKVVDS KPNDRIFLYY
Ptr_224141591         PRRGVIINSP QGEDVYKGVP KGEDVTVGNF FAAILGNKTA LGGSGKVVDS GPNDHIFIYY
Ptr.008G003400        PRPGIIINKP FGHDVYAGVP KGDNCTVDNL FAVLLGNKSA LGGSGKVVDS GPNDNIFIYY
Ppe_gi462419182       PRPGVIINSP HGDDVYKGVP KGDDVTVNNF FAAILGNKTA LGGTGKVVDS GPNDHIFIYY
Ppe_gi462405686       PRPGVIINHP QGQDVYAGVP KGKQVTAANL YAVLLGDKKA VGGSGKVVDS KPNDRIFLYY
```

```
Rco_gi255537021     PRPGVIINHP QGEDVYAGVP KGEHVTAKNL YAVLLGDKSA VGGSGKVVDS KPNDRIFLYY
Rco_gi255550848     PRQGIIINNP HGEDVYKGVP KGENVTVGNF FAAILGNRTA LGGRGKVVDS GPNDHIFVYY
Shy_gi373254763     PRPGVVINHP QGGDVYAGVP KGRQVSVNNF FAVLLGNKTA LGGSGKVVDS GPNDHIFVFY
Sof_gi93139442      PRPGVVINHP QGGDVYAGVP KGRQVSVNNF FAVLLGNKTA LGGSGKVVDS GPNDHIFVFY
Smo_SM00084G00480   PRPGTIINHP QGSDVYAGVP KGAAVTAENF LAVLLGDKNS TGGTGKVVSS GPEDHVFLFY
Smo_SM00000G12900   PRPGVMINHP NGDNVYAGVP KGDQVTVNNF LAVLRGDKEA LGGSGKVVES GPNDHIFVFY
Sin_gi9622221       PRKGVIINHP TGGDVYAGVP KGEQVTAENL YAVILGDKSA IGGSGKVVDS KPNDRIFIYY
Sit_gi514715547     PRQGVIINHP EGEDVYAGVP KGEQVTAKNF YAVLLGNKDA VGGSRKVINS KPNDHIFIYY
Sit_gi514802965     PRPGVIINHP KGENVYPGVP KGDQVTTENF FAVLLGNRSA IGGSKKVIDS KPNDHIFIYY
Sly_gi460400421     PRPGVIINHP NGSDVYAGVP KGEHVTAANL YAVLLGDKSA VGGSGKVVNS GPNDRIFLYY
Stu_gi565378091     PRPGVIINHP NGSDVYAGVP KGEHVTAANL YAVLLGDKSA VGGSGKVVNS GPNDRIFLYY
Sbi_gi242063120     PRQGVIINHP QGEDVYAGVP KGDQVTAKNF FAVLLGNKTA VGGSRKVINS KPDDHIFICY
Sbi_gi242076602     PRPGVIINHP KGENVYNGVP KGDQVTTENF FAVLLGNKSA IGGSKKVIDS KPNDHIFIYY
Sbi_gi242053313     PRPGVLINHP QGGDVYAGVP KGREVSVNNF FAVLLGNKTA LGGSGKVVDS GPNDHIFVFY
Tha_gi312281859     PRRGIIINSP HGKDVYQGVP KGDDVTVDNL FAVILGNKTA TGGSGKVVDS GPNDHIFIFY
Tca_gi590677122     PRPEVIINHP KGDDVYAGVP KGVHVTAANL YAVLLGNNSA LGGSGKVVDS KPNDRIFLFY
Tca_gi590573851     PRPGIIINSP HGDDVYEGVP KGEDVTVNNL LAAILGNKTA LGGSGKVVDS GPNDHIFIYY
Tmo_gi356463700     PRPGVIINHP EGEDVYAGVP KGEEVTAKNF YAVLLGNKTA VGGSKKVIDS KPNDHIFIYY
Tmo_gi356463706     PGPGVIINHP QGGDVYAGVP KGKEVNVKNL FAVLLGNKTA VGGSGKVVDS GPNDHIFVFY
Tur_gi474124510     PRRGVVINHP KGKDVYHGVP KGEHVTAKNL YAVLLGNKTA VGGSRKVINS KPNDHIFIYY
Vna_gi2414681       PRPGVIINHP QGPNVYDGVP KGDFVTAENF YAVILGDKSK VGGSGKVINS KAEDRIFIYC
Vsa_gi3452551       PRPGVIINHP QGPNVYDGVP KGDFVTADNL YAVILGDKSK VGGSGKVINS KAEDRIFIYY
Vvi_gi442540375     PRPGVIINHP QGDDVYAGVP KGEDVTAQNL FAVLLGDKSL LGGSGKVVES KPNDRIFLYY
Vvi_gi296085092     PRPGVIINHP QGDDVYAGVP KGEDVTAQNL FAVLLGDKSL LGGSGKVVES KPNDRIFLYY
Vvi_VV04G07860      PRPGIIINSP HGEDVYEGVP KGEDVTVDNF FAVILGNKTA LGGSGKVLDS GPNDHIFIYY
Vvi_VV09G09760      PRPGVIINQP GGDDVYEGVP KQSAATVANV FAVLLGNKTA VGGSGKVLDS GPDDHVFIYY
Zma_gi413937993     PRQGVIINHP EGEDVYAGVP KGDQVTTKNF YAVLLGNKTA VGGSRKVINS KADDHIFIYY
Zma_gi414586098     PRPGVIINHP KGANVYDGVP KGDQVTTENF FAVLLGNRSA TGGSKKVIDS KPNDHIFIYY
Zma_gi226493414     PRPGVIINHP KGANVYDGVP KGDQVTTENF FAVLLGNRSA TGGSKKVIDS KPNDHIFIYY
Zma_gi195624628     PRPGVIINHP QGGDVYAGVP KGRDVNVDNF FAVLLGNKTA LGGSGKVVDS GPDDHIFVFY
Zma_ZM08G11940      PRPGVIINHP SGGDVYAGVP KGKDVNANNF LAALLGNRSA VGGSGKVVAS GPADHVFVYY
Zma_gi6634705       PRPGVIINHP QGG----GVP KGRDVNVDNF FAVLLGNKTA LGGSGKVVDS GPNDHISVFY
```

121

Mtr_1g016590	TDHGGPGVLG	MPPYLYASDL	NEVLKKKHAS	GSYKSLVFYL	EACESGSIFE	GLLPEDINIY
Mtr_7g032230	TDHGSPGSIG	IPGLLYANDF	VDALKKKHDA	KSYKKMVIYM	EACEAGSMFE	GLLPNDINIY
Mtr_7g079140	SGHGYPGLIG	MASLVYAKDL	VDALKKKHAS	NSYKKMVIYV	EACYSASLFE	GLLPNNISIY
Pda_gi672145835	SDHGGPGVLG	MPPFLYAADF	IDVLKKKHAS	GSYREMVIYV	EACESGSIFE	GLLPEGLNIY
Egu_gi743827934	SDHGGPGVLG	MPPFLYAADF	IDVLKKKHAS	GGYKEMVIYV	EACESGSIFE	GLMPEDLNIY
Mac_gi695022010	SDHGGPGVLG	MPPYLYAVDF	IEVLKKKHAM	NSYKEMVIYV	EACESGSIFE	GLMPKDLNIY
Mac_gi695070617	SDHGGPGVLG	MPPFLYAADF	IEVLKMKHAS	NGYKEMVIYV	EACESGSIFE	GLMPENLDIY
Atr_262_1	SDHGGPGVLG	MPPFLYANDL	MEVLKKKHKS	KGYKEMVIYV	EACESGSIFE	GLMTEDLNIY
Atr_262_2	SDHGGPGVLG	MPPFLYANDL	MEVLKKKHKS	KGYKEMVIYV	EACESGSIFE	GLMTEDLNIY
Atr_36_100	SDHGGAGVLG	MPPYLYADDL	VNVLKKKHVS	GTYKSLVFYL	EACESGSIFE	GLLPEGLNIY
Asp_gi356463708	SDHGGPGVLG	MPPYLYAADF	IKVLQEKHAS	NTYAKMVIYV	EACESGSIFE	GLMPADLNIY
Ata_gi356463714	SDHGGPGVLG	MPPYLYAADF	IKVLQEKHAS	NTYAKMVIYV	EACESGSIFE	GLMPADLNIY
Ata_gi475545141	SDHGGPGVLG	MPPYLYGDDL	VDVLKKKHAA	GTYKSLVFYL	EACESGSIFE	GLLPNDIGVY
Aly_gi297825691	SDHGGPGVLG	MPPYLYANDL	NDVLKKKHAS	GTYKSLVFYL	EACESGSIFE	GLLPEGLNIY
Aly_gi297798670	SDHGGPGVLG	MPPYLYANDL	NDVLKKKHAS	GTYKSLVFYL	EACESGSIFE	GLLPEGLNIY
Ath_gi334185489	ADHGAPGLIA	MPDEVMAKDF	NEVLEKMHKR	KKYNKMVIYV	EACESGSMFE	GILKKNLNIY
Ath_gi6630462	ADHGGPGVLG	MPPHIYAADF	IETLKKKHAS	GTYKEMVIYV	EACESGSIFE	GIMPKDLNIY
Ath_gi15233996	SDHGGPGVLG	MPPYLYANDL	NDVLKKKHAL	GTYKSLVFYL	EACESGSIFE	GLLPEGLNIY
Ath_gi15225226	SDHGGPGVLG	MPPNLYANDL	NDVLKKKYAS	GTYKSLVFYL	EACESGSIFE	GLLPEGLNIY
Adi_gi347326466	SDHGGPGILG	MPPYLYANDL	NEVLKKKHAS	GGYKSLVFYL	EACESGSIFE	GLLPEDINIY
Bdi_gi357136769	SDHGGPGVLG	MPPYLYAGDF	IKILQQKHAS	NTYAKMVIYV	EACESGSIFE	GLMPADLNIY
Bdi_gi357135238	SDHGGPGVLG	MPPYLYGDDL	VDVLKKKHAA	GTYKSLVFYL	EACESGSIFE	GLLPNDIGIY
Cen_gi1346432	SDHGGPGVLG	MPPFVYAMDF	IDVLKKKHAS	GGYKEMVIYI	EACESGSIFE	GIMPKDLNIY
Cru_gi565477221	SDHGGPGVLG	MPPYLYAKDL	NDVLKKKHAS	GTYKSLVFYL	EACESGSIFE	GLLPEGLNIY
Cru_gi565489031	ADHGGPGVLG	MPPHIYAADF	IETLKKKHAS	GTYKEMVIYV	EACESGSIFE	GIMPKDLNIY
Car_gi502175795	SDHGGPGVLG	MPPYLYAMDF	INVLKKKHAS	RGYKKMVIYV	EACESGSIFE	GIMPNNLNIY
Car_gi502132679	SDHGGPGVLG	MPPYMYASDL	IEVLKKKHAS	GTYKSLVFYL	EACESGSIFE	GLLPEGLNIY
Ccl_gi567895782	SDHGGPGVLG	MPPYVYAMEF	IDVLKKKHAA	KSYKGMVIYV	EACESGSIFE	GVMPKDLDIY
Ccl_gi568846845	SDHGGPGVLG	MPPYVYAMEF	IDVLKKKHAA	KSYKEMVIYV	EACESGSIFE	GVMPKDLDIY
Csa_gi449459758	SDHGGPGVLG	MPPFVYAMDF	IEVLKKKHAA	KGYKEMVIYV	EACESGSIFE	GILPKDLNIY
Esa_gi567127595	SDHGGPGVLG	MPPHLYAADF	IETLKKKHAA	GTYREMVIYV	EACESGSVFE	GIMPKDLNIY
Esa_gi567217560	SDHGGPGVLG	MPPYLYANDL	NDVLKKKHAS	GTYKSLVFYL	EACESGSIFE	GLLEEGLNIY
Fve_gi470125355	SDHGGPGVLG	MPPFLYAMDF	INVLKKKHAS	GSYKEMVIYV	EACESGSIFE	GIMPSDLNIY
Gau_gi527209183	SDHGGPGVLG	MPPFVYGKDL	IEALKKKHAS	GTYEEMMIYV	ESCESGSVFE	GIMPTDLNVY
Gma_gi351724961	TDHGGPGVLG	MPPYLYADDL	IEVLKKKHAS	GTYKNLVFYL	EACESGSIFE	GLLPEDINIY
Gma_gi356564077	TDHGGPGVLG	MPPYLYADDL	IEVLKKKHAS	GTYKNLVFYL	EACESGSIFE	GLLPEDINIY
Gma_gi571453325	SDHGGPGVLG	MPPYLYAMDF	IEVLKKKHAS	GGYKKM-IYV	EACESGSMFE	GIMPKDLQIY
Gma_gi351720847	SDHGGPGILG	MPPYLYAMDF	IDVLKKKHAS	GSYKEMVIYV	EACESGSVFE	GIMPKDLNIY
Hvu_gi194352742	SDHGGPGVLG	MPPYLYGDDL	VDVLKKKHAA	GTYKSLVFYL	EACESGSIFE	GLLPNDIGVY
Hvu_gi194352740	TDHGGAGSLG	MPPFVYAGDF	IKVLRQKHAS	KSYSKMVVYV	EACESGSIFE	GLMPRDHNIY
Hvu_gi194352736	SDHGGPGVLG	MPPYLYAADF	IKVLQEKHAS	NTYAKMVIYV	EACESGSIFE	GLMPADLNIY
Hvu_gi313660970	TDHGGAGSLG	MPPFVYAGDF	IKVLRQKHAS	KSYSKMVVYV	EACESGSIFE	GLMPQDHNIY
Hvu_gi313660968	ADHGGPGVLG	MPPYLYAGDF	IRVLREKHAS	KSYSKMIIYV	EACESGSIFE	GLLPEDLNIY
Msi_gi443501900	TDHGGPGILG	MPPYIYANDL	IEVLKKKHAA	GTYKSLVFYL	EACESGSIFE	GLLPEGLNIF
Mtr_gi357476961	SDHGGPGVLG	MPPYVYAMDF	IDVLKKKHAS	GGYKKMVVYI	EACESGSMFE	GVMPKDLNVY
Mtr_gi217074670	SDHGGPGVLG	MPPYVYAMDF	IDVLKKKHAS	GGYKKMVVYI	EACESGSMFE	GVMPKDLNVY
Mtr_gi357465673	SDHGGPGVLG	MPPFMYATDL	IEVLKKKHAS	ETYKSLVFYL	EACESGSIFE	GLLPEGLNIY
Mno_gi587864848	SDHGGPGVLG	MPPFLYAMDF	VEVLKKKHAS	GTYKEMIIYV	EACESGSIFE	GIMPRDMNIY
Nta_gi40809676	SDHGGPGVLG	MPPFLYAKDF	IEVLKKKHAA	GTYKEMVLYI	EACESGSVFE	GMMPEDLNIY
Obr_gi573916715	SDHGGPGVLG	MPPYLYGDDL	VDVLKKKHAA	GTYKSLVFYL	EACESGSIFE	GLLPDDINVY
Obr_gi573919988	SDHGGPGVLG	MPPYLYAADF	MKVLQEKHVS	NTYAKMVIYV	EACESGSIFE	GLMPEDLNVY
Osa_gi26006020	SDHGGPGVLG	MPPYLYAADF	MKVLQEKHAS	NTYAKMVIYV	EACESGSIFE	GLMPEDLNIY
Osa_gi115437636	SDHGGPGVLG	MPPYLYGDDL	VDVLKKKHAA	GTYKSLVFYL	EACESGSIFE	GLLPNGINVY
Osa_gi115465809	ADHGGPGVLS	MPEYLYADDL	VKALKKKHAG	GGYKSLVVYV	EACESGSIFE	GLLPSDISVY
Osa_gi6907094	SDHGSPGYVC	MPGNLHANDL	SQALKNKNAA	GAYKNLVVYV	EACESGSMFE	GLLPSNIGVY
Osa_gi38567871	SDHGGPGVLG	MPPYLYAGDF	IKVLQKKHAS	NSYSKMVIYV	EACESGSIFE	GLMPENLNIY
Prh_gi310771866	SDHGGPGVLG	MPPYLYADDL	VNVLKQKHAL	GAYKSLVFYL	EACESGSIFE	GILPKGLNIY
Pvu_gi48429177	SDHGGPGVLG	SPPYIYASDL	NEVLKKKHAS	GTYKNLVFYL	EACESGSIFE	GLLPEDINVY
Pvu_gi561009896	SDHGGPGVLG	MPPYMYASDL	IEVLKKKHAS	ETYKSLVFYL	EACESGSIFE	GLLPEGMNIY
Pvu_gi561028978	SDHGGPGVLG	MPPYLYAMDF	IDVLKKKHAS	GGYKEMVIYV	EACESGSIFE	GIMPKDLNIY
Ppa_gi168013224	SDHGGPGVLG	MPPNLYADDL	LKTFKKMHEA	KTYKEMVVYI	EACESGSIFQ	GLLPKDLNIY
Ppa_gi168033758	SDHGGPGVLG	MPPNLYADDF	VGILKKKAAA	GTFKELVIYL	EACESGSIFE	GLLPEGLNIY
Ppa_gi168005016	TDHGGAGVLG	MPPNLYADDF	VDTLKKKAAA	GTFKELVIYL	EACESGSIFE	GLLPEGLNIY
Ppa_gi168065024	ADHGGAGVLG	MPPILYADEF	VDTLKKKAAA	GTFKKMVIYV	EACESGSIFD	GLLPTGLNIY
Pab_MA_60392g0010	SDHGGPGVLG	MPPYLYAHDL	VEVLKKKHAA	GAYKEMVIYI	EACESGSIFE	GLLPKGLNIY
Pab_MA_86205g0010	TDHGGPGVLG	MPHMLYAKDL	IDVLKKKHAA	DTYKQMVIYV	EACESGSIFE	GLLPEGMNIY
Psi_gi148910236	SDHGGPGVLG	MPPYLYANDF	VQVLKKKHDA	GSYREMVIYV	EACESGSIFE	GLLPTDLNIY
Psi_gi116789977	TDHGGPGVLG	MPHMLYAKDL	VDVLKKKHAA	DTYKQMVIYV	EACESGSIFE	GLLPEGMNIY
Pta_000008627	SDHGGPGVLG	MPPYLYANDF	IQVLKQKHAA	GSYREMVIYI	EACESGSIFE	GLLPTDMNIY
Pta_000069534	SDHGGPGVLG	MPPYLYAHDL	VEVLKKKHAA	NAYKEMVIYI	EACESGSIFE	GLLPKGLKIY
Pta_000008629	TDHGGPGVLG	MPPSLYANDL	IDVLKKKHAA	DSYKQMVIYV	EACESGSIFE	GLLPEGMNIY
Ptr_006G232900	TDHGGPGVLG	MPPYLYADDL	IDVLKKKHAS	GTYKSLVFYL	EACESGSIFE	GLLPQGLNIY
Ptr_gi566162122	SDHGGPGVLG	MPPFLYAMDF	IEVLKKKHAS	GSYKEMVMYI	EACESGSIFE	GIMPKDINIY
Ptr_001G119800	SDHGGPGVLG	MPPFLYAMDF	IEVLKKKHAS	GSYKEMVMYI	EACESGSIFE	GIMPKDLNIY
Ptr_224141591	TDHGGPGVLG	MPPYLYADDL	IDVLKKKHAS	GTYKSLVFYL	EACESGSIFE	GLLPQGLNIY
Ptr.008G003400	ADHGAPGLVG	MPKDLYAKDL	IQVLKKQQEA	NSYKSMVFYL	EACESGSMFE	GLLPSNWSIY
Ppe_gi462419182	SDHGGPGVLG	MPPYIYANDL	IEVLKKKHAA	GTYKSLVFYL	EACESGSIFE	GLLPEGLNIF
Ppe_gi462405686	SDHGGPGVLG	MPPFLYAMDF	IQVLKKKHAS	GSYKEMVIYV	EACESGSIFE	GIMPSDLNIY

```
Rco_gi255537021      SDHGGPGVLG MPPYLYAMDF IEVLKKKHAA GGYKKMVIYV EACESGSIFE GIMPKDVDIY
Rco_gi255550848      TDHGGPGVLG MPPYLYANDL IDVLKKKHAS GTYKSLVFYL EACESGSIFE GLLPEGLNIY
Shy_gi373254763      SDHGGPGVLG MPPYLYGDDL VDVLKKKHAA GTYKSLVFYL EACESGSIFE GLLPDDINVY
Sof_gi93139442       SDHGGPGVLG MPPYLYGDDL VDVLKKKHAA GSYKSLVFYL EACESGSIFE GLLPDDINVY
Smo_SM00084G00480    SDHGGPGVLG MPSNLYANDL IDVIKKKHAS GGYREMVIYI EACESGSMVE GLLPLGLGLY
Smo_SM00000G12900    SDHGGPGVLG MPPYLYAVDL VTTLQDMHDN NKYKEMVLYI EACESGSIFE GLLPKNLNIF
Sin_gi9622221        SDHGGPGVLG MPPYLYANDF IEVLKKKHAS GTYKEMVIYV EACESGSVFE GLMPDDLDIY
Sit_gi514715547      SDHGGPGVLG MPPYIYAGDF MKVLREKHAS NSYAKMVIYV EACESGSIFE GLMPEDLNIY
Sit_gi514802965      SDHGGPGVLG MPPYLYAGDF IKVLKKKHAS NSYSKMVIYV EACESGSIFE GLMPQDLNIY
Sly_gi460400421      SDHGGPGVLG MPPYLYGKDL IEVLKKKYAA RTYKEMVLYI EACESGSVFE GLMPENLNIY
Stu_gi565378091      SDHGGPGVLG MPPYLYGKDL IEVLKKKYAA GTYKEMVLYI EACESGSVFE GLMPENLNIY
Sbi_gi242063120      SDHGGPGVLG MPPYLYAGDF MKVLREKHAS NSYAKMVIYI EACESGSIFE GLMPEDLNIY
Sbi_gi242076602      SDHGGPGVLG MPPYLYAGDF IKVLKKKHAC NSYSKMVIYV EACESGSIFE GLMPEDLNIY
Sbi_gi242053313      SDHGGPGVLG MPPYLYGDDL VDVLKKKHAA GTYKSLVFYL EACESGSIFE GLLPDDINVY
Tha_gi312281859      SDHGGPGVLG MPPYLYANDL NDVLKKKHAS GTYKSLVFYL EACESGSIFE GLLEEGLNIY
Tca_gi590677122      SDHGGPGVLG MPPFLYAMDF LDVLKKKHAA GSYKEMVIYV EACESGSVFE GIMPKDLNIY
Tca_gi590573851      TDHGGPGVLG MPPYLYADDL IEVLKKKHAS GTYKSLVFYL EACESGSIFE GLLPEGLNIY
Tmo_gi356463700      SDHGGPGVLG MPPYLYAADF IKVLQEKHAS NTYAKMVIYV EACESGSIFE GLMPADLNIY
Tmo_gi356463706      SDHGGPGVLG MPPYLYGDDL VDVLKKKHAA GTYKSLVFYL EACESGSIFE GLLPNDIGVY
Tur_gi474124510      TDHGGAGSLG MPPFVYAGDF IKVLRQKHAS KSYSKMIIYV EACESGSIFE GLMPQDHNIY
Vna_gi2414681        SDHGGPGVLG MPPYVYAMDF IDVLKKKHAS GGYKKMVIYV EACESGSIFE GIMPKDINVY
Vsa_gi3452551        SDHGGPGVLG MPPYVYAMDF IDVLKKKHAS RGYQQMVIYV EACESGSVFQ GIMPKDIDVY
Vvi_gi442540375      SDHGGQGVLG MPPFLYAKDF IDVLKMKHAS GSYKEMVLYV EACESGSIFE GLMPDDLNIY
Vvi_gi296085092      SDHGGQGVLG MPPFLYAKDF IDVLKMKHAS GSYKEMVLYV EACESGSIFE GLMPDDLNIY
Vvi_VV04G07860       SDHGGPGVLG MPPYLYANDL IEVLKKKHAS GTYNSLVFYL EACESGSIFE GLLPEGLNIY
Vvi_VV09G09760       ADHGATGIIG MT-LIYAKDL IDVLKKKHEA KAYKTMVIYI EACEAGSMFQ GLLPNNWDIY
Zma_gi413937993      SDHGGPGVLG MPPYLYAGDF MKVLREKHAS NSYAKMVIYI EACESGSIFE GLMPEDLNIY
Zma_gi414586098      SDHGGPGVLG MPPYLYAGDF IKVLKKKHAS NSYSKMVIYV EACESGSIFE GLMPEDLNIY
Zma_gi226493414      SDHGGPGVLG MPPYLYAGDF IKVLKKKHAS NSYSKMVIYV EACESGSIFE GLMPEDLNIY
Zma_gi195624628      SDHGGPGVLG MPPYLYGDDL VDVLKKKHAA GTYKSLVFYL EACESGSIFE GLLPNDINVY
Zma_ZM08G11940       SDHGGPGVLG MPDYLYAKDL VDALRKKHAA GGYRSLVFYL EACESGSIFE GLLPPDIAVY
Zma_gi6634705        SDHGGPGVLG MPPYLYGDDL VDVLKKKHAA GTYKSLVFYL EACESGSIFE GLLPNDINVY
```

```
                  181
Mtr_1g016590      ATTASNAVES SWGTYPPPPP EYSTCLGDLY SIAWMEDSES LHQQYVKDRT INGY---YGS
Mtr_7g032230      VTTASNKSEN SYGFYLPPPP EYDICLGDLY SISWMEDSEI LKEQYVRQRT LL-------S
Mtr_7g079140      VTTSANAREL GYGFYNLSST EYTTCLGDTF GISWMEDSET LQQQYVRDRT I-------TS
Pda_gi672145835   VTTASNAEES SWGTYPAPPP EYITCLGDLY SVAWMEDSET IEKQYVKERT SNYNTYSAGS
Egu_gi743827934   VTTASNAEES SWGTYPSPPP EFITCLGDLY SVAWMEDSET IQKQYVKERT SNYNTYNTGS
Mac_gi695022010   VTTASNAEES SWGTYPPPPP EYITCLGDLY SVAWMEDSET VSKQYVKVRT SNYNTYSVGS
Mac_gi695070617   VTTASNAVES SWGTYPPPPR EFTTCLGDLY SVAWMEDSET VGKQFVKMRT SNHDTYNTGS
Atr_262_1         VTTASNAQES SWGTYPPPPP EFMTCLGDLY SVAWMEDSET IQKQYVKSRT SNYNTYTAGS
Atr_262_2         VTTASNAQES SWGTYPPPPP EFMTCLGDLY SVAWMEDSET IQKQYVKSRT SNYNTYTAGS
Atr_36_100        ATTASNAVES SWGTYPDFPQ EYDTCLGDLY SVSWMEDSET LKQQYVKMRT SNFETYMFGS
Asp_gi356463708   VTTASNAEES SWGTYPSPPS EYITCLGDLY SISWMEDSET IKKQYVKKRT SDMNSYSAGS
Ata_gi356463714   VTTASNAEES SWGTYPSPPS EYITCLGDLY SISWMEDSET IKKQYVKKRT SDMNSYSAGS
Ata_gi475545141   ATTASNAEES SWGTYPSPPP EYDTCLGDLY SISWMEDRES LKQQYVKKRT AAQDSYSYGS
Aly_gi297825691   ATTASNAVES SWGTYPSPPS EYETCLGDLY SVAWMEDSET LHQQYVKKRT AGSGK-SFGS
Aly_gi297798670   ATTASNAEES SWGTYPSPPP EYETCLGDLY SVAWMEDSET LHQQYVKRRT APVG-YSYGS
Ath_gi334185489   AVTAANSKES SWGVYPPPPS EIGTCLGDTF SISWLEDSET LEQQYVKRRV GSDVPE--TS
Ath_gi6630462     VTTASNAQES SYGTYPSPPS EYITCLGDLY SVAWMEDSET IKQQYVKMRT SNYNTYSGGS
Ath_gi15233996    ATTASNAEES SWGTYPSPPP EYETCLGDLY SVAWMEDSET LHQQYVKRRT APVG-YSYGS
Ath_gi15225226    ATTASNAEES SWGTYPSPPS EYETCLGDLY SVAWIEDSET LHEQYVKKRT AGSGKS-YGS
Adi_gi347326466   ATTASNAVES SWGTYPSPPP EYSTCLGDLY SISWMEDSET LHQQYVKDRT LNGNAY-YGS
Bdi_gi357136769   VTTASNAEES SWGTYPSPPS EYITCLGDLY SVSWMEDSET IKKQYVKRRT SDLNSYSAGS
Bdi_gi357135238   ATTASNAEES SWGTYPSPPP EYDTCLGDLY SIAWMEDSES LKQQYVKKRT APENSYSYGS
Cen_gi1346432     VTTASNAQEN SFGTYPPPPE EYVTCLGDLY SVSWMEDSET VQQQYVRKRT SNSNSYRFGS
Cru_gi565477221   ATTASNAEES SWGTYPSPPS EYETCLGDLY SVAWMEDSES LHQQYVKKRT AGTGS-SYGS
Cru_gi565489031   VTTASNAQES SYGTYPSPPS EYITCLGDLY SVAWMEDSET IKQQYVKMRT SNYNTYSAGS
Car_gi502175795   VTTASNAQEN SWGTYPAPPP EFITCLGDLY SVAWMEDSET VKEQYVKERT SNSNNYALGS
Car_gi502132679   ATTAANADES SWGTYPSPPP EYETCLGDLY SVAWMEDSET LHQQYVKERT KNGNTL-YGS
Ccl_gi567895782   VTTASNAQES SFGTYPSPPP EYITCLGDLY SVAWMEDSET ISQQYVKERT SNFNNYNSGS
Ccl_gi568846845   VTTASNAQES SFGTYPSPPP EYITCLGDLY SVAWMEDSET INQQYVKERT SNFNNYNSGS
Csa_gi449459758   VTTASNAQES SFGTYPAPPP EYMTCLGDLY SVAWMEDSET IDQQYVKERT SNPNNLNTGS
Esa_gi567127595   VTTASNAQES SYGTYPSPPS EYITCLGDLY SVAWMEDSET IKQQYVKMRT SNYNSYSEGS
Esa_gi567217560   ATTASNAVES SWGTYPSLPP EYETCLGDLY SVSWMEDSET LRQQYVKRRT AGVGSA-YGS
Fve_gi470125355   VATASNAEEN SFGTYPPPPP EYITCLGDLY SVAWMEDSET IKEQYVKERT SNFNNYNIGS
Gau_gi527209183   VTTASNAEES SWGTYPAPPP EYMTCLGDLY SVAWMEDSET VKEQYVKERT SNFNSYNAGS
Gma_gi351724961   ATTASNAEES SWGTYPSPPP EYTTCLGDLY SVAWMEDSET LHQQYVKERT ISGDSY-YGS
Gma_gi356564077   ATTASNAEES SWGTYPSPPP EYSTCLGDLY SVAWMEDSET LHQQYVKERT ISGDSY-YGS
Gma_gi571453325   VTTASNAQEN SWGTYPSPPP EYITCLGDLY SVAWMEDSES VKQQYVKQRT SNFNNYAMGS
Gma_gi351720847   VTTASNAQEN SWGTYPSPPP EYITCLGDLY SVAWMEDSES VKQQYVKQRT SNFNNYAMGS
Hvu_gi194352742   ATTASNAEES SWGTYPSPPP EYDTCLGDLY SISWMEDSES LKQQYVKKRT AAQDSYSYGS
Hvu_gi194352740   VTTAANAEES SWAAYIPPPS EYYTCLGDAY SVSWMEDSET IKQQYVKART APRNKSSIGS
Hvu_gi194352736   VTTASNAEES SWGTYPSPPS EYITCLGDLY SISWMEDSET IKKQYVKKRT SDMNSYSAGS
Hvu_gi313660970   VTTAANAEES SWAAYIPPPS EYYTCLGDAY SVSWMEDSET IKQQYVKART APGNKSSIGS
Hvu_gi313660968   VTTASNAVEN SWGAYSSPPT EYDTCIGDIY SVSWMEDSET LKQQYVKSRT SKSKEFDKGS
Msi_gi443501900   ATTASNAEES SWGTYPSPPP EYDTCLGDLY SVAWMEDSET LHQQYVKTRT ANDNS-GFGS
Mtr_gi357476961   VTTASNAQES SWGTYPAPPP EYITCLGDLY SVAWMEDSET VKQQYVKERT SNYNNYALGS
Mtr_gi217074670   VTTASNAQES SWGTYPAPPP EYITCLGDLY SVAWF----- ---------- ----------
Mtr_gi357465673   ATTAANAEES SWGTYPSPPP EYETCLGDLY SVAWMEDSET LHQQYVKERT SNGNSI-YGS
Mno_gi587864848   VTTASNAQEN SWGTYPSPPP EYITCLGDLY SVAWMEDSET IKQQYVKKRT ANANDFTAGS
Nta_gi40809676    VTTASNAEES SWGTYPPPPP EYITCLGDLY SVAWMEDSET IKQQYVKERT SNFNNYNAGS
Obr_gi573916715   ATTASNADES SWGTYPSPPP EYDTCLGDLY SVAWMEDCES LRQQYVKERT SVQHTYDSGS
Obr_gi573919988   VTTASNAEES SWGTYPSPPA EYITCLGDLY SVSWMEDSES IKEQYVKKRT SDMNSYGAGS
Osa_gi26006020    VTTASNAEES SWGTYPSPPS EYITCLGDLY SVSWMEDSES IKKQYVKKRT SDMNSYGAGS
Osa_gi115437636   ATTASNADES SWGTYPSPPP EYDTCLGDLY SVAWMEDSES LKQQYVKERT SVQHTYYSGS
Osa_gi115465809   ATTASNAEES SWGTYDAPAA EFDTCLGDLY SVAWMEDAET LRQQYVKNRT SDEGTYTLGS
Osa_gi6907094     AMTASNATEN SWATY---TP EYNTCLGDLF SVAWMEDAET LGQLYVAKRT N-------LS
Osa_gi38567871    VTTASNAVEN SWGTYPSPPP EYITCLGDMY SVAWMEDSET IEDQYVKKRT SNANKLNEGS
Prh_gi310771866   ATTASNAEES SWGTYPSPPS EYETCLGDLY SVAWMEDSET LKQQYVKERT QNANSA-YGS
Pvu_gi48429177    ATTASNADES SWGTYPSPPP EYSTCLGDLY SVAWMEDSET LHQQYVKERT ISGGLY-YGS
Pvu_gi561009896   ATTAANAEES SWGTYPSPPP EYETCLGDLY SVAWMEDSET LHQQFVKQRT INGNSA-YGS
Pvu_gi561028978   VTTASNAQEN SWGTYPPPPP EYITCLGDLY SVAWMEDSES VEQQYVKQRT SNFEAYAMGS
Ppa_gi168013224   ATTAANAEES SWGTYPAPLE EFDTCLGDLY SVAWMEDTET LRDQYVKSRT SNHNTYKSGS
Ppa_gi168033758   VTTASNAVES SWGTYPSPPS EYGTCLGDLY SVAWMEDTET LEDQYVKSRT SNHNTYRSGS
Ppa_gi168005016   VTTASNAEES SWGTYPPPPP EYDTCLGDLY SVAWMEDTET LEDQYVKSRT SNHNTYRTGS
Ppa_gi168065024   VTTASDPDEN SWGTYPPPPP EFGTCLGDLY SVSWMEDAET LNDQYVKSRT SDNDTYMTGS
Pab_MA_60392g0010 VTTAANGEES SWGTYPPPPP EYETCLGDLY SVAWMEDSET IKQQYVKFRT SDHNTYQAGS
Pab_MA_86205g0010 VTTASNAEES SWGTYPSPPL EYDTCLGDLY SVAWMEDSET LKQQYVKERT SNHQTYGMGS
Psi_gi148910236   VTTASNAEEN SWGTYPPPPP EYDTCLGDLY SVAWMEDSET LLQQYVKLRT SNHNTYMSGS
Psi_gi116789977   VTTASNAEES SWGTYPSPPL EYDTCLGDLY SVAWMEDSET LKQQYVKERT SNHQTYGMGS
Pta_000008627     ATTASNAEEN SWGTYPPPPP EYDTCLGDLY SVAWMEDSET LLQQYVKRRT SNHNTYMTGS
Pta_000069534     VTTAANGEES SWGTYPPPPP EYETCLGDLY SIAWMEDSET IKQQYVKFRT SDHNTYRAGS
Pta_000008629     VTTASNAEES SWGTYPSPPL EYDTCLGDLY SVAWMEDSET LDQQYVKERT SNHHTYGMGS
Ptr_006G232900    ATTASNAEES SWGTYPSPPP EYETCLGDLY SVAWMEDSET LHQQYVKRRT S-YDNSPYGS
Ptr_gi566162122   VTTASNAEEN SWGTYPSPPP EYFTCLGDLY SVSWMEDSET IEQQYVKERT SNYNTFTSGS
Ptr_001G119800    ---------- ---------- -YVTCLGDLY SVAWMEDSET IKQQYVKERT SNYNAFTSGS
Ptr_224141591     ATTASNAEES SWGTYPSPPP EYETCLGDLY SVAWMEDSET LHQQYVKRRT SNDNSP-YGS
Ptr.008G003400    AITAANGEES SYGIYPAPPP EFLTCLGDVF SISWMEDSET LQQQYVRRRT --GFDYEDRS
Ppe_gi462419182   ATTASNAEES SWGTYPSPPP EYETCLGDLY SVAWMEDSET LHQQYVKTRT ANDNS-GFGS
Ppe_gi462405686   VTTASNAQEN SFGTYPSPPP EYITCLGDLY SVAWMEDSET IKQQYVKKRT SNSNNYDVGS
```

Rco_gi255537021	VTTASNAQES	SWGTYPSPPP	EFTTCLGDLY	SVAWMEDSET	VKQQYVKART	SNYNTYAAGS
Rco_gi255550848	ATTASNAEES	SWGTYPSPPP	EYETCLGDLY	SIAWMEDSET	LHQQYVKRRT	SNGNSA-YGS
Shy_gi373254763	ATTASNAEES	SWGTYPSPPP	EYDTCLGDLY	SVSWMEDSES	LKQQYVKDRT	AAQDTFSYGS
Sof_gi93139442	ATTASNAEES	SWGTYPSPPP	EYDTCLGDLY	SVSWMEDSES	LKQQYVKDRT	AAQDTFSYGS
Smo_SM00084G00480	VTTASNAIES	SWGTYPSAPP	EYDTCLGDLY	SVAWMEDSET	LLQQYVKDRT	SNHNTYEAGS
Smo_SM00000G12900	VTTASNAVES	SWGTYPSPPP	EYDTCIGDLY	SVAWMEDSER	LKDQYVKART	SDANTYRMGS
Sin_gi9622221	VTTASNAEES	SWGTYPPPPP	EYITCLGDLY	SVAWMEDSET	VEQQYVKERT	SNFNTYNAGS
Sit_gi514715547	VTTASNAEES	SWGTYPSPPS	EYITCLGDLY	SVSWMEDSET	IKEQYVKERT	SDSNSYGAGS
Sit_gi514802965	VTTASNPVEN	SWGTYPSPPP	EYITCLGDLY	SVSWMEDSET	IKDQYVKTRT	SNLKKYKEGS
Sly_gi460400421	VMTASNAEES	SWGTYPPPPS	EYITCLGDLY	SVAWMEDSET	IKQQYVKERT	SNSNNYNAGS
Stu_gi565378091	VTTASNAEES	SWGTYPPPPS	EYITCLGDLY	SVAWMEDSET	IKQQYVKERT	SNSNNYNAGS
Sbi_gi242063120	VTTASNAEES	SWGTYPSPPS	EYITCLGDLY	SVSWMEDSET	IKEQYVKERT	SDSNSYGAGS
Sbi_gi242076602	VTTASNPVEN	SWGTYPSPPP	EYITCLGDLY	SVSWMEDSET	IKDQYVKTRT	SNSNKYKEGS
Sbi_gi242053313	ATTASNAEES	SWGTYPSPPP	EYDTCLGDLY	SVSWMEDSES	LKQQYVKDRT	AVQDTFSYGS
Tha_gi312281859	ATTASNAVES	SWGTYPSLPP	EYETCLGDLY	SVSWMEDSET	LRQQYVKRRT	AGVGSA-YGS
Tca_gi590677122	VTTASNAQES	SWGTYPSPPP	EYTTCLGDLY	SVAWMEDSET	VKQQYVRERT	SNFNSYTLGS
Tca_gi590573851	ATTASNAEES	SWGTYPSPPP	EYETCLGDLY	SVAWMEDSET	LHQQYVKRRT	INGNSA-YGS
Tmo_gi356463700	VTTASNAEES	SWGTYPSPPS	EYITCLGDLY	SISWMEDSET	IKKQYVKKRT	SDMNSYSAGS
Tmo_gi356463706	ATTASNAEES	SWGTYPSPPP	EYDTCLGDLY	SISWMEDSES	LKQQYVKKRT	AAQDSYSYGS
Tur_gi474124510	VTTAANAEES	SWAAYVPPPS	EYKTCLGDAY	SVSWMEDSES	IKQQYVKART	APPNESSIGS
Vna_gi2414681	VTTASNAQEN	SWGTYPAPPP	EYITCLGDLY	SVAWMEDSET	VKQQYVRERT	SNYKNYPLGS
Vsa_gi3452551	VTTASNAEES	SWGTYPASPP	EYITCLGDLY	SVAWMEDSET	LKQQFVKERT	LNNNNYGLGS
Vvi_gi442540375	VTTASGPDEE	SWGTYPAPPP	EYITCLGDLL	SVAWLEDSQT	IEDQYVKVRT	SNHNTYSVGS
Vvi_gi296085092	VTTASGPDEE	SWGTYPAPPP	EYITCLGDLF	SVAWLEDSQT	IEDQYVKVRT	SNHNTYSVGS
Vvi_VV04G07860	ATTAANAEES	SWGTYPSPPP	EYETCLGDLY	SVAWMEDSET	LRQQYVKKRT	ANDNSV-YGS
Vvi_VV09G09760	ATTAANAEEN	SYGTYPSAPS	EYDTCLGDTY	SVAWLEDSET	LEKQYIRRRV	FTQD-LDFNS
Zma_gi413937993	VTTASNAEES	SWGTYPPPPS	EYITCLGDLY	SVSWMEDSET	IKEQYVKKRT	SDFNSYGAGS
Zma_gi414586098	VTTASNPVEN	SWGTYPSPPP	EYITCLGDLY	SVSWMEDSET	IKDQYVKTRT	SNSNKYKEGS
Zma_gi226493414	VTTASNPVEN	SWGTYPSPPP	EYITCLGDLY	SVSWMEDSET	IKDQYVKTRT	SNSNKYKEGS
Zma_gi195624628	ATTASNAEES	SWGTYPSPPP	EYDTCLGDLY	SVAWMEDSES	LKQQYVKDRT	AVHDTFSYGS
Zma_ZM08G11940	ATTAANAEES	SWGTYPGPPP	EFDTCLGDLY	SVAWMEDSET	LRQQYVKDRT	SAHGTYSLGS
Zma_gi6634705	ATTASNAEES	SWGTYPSPPP	EYDTCLGDLY	SVAWMEDSES	LKQQYVKDRT	AVQDTFSYGS

```
                   241
Mtr_1g016590       HVMEYGDNHL FLYLGTNP-- ---------- --Y------- ---------- ----------
Mtr_7g032230       HVLQYGDDTL ITYIGADPFG QRDAHLIYLK TKLGRASSGS EDKLKAQKVE IARRKHVDNN
Mtr_7g079140       HVTQLGDDFL DTYIGSAPVN QDDAYLLHLK LKLEKAVDGS KDKLKAQNAE IAHRKHVDHN
Pda_gi672145835    HVMEYGDDKL YLFQGFDPIN QRDADLLFLW KRYEQLDARS EKKTQVLKET MMHRLHLDNS
Egu_gi743827934    HVMEYGDDKL YLFQGFDPIN QRDADLLFLW KRYEQFDEGS KKKTEALRET LVHRLHLDNS
Mac_gi695022010    HVMEYGDEKL YLYQGFDPIN QRDADLLFLW KRYERLAESS EDKRRTVMET MMHRTHLDRS
Mac_gi695070617    HVMEYGDDVL SLYQGFEPIN QRDADLLFMW KMYEQSDERS SKKKEILEKT LMHRVHLDSS
Atr_262_1          HVMEYGDERL YLYQGFDPVN QRDADLLFLW QRYKRSTEGS EEKVAFRNEK MAHREHLDKS
Atr_262_2          HVMEYGDERL YLYQGFDPVN QRDADLLFLW QRYERSTEGS EEKVAIRNEK MAHREHLDKS
Atr_36_100         HVMQYGDEQL VLYMGSNPVN QRDADLVYFW NKYRKSPVGS IKKRNAQKDV MAHRLHLDNS
Asp_gi356463708    HVMEYGDEKL YLYQGFNPIN QRDADLLFLW RRYELLHEKS KEKANVLRET VAHRKHLDSS
Ata_gi356463714    HVMEYGDEKL YLYQGFNPIN QRDADLLFLW RRYELLHGKS KEKANVLTET VAHRKHLDNS
Ata_gi475545141    HVMQYGSEHL FSYIGSNPVN QRDADLVYFW QKYRKLAESS PEKNDARKEM MGHRSHIDNS
Aly_gi297825691    HVMEFGDEKL VLYMGTNPTN QRDADLVHFW DKYRKAPEGS ARKVEAQKEA MSHRLHVDNS
Aly_gi297798670    HVMQYGDDNL DLYMGTNPTN QRDADLVHFW EKYRKAPEGS ARKTEAQKEA MSHRLHVDNS
Ath_gi334185489    HVCRFGTDYL SSYIGRNPVN PRDIPLLYLQ RKIQKAPMGS LESKEAQKDE KNHRKQIDQS
Ath_gi6630462      HVMEYGNEKL YLYQGFDPVN QRDADLLFLW HMYRTSEDGS RKKDDTLKET TRHRKHLDAS
Ath_gi15233996     HVMQYGDDNL DLYMGTNPTN QRDADLVHFW EKYRKAPEGS ARKTEAQKEA MSHRLHIDNS
Ath_gi15225226     HVMEFGDEKL VLFMGTNPTN QRDADLVHFW HKYQKAPEGS ARKVEAQKEA MSHRLHVDNS
Adi_gi347326466    HAMQYGDNLL FQYLGTNPVN QRDADLIHFW EKFRKAPEGS SSKITAQKEV MSHRMHIDNS
Bdi_gi357136769    HVMEYGDEKL YLYQGFNPIN QRDADLLFLW RRYEMLHEKS KEKVKVLRET VMHRKHLDNS
Bdi_gi357135238    HVMQYGSEHL FLYIGSNPVN QRDADLVYFW QKYRKLAESS PAKNDARKEM MAHRSHVDNS
Cen_gi1346432      HVMQYGDEKL YLYHGFDPVN QRDAELLFMW QMYQRSNHQP EKKTHILEET VKHRNHLDGS
Cru_gi565477221    HVLEFGDEKL VLYMGTNPTN QRDADLVHFW DKYRKAPEGS ARKVEAQKEA MSHRLHVDNS
Cru_gi565489031    HVMEYGNEKL YLYQGFDPVN QRDADLLFLW HMYRRSEDGS RKKDDTLKET TRHRKHLDAS
Car_gi502175795    HVMQYGDEKL YLYHGFNPVN QRDAELFFMW QLYKRLDHEA EKKRDILEET VKHRNHLDGS
Car_gi502132679    HVMQYGDNSL FLYLGTNPVN QRDADLVHFW DKFRKAPQGS PRKAAAEKEA MSHRMHIDDS
Ccl_gi567895782    HVMEYGNEKL YLYQGFDPVN QRDADLLFMW HMYKNAAEGS EKKSEMLKET MRHRKHLDAS
Ccl_gi568846845    HVMEYGNEKL YLYQGFDPVN QRDADLLFMW HMYKNAAEGS EKKSEMLKET MRHRKHLDAS
Csa_gi449459758    HVMEYGNERL YLYQGFDPIN QRDADIFFLW QMYRKFEDGT NERAQVLEET VTHRTHLDGS
Esa_gi567127595    HVMEYGNEKL YLYQGFDPVN QRDADLLFLW HMYRTSEDGS -RKDEILKDQ MRHRKHLDAS
Esa_gi567217560    HVMQYGDDKL DLYMGTNPTN QRDADLVHFW DKYRKAPEGS TRKTEAQKEA MSHRLHVDNS
Fve_gi470125355    HVMQYGSEKL YLYLGFDPVN QRDAEIFFMW QLYKRSEHGS EKKREILKDT MNHRNHIDGS
Gau_gi527209183    HVMEYGNEKL CLYQGFDPVN QRDADLLFMW ERYKKLKDNS EEKSEHLKDA IAYRKHLDVS
Gma_gi351724961    HVMQYGDDVL FHYLGTDPVN QRDADLIHFW DKFRKAPEGS LRKNTAQKEA MSHRMHVDNS
Gma_gi356564077    HVMQYGDDVL FHYLGTDPVN QRDADLIHFW DKFRKAPEGS LRKNAAQKEA MSHRMHVDNS
Gma_gi571453325    HVMQYGDEKL YLYQGFDPVN QRDAELFFMW QMYQRSNHQP EKKTDILKET VKHRKHIDGS
Gma_gi351720847    HVMQYGDEKL YLYQGFDPVN QRDAELFLLW QMYQRSNHQS ENKTDILKET VKHRKHIDGS
Hvu_gi194352742    HVMQYGSEHL FSYIGSNPVN QRDADLVYFW QKYRKLAESS PAKNNARKEM MGHRSHIDSS
Hvu_gi194352740    HVMEYGDEML FLYQGFDPIN QRDADVLFMW KKYEQLNGGS EEKHRALRET VLHRKHLDSS
Hvu_gi194352736    HVMEYGDEKL YLYQGFNPIN QRDADLLFLW RRYELLHEKS KEKVNVLRET VTHRKHLDSS
Hvu_gi313660970    HVMEYGDEML FLYQGFDPIN QRDADVLFMW KKYEQLNGGS EEKHRALRET VLHRKHLDSS
Hvu_gi313660968    HVMEYGDEKL FHYQGFNPIN QRDADILFMW KRYEKLNGGS EEKLGVLRET VAHRKHLDSS
Msi_gi443501900    HVMQYGDNNL FVYMGTNPVN QRDADLLHFW HKYRKAPEGS ARKIQAQKEA MSHRMHIDQT
Mtr_gi357476961    HVMQYGDEKL YLYQGFDPVN QRDAEILFMW EMYKRLDHQT EKKREILEET VKHRNHLDGS
Mtr_gi217074670    ---------- ---------- ---------- ---------- ---------- ----------
Mtr_gi357465673    HVMQFGDDSL FLYLGSNPVN QRDADLIHFW DKFRKAPQGS PRKVAAQKEA MSHRMHIDES
Mno_gi587864848    HVMEYGNEKL YLYQGFDPVN QRDAELFFMW QMYKRTESRN -KKREILNEA TRHRTHLDKS
Nta_gi40809676     HVMEYGSEKV YLYQGFDPVN QRDADLLFLW ERYKELADNS LEKAKLRKDT MLHRKHLDGS
Obr_gi573916715    HVMQYGSHHL FLYMGSNPVN QRDADLVYFW QKYRKLAEGS PEKNEARKEM MAHRSHVDNS
Obr_gi573919988    HVMEYGDEKL YLYQGFDPVN QRDADLLFLW RRYELLHEKS EEKLKVLREA VTHRKHLDSS
Osa_gi26006020     HVMEYGDDKL YLYQGFDPVN QRDADLLFLW RRYELLHDKS EEKLKALRDT VMHRKLLDSS
Osa_gi115437636    HVMEYGSHHV FMYMGSNPVN QRDADLVYFW QKYRKLPESS PEKNEARKEM MAHRSHVDNS
Osa_gi115465809    HVMQYGDQSL DLYMDTSPVN QRDADLLYLW RKYRRAGEGT AEKVEAREQE MGRRSRVDRS
Osa_gi6907094      HVSRYGDQPV SLYLPPGPVN QRDAGLVYLW RKYYE----- EKSVEAWERE MERRSRVDSS
Osa_gi38567871     HVMEYGDEKL FLYQGFNPVN QRDADLLFMW KRYEQLNGVS EDKLRALRDT IAHRKHLDSS
Prh_gi310771866    HVMQYGDEDL FLYMGTNPVN QREADLIHFW QKYRKAPEGS QRKADAQKEV MAHRMHVDHS
Pvu_gi48429177     HVMQYGDDIL FHYLGTDPVN QRDADLVHFW DKFRKAPEGS PKKNEARKEV MSHRMHIDDS
Pvu_gi561009896    HVMQYGDNNL SLYLGTNPVN QRDADLVHLW DKYRKAPVGS SRKSVAQKEA MSHRMHIDDS
Pvu_gi561028978    HVMQYGDEKL YLYHGFDPVN QRDAELLFMW QMYQRSNHLP EKKTDILKEI VKHRKHLDGS
Ppa_gi168013224    HVLEFGDEEL DQYLGYDPIN QRDADLVHYW HRYHKSKVGS TAKAEAELRI LSHRMYIDKS
Ppa_gi168033758    HVMQYGDEEL ERYLGFDPVP QREADLVHLK HKFYNAKKGS LREANAASKT ILHRRHLDDS
Ppa_gi168005016    HVMQYGDEEL ARYLGYDPVD QREADLIHLR YKFRNAVKGS LREANAATKT IVHRKHLDDS
Ppa_gi168065024    HVMQYGDEEV ERYLGFDPVM QREAELLHLW HKYHKAVDGS -KKESAGMRT IAHRMHVDNS
Pab_MA_60392g0010  HVMQYGDEHL FLYIGSDPVN QRDADLLYLW QKYKRSKEGS IEKLESQKDL MTHRMHLDKS
Pab_MA_86205g0010  HVMQYGDDPL SLYIGFDPIN QRDADLLYLW QKYKRSKPDS TEKLEAQQES MSHRLYLDKS
Psi_gi148910236    HVMQYGNEEL YLYMGFDSIN QRDADLLYMW QKYKKSKEDS PERLTAQTEF MAHRMHVDKS
Psi_gi116789977    HVMQYGDDPL SLYIGFDPIN QRDADLLYLW QKYKRSKPDS TEKLEAQQES MSHRLYLDKS
Pta_000008627      HVMQYGNEEL YLYIGFDSIN QRDADLLYMW QKYKKSKEDS PERFTAQTEF MAHRMHVDKS
Pta_000069534      HVMQYGDEQL FLYIGSDPVN QRDADLLYLW QKYKRSKEGS IKKLESQRDL MTHRMHVDKS
Pta_000008629      HVMQYGDDPL SLYIGFDPIN QRDADLLYLW QKYKRSKPDS TEKLEAQQES MTHRLHLDKS
Ptr_006G232900     HVMQYGDDDL FQYMGTNPVN QRDADLVHFW TKYRKAPEGS SRKVEAQKEA MSHRMHIDHS
Ptr_gi566162122    HVMQYGNEKL YLYQGFNPVN QRDAELVFLW QMYKRSEDGS EKKTQILNET MRHRTQLDSS
Ptr_001G119800     HVMQYGNEKL FLYQGFDPVN QRDAELVFLW QMYKRAEGGS EKKTQILNET MRHRTHLDSS
Ptr_224141591      HVMQYGDDNI FLYMGTNPVN QRDADLVHFW DKYRKAPEGS SRKVEAQKEA MSHRMHIDHS
Ptr.008G003400     HVMQYGNELL SSYLGTNAFD QREATLLHFW HKYQEAPDGS DKKAEAHKRI HSHIRHVDRS
Ppe_gi462419182    HVMQYGDENL FVYMGTNPVN QRDADLVHFW HKYRKAPEGS PRKIQAQKEA MSHRMHMDQT
Ppe_gi462405686    HVMQYGSEKL YLYQGFDPVN QRDAELFFMW QMYKRSEHGS EKKTHILEET MGHRTHLDAS
```

Rco_gi255537021	HVMQYGNDKL	YLFQGFDPVN	QRDAELHFMW	QLYKRSENGS	EKKKEILQDA	IKHRSHLDSS
Rco_gi255550848	HVMQYGDENL	FLYMGTNPVN	QRDADLVHFW	DKYRKAPDGS	ARKDQAQKEA	MSHRMHIDHS
Shy_gi373254763	HVMQYGSQKL	FSYIGTNPCN	QRDADLVYFW	QKYRKLADGS	SKKNEARKEV	MSHRSHVDNS
Sof_gi93139442	HVMQYGSQKL	FSYIGTNPVN	QRDADLVYFW	QKYRKLADGS	SKKNEARKEV	MSHRSHVDNS
Smo_SM00084G00480	HVMQYGDNPL	SMFLGFDPVS	QRDADLLHLW	SKYRRAKDGS	DSKREARENA	LAHRQHVDES
Smo_SM00000G12900	HVMKYGDERL	SLYLGFDPIG	QRDADLLHFW	QKYKNSKENS	LEKSKALQDV	IGRRTQIDRS
Sin_gi9622221	HVMEYGNEKL	YLYQGFDPVN	QRDADLLFLW	ERYKRLDGGA	KKKSELFKDT	MLHRKHMDDS
Sit_gi514715547	HVMEYGDEKL	YLYQGFNPVN	QRDADILFLW	KRYELLNEKS	KEKLEVLRGT	VTHRKHLDSS
Sit_gi514802965	HVMEYGDEKL	FLYQGFDPVN	QRDADLLFMW	KRYEQLDGGS	EEKLRALRET	VQHRKHLDSS
Sly_gi460400421	HVMEYGSEKV	YLYQGFDPVN	QRDADLLFLW	ERYKKLEDNS	LEKAKLRKET	MLHRQHLDGS
Stu_gi565378091	HVMEYGSEKV	YLYQGFDPVN	QRDADLLFLW	ERYKKLEDNS	LEKAKLRKET	LQHRQHLDGS
Sbi_gi242063120	HVMEYGDEKL	YLYQGFDPVN	QRDADILFLW	KRYELLHEKS	EEKQEVLRGT	VRHRKHLDSS
Sbi_gi242076602	HVMEYGDEKL	FLYQGFDPVN	QRDADLLFMW	KRYEQLNGES	VEKLRALIET	VQHRKHLDSS
Sbi_gi242053313	HVMQYGSQKL	FSYIGTNPVN	QRDADLVYFW	QKYRKLADDS	SKKNEARKEV	MAHRSHVDNS
Tha_gi312281859	HVMQYGDDKL	DLYMGTNPTN	QRDADLVHFW	DKYRKAPEGS	TRKTEAQKEA	MSHRLHVDNS
Tca_gi590677122	HVMEYGNEKL	CSYQGFDPVN	QRDADILFLW	HMYKNSEDGS	-KKTEILRET	IRHRIHLDGS
Tca_gi590573851	HVMQYGDDIV	FVYLGTNPVN	QRDADLVHFW	DKYRKAPDGS	VRKLEAQKEA	MSHRMHIDNS
Tmo_gi356463700	HVMEYGDEKL	YLYQGFNPIN	QRDADLLFLW	RRYELLHEKS	KEKGNVLRET	VTHRKHLDSS
Tmo_gi356463706	HVMQYGSEHL	FSYIGSNPVN	QRDADLVYFW	QKYRKLAESS	PEKNDARKEM	MGHRSHIDNS
Tur_gi474124510	HVMEYGDDML	FLYQGFDPIN	QRDADILFMW	KKYEQLNGGS	EEKQRALTET	VLHRKHLDSS
Vna_gi2414681	HVMQYGDEKL	YLYHGFDPVN	QRDAEILFMW	HMYQRLDHQT	EKKKDTLEET	VKHRNHLDGS
Vsa_gi3452551	HVTEYGDEKL	YLYHGFDPVN	QRDAEILFMW	QMYQRLDHQS	EKKRDILKET	VKHRNHLDGS
Vvi_gi442540375	HVMVYGNELL	YLYQGFDPIN	QRDADLLFLW	QRYKRSKAGS	EKK-EILKQT	MQHRVHLDQS
Vvi_gi296085092	HVMVYGNELL	YLYQGFDPIN	QRDADLLFLW	QRYKRSKADS	EKK-EILKQT	MQHRVHLDQS
Vvi_VV04G07860	HVMQYGDEDL	VLYMGTNPVN	QRDADLVHFW	DKFRKAPEGS	PRKAEAQKEA	MSHRTHIDHA
Vvi_VV09G09760	HVTQYGDEFL	FTYMGTNPAS	QYDAELLHFW	YKFHRAPEGS	TRKLEAQKRK	ISHRMHVDHS
Zma_gi413937993	HVMEYGDEKL	YLYQGFDPVN	QRDADILFLW	KRYELLHEKS	EEKQEVLRGT	VRHRKHLDNS
Zma_gi414586098	HVMEYGDEKL	SFYQGFDPVN	QRDADLLFMW	KRYEQLNGGT	EEKLRALIET	VQHRKHLDSS
Zma_gi226493414	HVMEYGDEKL	SFYQGFDPVN	QRDADLLFMW	KRYEQLNGGT	EEKLRALIET	VQHRKHLDSS
Zma_gi195624628	HVMQYGAQRL	FSYIGTDPVN	QRDADLVYFW	QKYRKLADSS	PPKSEARKEV	MAHRSHVDSS
Zma_ZM08G11940	HAMEYGDQSL	YTFMGSDDVS	QRDADLVYFW	RRYRRAAERT	PEKAEARTRA	VSRRSRVDSI
Zma_gi6634705	HVMQYGSKHL	FSYIGTNPVN	QRDADLVYFW	QKYRKLADSS	PEKNEARREV	MAHRSHVDSS

```
                       301
Mtr_1g016590           ---------- ---------- ---------- ---------- ---------- ----------
Mtr_7g032230           VHQISDLLFG EEKGSVMVHV RASGQPLVDN WDCLKTLKTY ESHCGTLSSY GRKYLRAFAN
Mtr_7g079140           IHLIGNILFG EKKSSMMSDL RSAGQPLIDD WNCLKILKTY ESHCGILSTY GRKYSRVFAY
Pda_gi672145835        INLIGKLIFG SENGPILNAV RPSGQALVDD WNCLKTMQVF QSYCGPLTQH GMRHMRAFAN
Egu_gi743827934        INLIGKLIFG SENGPILNAV RPSGQALVDD WNCLKTMQVF QTYCGPLTQY GMRHMRAFAN
Mac_gi695022010        IDLIGKLIFG SNSGPILRAV RPYGQALVDD WDCLKSMRSF ESHCGSLTQY GMKHMRAFAN
Mac_gi695070617        IDYIGNQIFG SEIGPILRAV RPSGQALVDD WECLKSMRAF ESHCGSLTQY GMKHMRAFAN
Atr_262_1              VDLIGRLLFG WDKGSVLGAK RPSGKALVDD WSCLKSMRAF EEKCGPLTQY GMKHMRAFAN
Atr_262_2              VDLIGRLLFG WDKGSVLGAK RPSGKALVDD WSCLKSMRAF EEKCGPLTQY GMKHMRAFAN
Atr_36_100             IELIGKLLFG SEKGPILKTV RTTGLPLVDD WDCLKAMRTF ETKCGSISQY GMKHMRSMAN
Asp_gi356463708        IDFIGKLLFG FENGPELQAV RPSGKPLVDD WDCLKRMRIF ESHCGSLTQY GMKHMRAFAN
Ata_gi356463714        IDFIGKLLFG FENGPELQAV RPSGKPLVDD WDCLKRMRIF ESHCGSLTQY GMKHMRAFAN
Ata_gi475545141        VELIGNLLFG SAGGPVLKAV RPAGEPLVDD WSCLKSTRTF ESQCGSLAQY GMKHMRSFAN
Aly_gi297825691        ILLIGKLLFG LD-SPVLNNV RPSGTPLVDD WDCLKSLRVF EMHCGSLSQY GIKHMRSIAN
Aly_gi297798670        VILVGKILFG ISEGPVLNKV RSAGQPLVDD WNCLKNLRAF ERHCGSLSQY GIKHMRSFAN
Ath_gi334185489        ITDILRLSVK QTNVLLLTST RTTGQPLVDD WDCFKTLNSF KNHCGATVHY GLKYTGALAN
Ath_gi6630462          VELIATILFG PTMN-VLNLV REPGLPLVDD WE-LKSMRVF EEHCGSLTQY GMKHMRAFAN
Ath_gi15233996         VILVGKILFG ISRGPVLNKV RSAGQPLVDD WNCLKNQRAF ERHCGSLSQY GIKHMRSFAN
Ath_gi15225226         ILLIGILLFG LE-GHVLNKV RPSGEPLVDD WDCLKSLRAF ERHCGSLSQY GIKHMRSIAN
Adi_gi347326466        VKLIGNLLFG TEKGPLLSAV RPAGKPLVDD WDCLKNMRTF ETHCGSLSQY GMKHMRTFAN
Bdi_gi357136769        VDLIGQLLFG FENGPVLQAV RPSGKPLVDD WDCLKRMRIF ESHCGPLTQY GMKHMRAFAN
Bdi_gi357135238        VELTGNLLFG SEDGPVLKTV RTAGEPLVDD WGCLKSTRAF ESQCGSLAQY GMKHMRSFAN
Cen_gi1346432          VELIGVLLYG PGKSSVLHSV RAPGLPLVDD WTCLKSMRVF ETHCGSLTQY GMKHMRAFGN
Cru_gi565477221        VLLIGKLLFG LE-GPVLNKV RPSGRPLVDD WDCLKSMRAF ERHCGSLSQY GIKHMRSIAN
Cru_gi565489031        VELIDTILFG PAMN-VLNSI REPGLPLVDD WECLKSTRVF ETHCGSLTQY GMKHMRAFAN
Car_gi502175795        VELVGVLLFG PEKGSVLQSV RAPGLPLVDD WECLKSRRVF ETHCGSLTQY GMKHMRSFAN
Car_gi502132679        IKLVGKLLFG MEKGPVLTSV RPAGQPLADD WNCLKTLRTF ETYCGSLSQY GMKHMRSFAN
Ccl_gi567895782        IDMIGVILFG PDKGSILNSV GARGLPLVDD WQCLKSMRVF ETHCGSLTQY GMKHMRAFAN
Ccl_gi568846845        IDMIGVILFG PDKGSILNSV RARGLPLVDD WQCLKSMRVF ETHCGSLTQY GMKHMRAFAN
Csa_gi449459758        IRMIGFLLFG PEKGSILDDV RASGLPLVDD WECLKSMRVL ESYCGSLTQY GMKHMRAIAN
Esa_gi567127595        VELIGMILFG PTTN-VLNSV REPGLPLVDD WECLKSMRVF ETHCGSLTQY GMKHMRAFAN
Esa_gi567217560        VKLVGKLLFG ISEGPVLNKV RSAGQPLVDD WNCLKNLRAF ERHCGSLSQY GIKHMRSFAN
Fve_gi470125355        IKFIGTFLYG PKNGAVLNSV RPLGLPLVDD WECLKSMRVF ETHCGSLTQY GMKHMRAFAN
Gau_gi527209183        VETIGFILFG PAN--DLKST RSQGLPLVDD WDCLKSMQVF EERCGSLTQY GMKHMRAFAN
Gma_gi351724961        VKLIGKLLFG IEKGPVLNAV RPAGSALVDD WHCLKTMRTF ETHCGSLSQY GMKHMRSFAN
Gma_gi356564077        VKLIGKLLFG IEKGPVLNAV RPAGSALVDD WHCLKTMRTF ETHCGSLSQY GMKHMRSFAN
Gma_gi571453325        VELIGVLLYG PGKGSVLQSM RAPGLALVDD WTCLKSMRVF ETHCGTLTQY GMKHMRAFAN
Gma_gi351720847        VELIGVLLYG PGKGSVLQSV RAPGSSLVDD WTCLKSMRVF ETHCGTLTQY GMKHMRAFAN
Hvu_gi194352742        VELIGNLLFG SAGGPVLKTV RPAGEPLVDD WSCLKSTRTF ESQCGSLAQY GMKHMRSFAN
Hvu_gi194352740        IDFIGKLVFG FDKGPMLQAA RGSGQPLVDD WDCLKRMRVF ESQCGSLTQY GMKHMRAFAN
Hvu_gi194352736        IDFIGKLLFG FENGPMLETV RPSGIPLVDD WDCLKRMRIF ESHCGSLTQY GMKHMRAFAN
Hvu_gi313660970        IDFIGKLVFG FDKGPMLQAA RGSGQPLVDD WDCLKRMRVF ESQCGSLTQY GMKHMRAFAN
Hvu_gi313660968        IDFIGKLVFG FENGPVLEAA RSSGQPLVDD WDCLKRMRVF ESQCGSLTQY GMKHMRAFAN
Msi_gi443501900        MKLIGKLLFG IEKGPVLNAV RPAGQPLVDD WDCLKTMRSF ETHCGSLSQY GMKHMRSLAN
Mtr_gi357476961        VELIGVLLFG PTKGSVLQAV RATGLPLVDD WECLKSRRLF ETHCGSLTQY GMKHMRAFAN
Mtr_gi217074670        ------LLF- ---------- ---------- ---------- ---------- ----------
Mtr_gi357465673        IKLVGKLLFG MKKGPVLASV RPAGQPVVDD WDCLKSLRTF ETYCGSLSQY GMKHMRSFAN
Mno_gi587864848        IELIGSLLYG PQKGSVLNSV RSPGQPLVDD WACLKSMKSF ESHCGSLTQY GMKHMRAFAN
Nta_gi40809676         VDAIGVFLFG PTKGSVLNSV REPGLPLVDD WDCLKSTRLF ELHCGSLTQY GMKHMRAFAN
Obr_gi573916715        VELIGNLLFG SEEGPVLKAV RATGEPLIDD WSCLKSMRAF EAQCGSLAQY GMKHMRSFAN
Obr_gi573919988        VDFVGKLLFG FGNGPVLQHV RPSGQPLVDD WDCLKRMRIF ESHCGSLTQY GMKHMRAFAN
Osa_gi26006020         VDLVGKLLFG FGNGPVLQAV RPSGQPLVDD WDCLKRMRIF ESHCGPLTQY GMKHMRAFAN
Osa_gi115437636        VELIGNLLFG SEEGPVLKAV RATGEPLVDD WSCLKSMRTF EAQCGSLAQY GMKHMRSFAN
Osa_gi115465809        VEMIGGLLLG GAKHK--QVV RERA-ALVED WECLRSMRTF EDQCGSLGQY GIKHMRSFAN
Osa_gi6907094          VDLIGDILLG DSSKKLLHIR RPAGQPLVDD WDCLKSMRTF EAHCGPLGQY GMKHTRAFAN
Osa_gi38567871         IDFIGKLVFG FENGPALEAA RSSGQPLVDN WDCLKKMRIF ESQCGSLTQY GMKYMRAFAN
Prh_gi310771866        IKLIGKLLFG FEKGPVLEAV RPAGQPLVDD WDCLKTMRTF EAQCGSLSQY GMKHMRSVAN
Pvu_gi48429177         VELVGKLLFG IEKAPLLNAV RPAGSALVDD WDCLKTMRTF ETHCGSLSQY GMKHMRSFAN
Pvu_gi561009896        MKLIGKLLFG IEEGPLLNSV RPAGQPLVDD WDCLKTLRTF ETHCGSLSQY GMKHMRSFAN
Pvu_gi561028978        VELIGVLLYG PEKASVLRSV RTTGLPLVDD WTCLKSMRVY ETHCGSLTQY GMKHMRAFAN
Ppa_gi168013224        VDLVGRLLFG VEAGPTLSAV RPDGLPLTDD WACLKSMSAF ELSCGELSEY GMKHMRAFAN
Ppa_gi168033758        VRLIGELLFA GEDALKLGAV RPAGSVVVDD WACLKNMRIF EASCGPLTQY GMKHMRAFAN
Ppa_gi168005016        VQLIGEILFA GENALKLTAV RPAGSVVVDD WACLKTMRTF EASCGPLTQY GMKHMRAFAN
Ppa_gi168065024        IKLIGDHMFG LDTSLRLKAV RPAGQVLVDD WSCLKAMRTF EASCGPLTQY GMKHMRAFAS
Pab_MA_60392g0010      VNLIGKLLFG SVRGLVLNTV RPPGQPLVND WDCLKTMRTF EKHCGSLSQY GMKHMRSLAN
Pab_MA_86205g0010      INFIGKILFG SDTGTVLNAV RPSGQPLVDD WDCLKTMRTF ESHCGSLSQY GMKHVRALAN
Psi_gi148910236        VKLVGNLLFG PEKGPVFNAV RPQGEPLVDD WDCLKKMRTF EGHCGSLAQY GMKHMRALAN
Psi_gi116789977        INFIGKLLFG SDMGTVLNAV RPSGQPLVDD WDCLKTMRTF ESHCGSLSQY GMK-------
Pta_000008627          VKLVGNLLFG PEKGSVLKAV RPQGEPLVDD WDCLKKMRTF ERHCGSLAQY GMKHMRALAN
Pta_000069534          MNLIGKLLFG SARGPVLNTV RPPGQPLVDD WDCLKTMRTF EKHCGSLSQY GMKHMRSLAN
Pta_000008629          INFIGKLLFG SEMGTVLKAV RPSGQPLVDD WDCLKTM--- ---------- ----------
Ptr_006G232900         IKLIGKLLFG IEKASALNTV RPAGQPLVDD WVCLKTLRTF ETHCGSISQY GMKHMRSLAN
Ptr_gi566162122        MELIGTLLFG RKKGSILKSV REPGSPLVDD WICLKSMRRF ETHCGSLTQY GMKHMRAFAN
Ptr_001G119800         MELIGTLLLG PKKGSILKSV REPDSPLVDD WRCLKSMRLF ETHCGSLTQY GMKHMRAFAN
Ptr_224141591          IKLIGKLLFG IEKASVLNAI RPAGQPLVDD WDCLKTLRTF ETHCGSVSQY GMKHMRSLAN
Ptr.008G003400         LSHIASTLFG DENAAAMKHV RPSGQPLVDD WDCLKGLEAY EKQCGGLSWY GKKYTRVIAN
Ppe_gi462419182        MKLIGKLLFG IKKGPVLNTV RPAGQPLVDD WDCLKTMRSF ETYCGSLSQY GMKHMRSLAN
Ppe_gi462405686        IEFIGTFLYG PGKGFTLNSV RALGLPLVDD WECLKSMRVF ETQCGLLTQY GMKHMRAFAN
```

Rco_gi255537021	MQLIGDLLFG	PKKASILKSV	REPGSPLVDD	WGCLKSMRVF	ETCCGSLTQY	GMKHMRTFAN
Rco_gi255550848	VKLIGKLLFG	LEKASVLSTV	RPAGQPLVDD	WDCLKKLRTF	ETHCGSISQY	GMKHMRSLAN
Shy_gi373254763	VELIGSLLFG	SEDGPVLKAV	RAAGEPLVDD	WSCLKSMRTF	EAQCGSLAQY	GMKHMRTFAN
Sof_gi93139442	VELIGSLLFG	SEDGPVLKAV	RAAGEPLVDD	WSCLKSMRTF	EAQCGSLAQY	GMKHMRTFAN
Smo_SM00084G00480	VDRVGERLFG	SKAAAVLSTV	RGSGLALVDD	WTCLKSLQAF	ETSCGLLGQY	GMKHMRAFAN
Smo_SM00000G12900	VELVGSVLLG	SESASILNSV	RPEGHPLVDN	WDCLKEMRVF	ETKCGPLGQY	GMKHMRAFAN
Sin_gi9622221	IDIIGAFLFG	PENGPILKSV	RDRGLPLADD	WDCLKSMRLF	EAHCGSLTQY	GMKHTRAFAN
Sit_gi514715547	VDFIGKLLFG	VENGPTLGAV	RSPGQPLVDD	WDCLKRMRIF	ESHCGSLTQY	GMKHMRAFAN
Sit_gi514802965	IDFIGRLVFG	FENGPMLEAV	RASGQPLVDD	WDCLKRMRIF	EAQCGSLTQY	GMKYMRAFAN
Sly_gi460400421	IDAVGVFLFG	PIKGGVLSSV	RKPGLPLVDD	WECLKSTRLF	EAHCGSLTQY	GMKHMRAFAN
Stu_gi565378091	IDAVGVFLFG	PIKGGVLSSV	RKPGLPLVDD	WECLKSTRLF	EAHCGSLAQY	GMKHMRAFAN
Sbi_gi242063120	IDFIGKLLFG	IEKGPTLQAV	RPSGQPLVDD	WDCLKQMRIF	ESHCGSLTQY	GMKHMRAFAN
Sbi_gi242076602	IDFIGRLLFG	FEKGPMLEAV	RASGLPLVDD	WDCLKRMRIF	ESQCGSLTQY	GMKYMRAFAN
Sbi_gi242053313	VELIGSLLFG	SEDGPVLKAV	RAAGEPLVDD	WSCLKSMRTF	EAQCGSLAQY	GMKHMRSFAN
Tha_gi312281859	VKLVGKLLFG	ISEGPVLNKV	RSAGQPLVDD	WNCLKNLRAF	ERHCGSLSQY	GIKHMRSFAN
Tca_gi590677122	IDLIGTLLYG	PAKGSILNSV	REPGLPLVDD	WQCLKSMRLF	ETHCGLLTQY	GMKHMRAFAN
Tca_gi590573851	MKLIGKLLFG	IEKGPVMKTV	RPAGQPLVDD	WKCLKKMRTF	ETHCGSLAQY	GMKHMRSLAN
Tmo_gi356463700	IDFIGKLLFG	FENGPVLQAV	RPSGKPLVDD	WDCLKRMRIF	ESHCGSLTQY	GMKHMRAFAN
Tmo_gi356463706	VELIGNLLFG	SAGGPVLKAV	RPAGEPLVDD	WSCLKSTRTF	ESQCGSLAQY	GMKHMRSFAN
Tur_gi474124510	IDFIGKLVFG	FEKGPVLDAA	RGSGQPLVDD	WDCLKTMRIF	ESQCGSLTQY	GMKHMRAFAN
Vna_gi2414681	VELIGVLLFG	PTKGSVLQSV	RASGLPLVDD	WECLKSRRVF	ETHCGSLTQY	GMKHMRAFAN
Vsa_gi3452551	VELIGVLLFG	PTRGSVLQSV	RASGLPLVDD	WECLKSRRVF	ETHCGSLTQY	GMKHMRAFAN
Vvi_gi442540375	IELIGMLLLG	PENGPLLNAV	RPRGLPVVDD	WECLKSMVVF	ETRCGSLTQY	GMKHMRAFAN
Vvi_gi296085092	IELIGMLLLG	PENGPLLNAV	RPRGLPVVDD	WECLKSMVVF	ETRCGSLTQY	GMKHMRAFAN
Vvi_VV04G07860	IKLVGRLLFG	MKKGSVLKTV	RPAGQPLVDD	WHCLKTLRTF	EAHCGSLSQY	GMKHMRSIAN
Vvi_VV09G09760	MKEIGKLILG	SENSTLLKTV	RPLDQPVVDD	WDCYKMLKTY	EEHCGSLSRY	GLKYTRALAN
Zma_gi413937993	INFIGKLLFG	AEKGPTLEAV	RPPGQPLVDD	WDCLKQMRIF	ESHCGSLTQY	GMRHMRAFAN
Zma_gi414586098	IDFVGRLVFG	FEKGPMLEAV	RTSGQPLVDD	WDCLKRMRIF	ESQCGSLTQY	GMKYMRAFAN
Zma_gi226493414	IDFVGRLVFG	FEKGPMLEAV	RTSGQPLVDD	WDCLKRMRIF	ESQCGSLTQY	GMKYMRAFAN
Zma_gi195624628	VELIGSLLFG	SEDGPVLKAV	RAPGEPLVDD	WSCLKSIRTF	EARCGSLAQY	GMKHMRSFAN
Zma_ZM08G11940	MELIGGLLFG	SEGGPVLGAV	RPAGQPLADD	WDCLKSLRAY	ERSCGPLGQY	GMKHMRGFAN
Zma_gi6634705	VELIGSLLFG	SEDGPVLKAV	RAAGEPLVDD	WSCLKSTRTF	EAQCGSLAQY	GMKHMRSFAN

```
                    361
Mtr_1g016590        ---------- ---------
Mtr_7g032230        MCNNGITVKQ MVAASLQAC
Mtr_7g079140        MCNIGIFEKQ TISAVSQVC
Pda_gi672145835     LCNRGISNDA MVEACVNTC
Egu_gi743827934     ICNRGSSKDA MVEACLNAC
Mac_gi695022010     ICNRGISRDA IKEASASAC
Mac_gi695070617     ICNEGISKDV MEAACSRSC
Atr_262_1           ICNEGISLEV MSKACEEVC
Atr_262_2           ICNEGISLEV MSKACEEVC
Atr_36_100          ICNAGISKEV MAEASAEAC
Asp_gi356463708     ICNNGVSGTT MNEASIGAC
Ata_gi356463714     ICNNGVSGTT MKEASINTC
Ata_gi475545141     ICNAGIVPEA MAKVAAQAC
Aly_gi297825691     ICNAGIQMGQ MEEAAMQAC
Aly_gi297798670     ICNAGIRTEQ MEEAASQAC
Ath_gi334185489     ICNMGVDVKQ TVSAIEQAS
Ath_gi6630462       VCNNGVSKEL MEEASTAAC
Ath_gi15233996      ICNAGIQMEQ MEEAASQAC
Ath_gi15225226      MCNAGIQMRQ MEEAAMQAC
Adi_gi347326466     ICNAGIHKDQ MDEATAQAC
Bdi_gi357136769     ICNNGIPGST MKEGSISAC
Bdi_gi357135238     ICNAGILPEA TAKVAAQAC
Cen_gi1346432       VCNSGVSKAS MEEACKAAC
Cru_gi565477221     ICNAGIQMGL MEEAAKQAC
Cru_gi565489031     VCNNGISKEL MEEASTAAC
Car_gi502175795     ICNNDISETS FEEACLKAC
Car_gi502132679     FCNAGIHKEQ MAEASAQAC
Ccl_gi567895782     ICNSGVSQAL MEETSEAAC
Ccl_gi568846845     ICNSGVSQAL MEETSEAAC
Csa_gi449459758     ICNRGVSKAS MREASMVAC
Esa_gi567127595     VCNNGVSKEL MEKASAAAC
Esa_gi567217560     ICNAGIQMEQ MEEASSQAC
Fve_gi470125355     ICNSGVSQAQ MEEASWAAC
Gau_gi527209183     ICNNGVTTED MAEASAVAC
Gma_gi351724961     ICNVGIKNEQ MAEASAQAC
Gma_gi356564077     ICNVGIKNEQ MAEASAQAC
Gma_gi571453325     ICNSGVSEAS MEEVCVAAC
Gma_gi351720847     ICNSGVSEAS MEEACLAAC
Hvu_gi194352742     MCNAGIVPEA MAKVAAQAC
Hvu_gi194352740     ICNNGVSEAE MKEASISAC
Hvu_gi194352736     ICNNGISGTS MKEASISTC
Hvu_gi313660970     ICNNGVPEAE MKEASINAC
Hvu_gi313660968     ICNNGVSEAQ MREASISAC
Msi_gi443501900     ICNAGMTQDQ MAEASAQAC
Mtr_gi357476961     ICNSGISEDS MEKACMVAC
Mtr_gi217074670     ---------- ---------
Mtr_gi357465673     FCNAGIHSEQ MAEASAQAC
Mno_gi587864848     ICNSGVSVAS MEEAALAVC
Nta_gi40809676      ICNNGVSRDA MEEAFMAAC
Obr_gi573916715     ICIAGISAEE MAKVAAQAC
Obr_gi573919988     ICNNGIPDAA MKDASIVAC
Osa_gi26006020      ICNNGISGAS MKEASIATC
Osa_gi115437636     ICNAGISAEA MAKVAAQAC
Osa_gi115465809     ICNAGVPHHA MAKAASLAC
Osa_gi6907094       MCNAALDHNH MAKAASKAC
Osa_gi38567871      ICNNGVSEAK MMEASINAC
Prh_gi310771866     ICNAGIKKEQ MAEAASQAC
Pvu_gi48429177      MCNVGIKKEQ MREASAQAC
Pvu_gi561009896     FCNAGIGKEQ MAEASAQAC
Pvu_gi561028978     ICNSGVSETS MEKACVAAC
Ppa_gi168013224     ICNAGVEPSK MSGVAAEAC
Ppa_gi168033758     ICNAGINSSR MSLASLEVC
Ppa_gi168005016     ICNARIDPAK MAVASSEAC
Ppa_gi168065024     ICNAGIDLDT MKKATSQAC
Pab_MA_60392g0010   ICNEGVTKNT MAVVSAEAC
Pab_MA_86205g0010   ICNNGVSVDT MAEVSAEAC
Psi_gi148910236     ICNEGISMDT MATVSAEAC
Psi_gi116789977     ---------- -----THAC
Pta_000008627       ICNEGISMDT MATVSAEAC
Pta_000069534       MCNEGVTKNT MTAVSAEAC
Pta_000008629       ---------- IFKCSMVIC
Ptr_006G232900      LCNAGIVKEQ MAEASAQAC
Ptr_gi566162122     ICNGGVSQAS MEEACIAAC
Ptr_001G119800      ICNGGVSLAS MEEACVAAC
Ptr_224141591       LCNAGIGKEQ MAEASAQAC
Ptr.008G003400      MCNAGINVEQ MIGASTRAC
Ppe_gi462419182     ICNAGMTKEQ MTEASAQAC
Ppe_gi462405686     ICNSGVTQSE MEEACSAAC
```

Rco_gi255537021	ICNAGVSHTS	MEEACNAAC
Rco_gi255550848	LCNAGIREEQ	MAEASAQAC
Shy_gi373254763	ICNAGILPEA	VSKVAAQAC
Sof_gi93139442	ICNAGILPEA	VSKVAAQAC
Smo_SM00084G00480	LCNEGVDVPR	MAAASAEAC
Smo_SM00000G12900	LCNAGVDPER	MKSAAGATC
Sin_gi9622221	ICNSRVSSAD	MEDACMAAC
Sit_gi514715547	ICNRGTPATA	MKEASISAC
Sit_gi514802965	ICNSGISEAK	MRESSISAC
Sly_gi460400421	ICNNGISSDA	MEDAFMAAC
Stu_gi565378091	ICNNGISRDA	MEEAFMAAC
Sbi_gi242063120	ICNSGTPGAS	MKQASMGAC
Sbi_gi242076602	ICNSGISEMK	MRESSISAC
Sbi_gi242053313	ICNAGILPEA	VSKVAAQAC
Tha_gi312281859	ICNAGIQMEQ	MEEASSQAC
Tca_gi590677122	LCNSGVSQSL	MEQACVAAC
Tca_gi590573851	ICNAGIQTEQ	MAEASAQAC
Tmo_gi356463700	ICNNGISGTT	MKEASIGAC
Tmo_gi356463706	ICNAGIVPEA	TAKVAAQAC
Tur_gi474124510	ICNNGVSEAE	MKEASISAC
Vna_gi2414681	ICNSGISEDS	MEEACMAAC
Vsa_gi3452551	ICNRGISEDL	MEETCMVAC
Vvi_gi442540375	ICNNGISLTA	MEEACRSAC
Vvi_gi296085092	ICNNGISLTA	MEEACRSAC
Vvi_VV04G07860	ICNAGIEKEQ	MAEASAQAC
Vvi_VV09G09760	MCNAGIKMEQ	MAVASAQAC
Zma_gi413937993	ICNSGTPGAS	MKRASMGAC
Zma_gi414586098	ICNSGISEMK	MRESSISAC
Zma_gi226493414	ICNSGISEMK	MRESSISAC
Zma_gi195624628	MCNAGILPEA	VSKVTAQAC
Zma_ZM08G11940	ICNAGVGEDG	MAKVASEAC
Zma_gi6634705	ICNAGILPEA	VSKVAAQAC
