# Supplementary material for: The Amborella vacuolar processing enzyme family
Source: Front Plant Sci. 2015 Aug 21;6:618. doi: 10.3389/fpls.2015.00618 (PMC4544213; doi:10.3389/fpls.2015.00618)

Supplemental Figure S4: SNP distributions

These distributions are shown for the three *Amborella* VPE genes *AmTr_36.10 0* (referred to as 100), *AmTr_261-1* (referred to as 262-1) and *AmTr_262-2* (referred to as 262-2) within 12 individuals (See Map in Figure 3).

A) Minor allele frequency (MAF), exons are in red, SNPs in exons are in blue

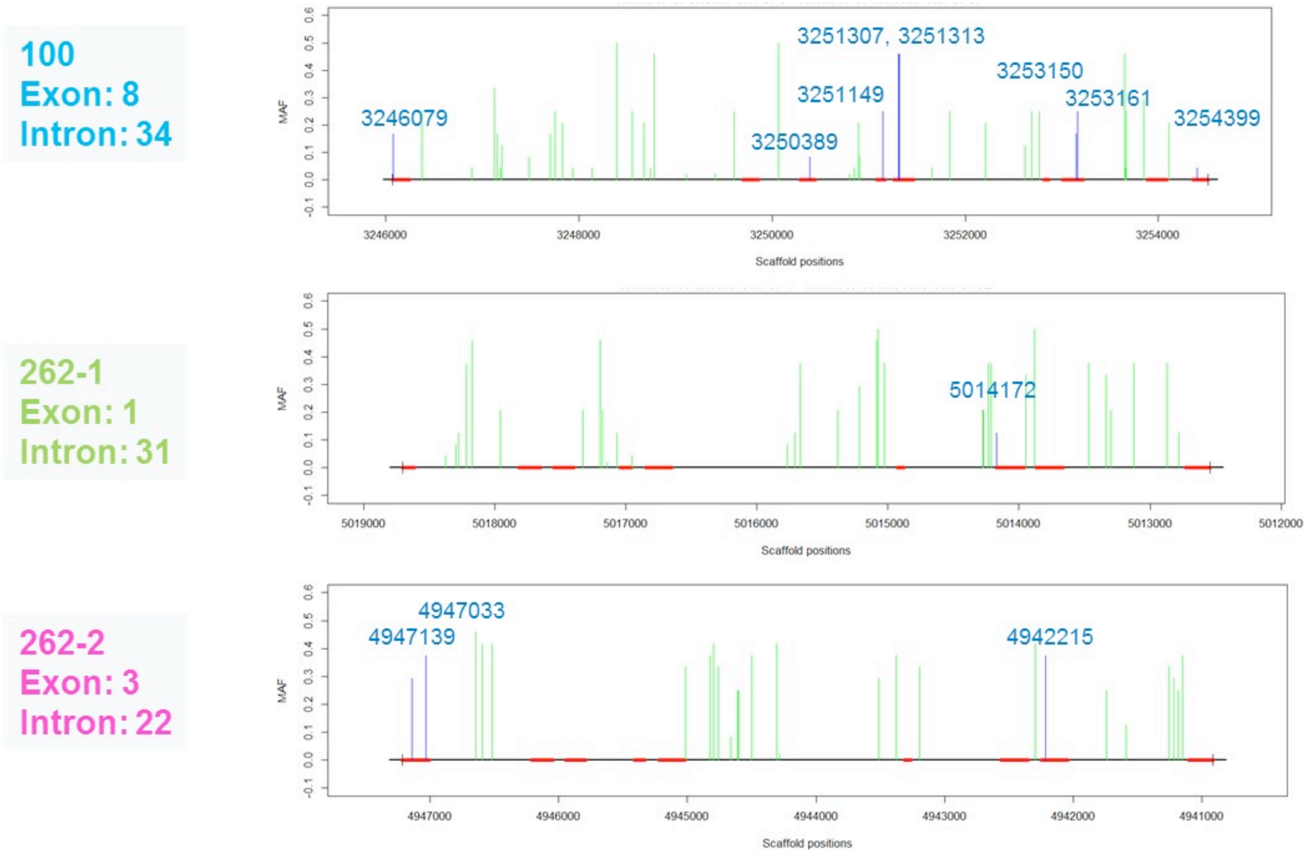

B) Impact of exonic mutations

Exonic mutations were characterized for the three *Amborella* VPE genes *AmTr_36.100* (referred to as 100), *AmTr_261-1* (referred to as 262-1) and *AmTr_262-2* (referred to as 262-2) from 12 resequenced individuals (See Map in Figure 3).

SNP	Exon #	Ncl ref	Ncl alt	Aa #	Aa ref		Aa alt		Changes
**AmTr_262-1**									
5014172	7	T	G	290	GTA	Valine	GGA	Glycine	both non polar
**AmTr_262-2**									
4942215	8	G	C	418	GCG	Alanine	GCC	Alanine	synonyme
4947033	1	C	T	61	TCC	Serine	TTC	phenylalanine	polar-> non pola
4947139	1	T	C	26	TTT	phenylalanine	CTT	Leucine	both polar
**AmTr_100**									
3246079	1	T	C	4	TCT	Serine	TCC	Serine	synonyme
3250389	3	G	A	149	GGT	Glycine	AGT	Serine	nonpolar->polar
3251149	4	T	G	190	TCT	Serine	TCG	Serine	synonyme
3251307	5	T	C	211	GGT	Glycine	GGG	Glycine	synonyme
3251313	5	A	G	213	TTA	Leucine	TTG	Leucine	synonyme
3253150	7	C	T	327	CGC	Arginine	TGC	Cysteinz	basic->polar
3253161	7	G	A	330	TTG	Leucine	TTA	Leucine	synonyme
3254399	9	T	C	433	TAT	Tyrosine	TAC	Tyrosine	synonyme

C) Geographical distribution of the 12 resequenced *Amborella* genotypes, their intronic average minor allele frequency (MAF) for each of the three *Amborella* VPE genes *AmTr_36.100* (red), *AmTr_262-1* (blue) and *AmTr_262-2* (yellow) and their assignation to the four genetic clusters as inferred by SSR analysis (Poncet et al. 2013).

Genes *AmTr_36.100*, *AmTr_262-1* and *AmTr_262-2* are referred to as gene 100, gene 262-1 and gene 262-2, respectively. The names of the populations belonging to the “North” genetic cluster are in green; to the “Center” cluster in blue; to the “Me” cluster in yellow; to the “Nak” cluster in red.

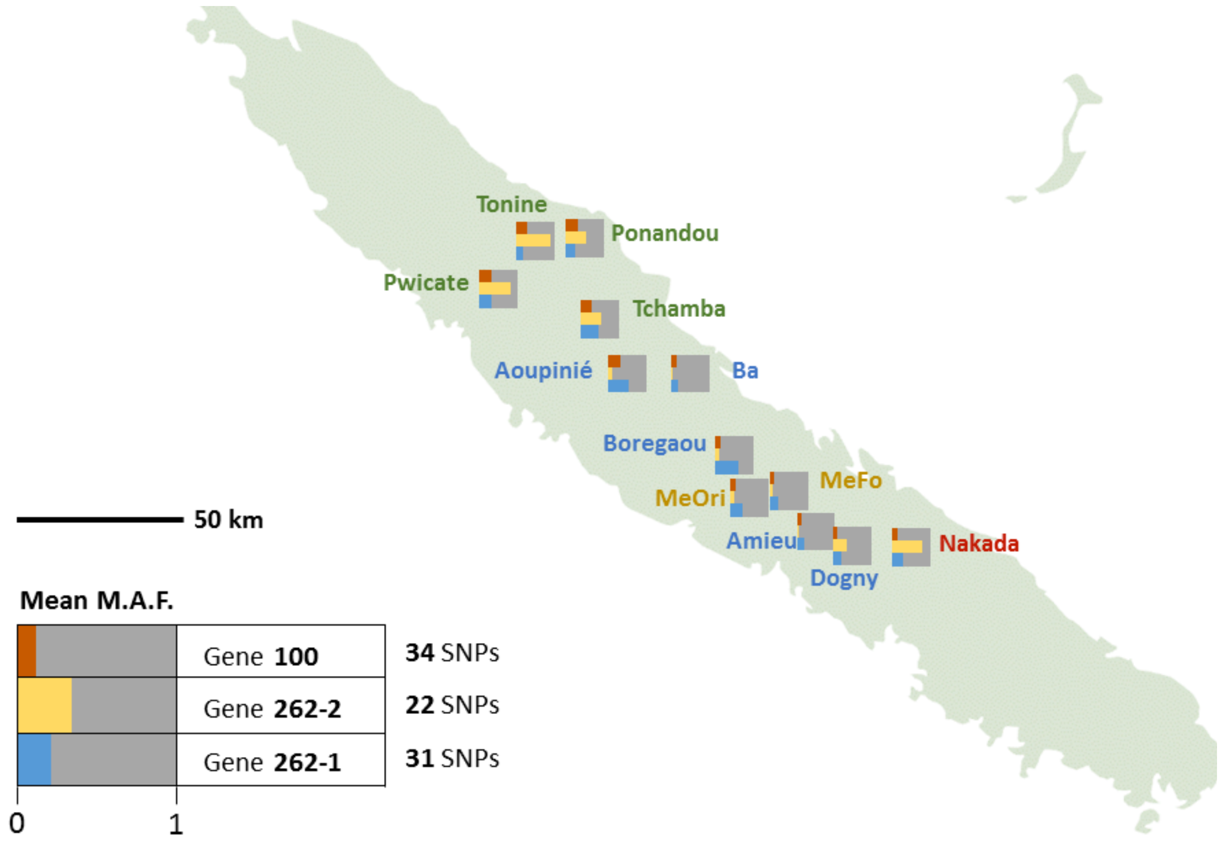

Supplement: Supplementary file 5 [file Image4.PDF]
